# The impact of circulating nucleosomes on inflammation in acute lung injury

**DOI:** 10.1096/fj.202401571RR

**Published:** 2024-11-29

**Authors:** Saugata Dutta, Yin Zhu, Sultan Almuntashiri, Payaningal R. Somanath, Shaheen Islam, Wenbo Zhi, Gustavo Ramírez, Nora Regino, María Jose Leyva‐Zilli, Victoria Muñoz‐Guido, Luis Jiménez‐Alvarez, Alfredo Cruz‐Lagunas, Tatiana S. Rodriguez‐Reyna, Joaquin Zuñiga, Xiaoyun Wang, Duo Zhang

**Affiliations:** ^1^ Clinical and Experimental Therapeutics, College of Pharmacy University of Georgia and Charlie Norwood VA Medical Center Augusta Georgia USA; ^2^ Division of Pulmonary, Critical Care & Sleep Medicine, Medical College of Georgia Augusta University Augusta Georgia USA; ^3^ Center for Biotechnology and Genomic Medicine, Medical College of Georgia Augusta University Augusta Georgia USA; ^4^ Laboratory of Immunobiology and Genetics and Intensive Care Unit Instituto Nacional de Enfermedades Respiratorias Ismael Cosío Villegas Mexico City Mexico; ^5^ Tecnologico de Monterrey School of Medicine and Health Sciences Mexico City Mexico; ^6^ Department of Immunology and Rheumatology Instituto Nacional de Ciencias Médicas y Nutrición Salvador Zubirán Mexico City Mexico

**Keywords:** ARDS, COVID‐19, extracellular histones, NF‐κB, organ crosstalk

## Abstract

Extracellular histones are released in two major forms: free histones and nucleosomes (DNA‐bound histones). However, little distinction has been made between these two forms of circulating extracellular histones. Our study detected increased circulating nucleosomes in acute lung injury patients. Further, our group identified nucleosomes as the leading form of extracellular histones compared to free histones in the plasma of COVID‐19 patients, underscoring the necessity to reassess the forms of circulating histones and nucleosome contributions to immunopathology. Functionally, nucleosomes activated macrophages and induced inflammation in different organs. Mechanistically, we observed nucleosomes activating the NF‐κB signaling, while inhibition of NF‐κB by sulfasalazine attenuated nucleosome‐induced macrophage activation. Taken together, our study indicates that extracellular histones are predominantly released as nucleosomes, playing a critical role in the inflammation of the lungs and other organs.

## Graphical abstract

Extracellular histones are predominantly released as nucleosomes during ALI. Extracellular nucleosomes can lead to macrophage activation and trigger inflammation in various organs. Mechanistically, nucleosomes activate the NF‐κB signaling pathway, while inhibition of NF‐κB by sulfasalazine attenuated nucleosome‐induced macrophage activation.

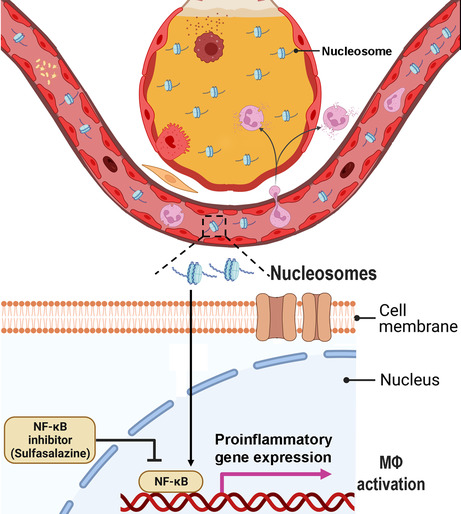

**Abbreviations**
ALI: acute lung injury
ARDS: acute respiratory distress syndrome
cf: circulating free
FACTT: Fluids and Catheters Treatment Trial
ELISA: enzyme‐linked immunosorbent assay
BAL: bronchoalveolar lavage
IT: intratracheal instillation
CFSE: carboxyfluorescein succinimidyl ester
BMDM: bone marrow‐derived macrophage
WT: wild type
ROS: reactive oxygen species
TEM: transmission electron microscope
LC‐MS/MS: Liquid Chromatography‐Tandem Mass Spectrometry
PSM: peptide‐spectrum match
EVs: extracellular vesicles
H&E: Haemotoxylin and Eosin
IV: intravenous injection
PaO2/FiO2: partial pressure of oxygen/fraction of inspired oxygen ratio

## 1 INTRODUCTION

Acute lung injury (ALI) is a life‐threatening disease process observed in acute respiratory distress syndrome (ARDS) and advanced COVID‐19 infection characterized by widespread inflammation.^1^ The pathogenesis of inflammatory damage in ALI is complex, and incompletely understood,^2^, ^3^ although attenuation of this inflammatory cascade is thought to be a key treatment target.

Extracellular histones are released from dying cells during cell death, including apoptosis, necrosis, NETosis, pyroptosis, and ferroptosis.^4^ Several limitations exist in current extracellular histone studies. First, histones can be released into the extracellular space in the forms of free histones and nucleosomes. However, little distinction between these two forms has been made. Second, although studies suggested that circulating nucleosomes might be a predictor for sepsis^5^ or pediatric ARDS,^6^ nucleosomes were quantified in arbitrary units rather than concentration, making it impossible for clinical use. Last, basic studies exclusively employed *E. coli*‐produced recombinant histones or calf thymus histones at suprapathological concentrations, which could lead to exaggerated results and outcomes.^4^, ^7^, ^8^, ^9^

Recent studies have provided evidence that the circulating extracellular nucleosomes are involved in the inflammatory progression of a variety of pathological conditions.^10^, ^11^ Particularly, circulating nucleosomes can trigger a substantial level of inflammation which can ultimately result in a cytokine storm.^12^ Circulating nucleosomes even have more pro‐inflammatory attributes than circulating free (cf) histones and cfDNA.^13^ Circulating nucleosomes are also being used as diagnostic biomarkers.^11^, ^14^ Despite indications of a strong association between circulating nucleosomes and lung inflammation, the specific role and the underlying mechanism of nucleosomes in ALI remain inadequately investigated.

In this study, we conducted regulatory and functional assessments and analysis of samples from ARDS and COVID‐19 patients to understand the contribution of circulating nucleosomes to inflammation and its associated mechanisms. The assessments included quantification of circulating nucleosomes, analysis of the pro‐inflammatory effect, and the relationship between nucleosomes and the NF‐κB signaling pathway. We hypothesized that circulating nucleosome is the leading form of extracellular histones compared to free histones in ALI/ARDS patients. Nucleosomes can promote inflammation and activate macrophages via the NF‐κB signaling pathway.

## 2 MATERIALS AND METHODS

### 2.1 Human specimens and ethics approval

This study includes a secondary analysis of a subset of the fluids and catheters treatment trial (FACTT) patients and COVID‐19 patients that contained five experiments: proteomics, Western blot analysis, immunogold labeling, enzyme‐linked immunosorbent assay (ELISA), and inflammatory mediators analysis. We used unidentifiable human plasma specimens for analysis in accordance with the Declaration of Helsinki. Human sample research was approved by the institutional review board at Augusta University (IRB reference number: 1889310‐1 and 2070085‐1) and determined this project was nonhuman subject research. The demographic information in each cohort is listed in Table 1.

**TABLE 1:** Different cohorts used in the study: Demographic and clinical characteristics.

	All (*n* = 94)	Normal (*n* = 20)	COVID‐19 (*n* = 60)	ARDS (*n* = 9)	BAL (*n* = 5)	*p* value
Age in years	49.83 ± 15.43	39.25 ± 10.5	50.3 ± 13	53.44 ± 19.7	80 ± 5	<.001
Male gender *n* (%)	41 (43.6)	5 (25)	30 (50)	3 (33.3)	3 (60)	.191

*Note*: The table shows the demographics of different cohorts that were used in the study. Data are presented as mean ± SD.

### 2.2 Isolation of extracellular particles from human plasma

The pellets from human plasma were prepared using the sequential centrifugation protocol described previously.^15^, ^16^ Plasma specimens were centrifuged at 2000 *g* for 10 min at 4°C dto remove cell debris and apoptotic bodies. The supernatant was centrifuged at 16 000 *g* for 40 min at 4°C to pellet circulating particles for further analysis.

### 2.3 Nucleosomes preparation and labeling

Human or murine nucleosomes were isolated from either BEAS‐2B or MLE 12 cells under normal culture conditions using the Nucleosome Preparation Kit (53504, Active Motif, Carlsbad, CA, USA) following the manufacturer's instructions. The digestion time was optimized to obtain mononucleosomes and dinucleosomes. To mitigate species‐related immunogenicity, we utilized species‐matched nucleosomes in the study. To track the interaction between nucleosomes and macrophages, purified nucleosomes were labeled with carboxyfluorescein succinimidyl ester (CFSE) fluorescence dye (C34554, Thermo Fisher Scientific, Waltham, MA, USA) according to the manufacturer's protocol. Unincorporated dye was removed using a 3 kDa Amicon Ultra Centrifugal Filter (UFC500308, MilliporeSigma, Rockville, MD, USA). Fluorescence images were captured using a Carl Zeiss Observer Z1 microscope.

### 2.4 Haemotoxylin and Eosin (H&E) staining and Hema 3 staining

H&E staining of lung sections was conducted in the Histology Core Laboratory at Augusta University. Bronchoalveolar lavage (BAL) cells were cytocentrifuged at 500 *g* for 5 min using a Shandon Cytospin 4 (Thermo Fisher Scientific) and concentrated onto a microscope slide. Cells were air‐dried and stained with PROTOCOL Hema 3 fixative and solutions (22‐122911, Thermo Fisher Scientific). Images were captured using a Zeiss Observer Z1 microscope.

### 2.5 Concentration of nucleosome and nucleosome histone H3

The concentration of nucleosome histone protein was measured using the absorbance at 230 nm. The average molecular weight of a nucleosome is approximately 200 kDa and it consists of approximately 50% proteins and 50% DNA. Thus, the concentration of nucleosome was twice the concentration of nucleosome histone protein. The molecular weight of histone H3 is about 15 kDa. Based on the structure of nucleosomes, histone H3 accounts for approximately 15% of the mass of nucleosomes. Therefore, there is approximately 150 ng histone H3 in 1 μg nucleosome.

### 2.6 Animal studies

C57BL/6J wild‐type (WT) mice (000664, Jackson Laboratory, Bar Harbor, ME, USA) of both sexes (8 weeks old) were purchased and anesthetized with 2% isoflurane (008957, Covetrus, Portland, ME, USA). Fifty microliters of sterile phosphate‐buffered saline (PBS) (10010023, Thermo Fisher Scientific) with or without 50 μg murine nucleosomes (*n* = 5–7 per group) was administered to mice via intratracheal instillation. The nucleosome dose was chosen based on the previous ALI study in mice.^9^ The mice were euthanized 24 h after treatment and whole lung tissues were collected for RNA extraction or fixed using 4% paraformaldehyde (J61899.AK, Thermo Fisher Scientific) for histological analysis. For other experiments, BAL was collected as previously described.^17^ The total BAL cells were counted using a hemocytometer. To investigate the systemic effects of nucleosomes, BALB/cJ mice (000651, Jackson Laboratory) of both sexes (8 weeks old) were used for the following experiments. Three hundred microliters of sterile PBS with or without 300 μg murine nucleosomes (*n* = 4–5 per group) was administered to mice via intravenous injection (IV). The mice were euthanized 24 h after treatment and kidney, liver, and lungs were collected for RNA extraction. All the animal experiments were approved by the Charlie Norwood Veterans Affairs Medical Center Institutional Animal Ethics Committee and followed the ARRIVE guidelines.

### 2.7 Cell culture, transfection, and NF‐κB reporter assay

U937 (CRL‐1593.2), MH‐S (CRL‐2019), BEAS‐2B (CRL‐3588), MLE 12 (CRL‐2110), HUVEC (CRL‐1730), and HEK293T (CRL‐3216) cells were obtained from the American Type Culture Collection (ATCC) (Manassas, VA, USA) and cultured according to the standard protocol provided by ATCC. All cell lines were tested for mycoplasma contamination. Human BAL cells were isolated from the BAL cohort and cultured as previously described.^18^ Murine bone marrow‐derived macrophages (BMDM) were isolated from C57BL/6J WT mice and cultured as we described previously.^17^ Approximately 2 × 10^6^ murine alveolar macrophages were enriched from BAL cells using the protocol previously described.^19^ Briefly, the murine BAL cells were collected following an adhesion step. Differential staining was conducted using PROTOCOL Hema 3 solutions to ensure the enrichment of alveolar macrophages.

### 2.8 Co‐culture model

Corning Transwell 24 well plates (#3413) with 0.4 μm pore size, tissue culture (TC)‐treated surface round wells were used to establish the co‐culture system. Differentiated U937 cells were incubated with or without 4 μg/mL nucleosomes for 6 h before being seeded in the insert with 0.1 mL culture medium. Meanwhile, 5 × 10^4^ BEAS‐2B or HUVEC cells with 0.6 mL culture medium per well were seeded in the 24‐well plate and incubated for 2 h to allow cell adhesion. Then the transwell membrane with untreated or nucleosome‐treated U937 was inverted and placed on the well. The co‐cultured cells were incubated for 24 h. ROS production and pro‐inflammatory markers were determined in BEAS‐2B and HUVEC.

### 2.9 Reactive oxygen species (ROS) detection

ROS generation was detected as previously described.^20^ Briefly, 5 μM CellROX Green dye (Thermo Fisher Scientific) was added to the cultured cells for 30 min to determine the cellular oxidative stress. Cells were washed with PBS three times and then fixed with a 4% formaldehyde solution (MilliporeSigma) for 1 h before imaging. The nuclei of the cells were stained using 4′,6‐diamidino‐2‐phenylindole (DAPI, Abcam, Waltham, MA, USA). Images were taken using a Carl Zeiss Observer Z1 microscope (Jena, Germany). The fluorescence intensity of these images was quantified using ImageJ software (U.S. National Institutes of Health, Bethesda, MD, USA).

### 2.10 Liquid Chromatography‐Tandem Mass Spectrometry (LC‐MS/MS) analysis

For LC‐MS/MS, the pellets were collected from 400 μL of pooled plasma specimens from normal individuals and ARDS patients (*n* = 4 per group, 100 μL plasma from each individual). Mass spectrometry was performed in the Proteomics Core at Augusta University. The peptide‐spectrum match (PSM) count for each identified protein in the LC‐MS/MS search results was used as a semi‐quantitative measure for protein expression level. The PSM count for each protein in a specific sample was first normalized using the sum of the PSM counts for all proteins in that sample. Then, the mean PSM count for the three replicates in each group was calculated for each protein and further used for statistical analysis.

### 2.11 Immunogold labeling transmission electron microscope (TEM)

Pellets from 100 μL plasma specimens of COVID‐19 patients (*n* = 3) were collected and immunogold labeling TEM was conducted in the Electron Microscopy & Histology Core Laboratory at Augusta University as previously described.^17^ Antibody against Histone H3 (ab10799, Abcam, Cambridge, MA, USA) was used for staining. Mouse IgG (I‐2000‐1, Vector Laboratories, Newark, CA, USA) was used as a negative control.

### 2.12 RNA preparation, reverse transcription, and RT‐qPCR

Total RNA was isolated using RNeasy Plus Kits (74136, Qiagen, Germantown, MD, USA) following the manufacturer's instructions. cDNA was synthesized using the high‐capacity cDNA reverse transcription kit according to the manufacturer's instructions (4368814, Applied Biosystems, West Columbia, SC, USA). Primers were purchased from Integrated DNA Technologies (Coralville, IA, USA) and the sequences are listed in Table S2. PowerUP Green Master Mix (A25742, Applied Biosystems) was used for RT‐qPCR. TBP was used as an endogenous control. RT‐qPCR was performed and analyzed using QuantStudio 3 Real‐Time PCR system (Applied Biosystems).

### 2.13 Western blot analysis and ELISA

Pellets were collected from 100 μL pooled plasma specimens of normal individuals, ARDS, and COVID‐19 patients (*n* = 5 per group, 20 μL plasma from each individual). Western blot experiments were performed as previously described.^17^ Briefly, the pellets were lysed with radio‐immunoprecipitation assay buffer and resolved on SDS‐PAGE gels. The Histone antibodies for Western blot were purchased from Cell Signaling Technology (Danvers, MA, USA). Separated proteins were transferred onto the PVDF membrane and incubated with primary antibodies against Histone H2A (Cat#12349, Lot# 3, Cell Signaling Technology), H2B (Cat#12364T, Lot# 3, Cell Signaling Technology), H3 (Cat#4499T, Lot# 9, Cell Signaling Technology), or H4 (Cat#13919T, Lot# 3, Cell Signaling Technology). The membranes were then washed and incubated with HRP‐conjugated secondary antibody (HAF008, R&D Systems, Minneapolis, MN). Images were captured using the Chemidoc image system (Bio‐Rad Laboratories, Hercules, CA). Precision Plus Protein Standards were purchased from Bio‐Rad (Catalog# 1610375). The circulating nucleosomes and free histone H3 were measured in 100 μL plasma from normal individuals (*n* = 20) and COVID‐19 patients (*n* = 60) using Cell Death Detection ELISA Plus Kit (11774425001, MilliporeSigma) and Human Histone H3 ELISA Kit (LS‐F71552, LifeSpan BioSciences, Inc., Seattle, WA, USA) following manufacturer's instruction, respectively. To quantify the circulating nucleosome, BEAS‐2B cell‐derived nucleosomes with known concentrations were used to produce a standard curve. The concentration of IL‐1β, TNF‐α, and CXCL2 in BAL was quantified using Mouse IL‐1β DuoSet ELISA kit (DY401, R&D Systems), Mouse TNF‐α DuoSet ELISA kit (DY410, R&D Systems), and Mouse CXCL2 DuoSet ELISA kit (DY452, R&D Systems) as previously described.^21^

### 2.14 Statistics and reproducibility

Data were analyzed with SigmaPlot (Systat Software, San Jose, California, USA) and graphs were plotted using GraphPad Prism version 9.4.1 (GraphPad Software, San Diego, California, USA). Differences between the two groups were analyzed using the two‐tailed unpaired Student's *t*‐test for variables with a normal distribution or the Mann–Whitney *U* test for variables without a normal distribution. Differences between three or more groups were analyzed using a one‐way analysis of variance (ANOVA) with a Tukey's honest significance test. Parametric data are presented as mean with SD. Nonparametric data are presented as boxplots showing medians and the 25th and 75th percentiles and whiskers showing the 10th and 90th percentiles. *p*‐value of ≤.05 was considered statistically significant.

## 3 RESULTS

### 3.1 Presence of extracellular nucleosomes in the plasma of ALI/ARDS patients

**FIGURE 1:**
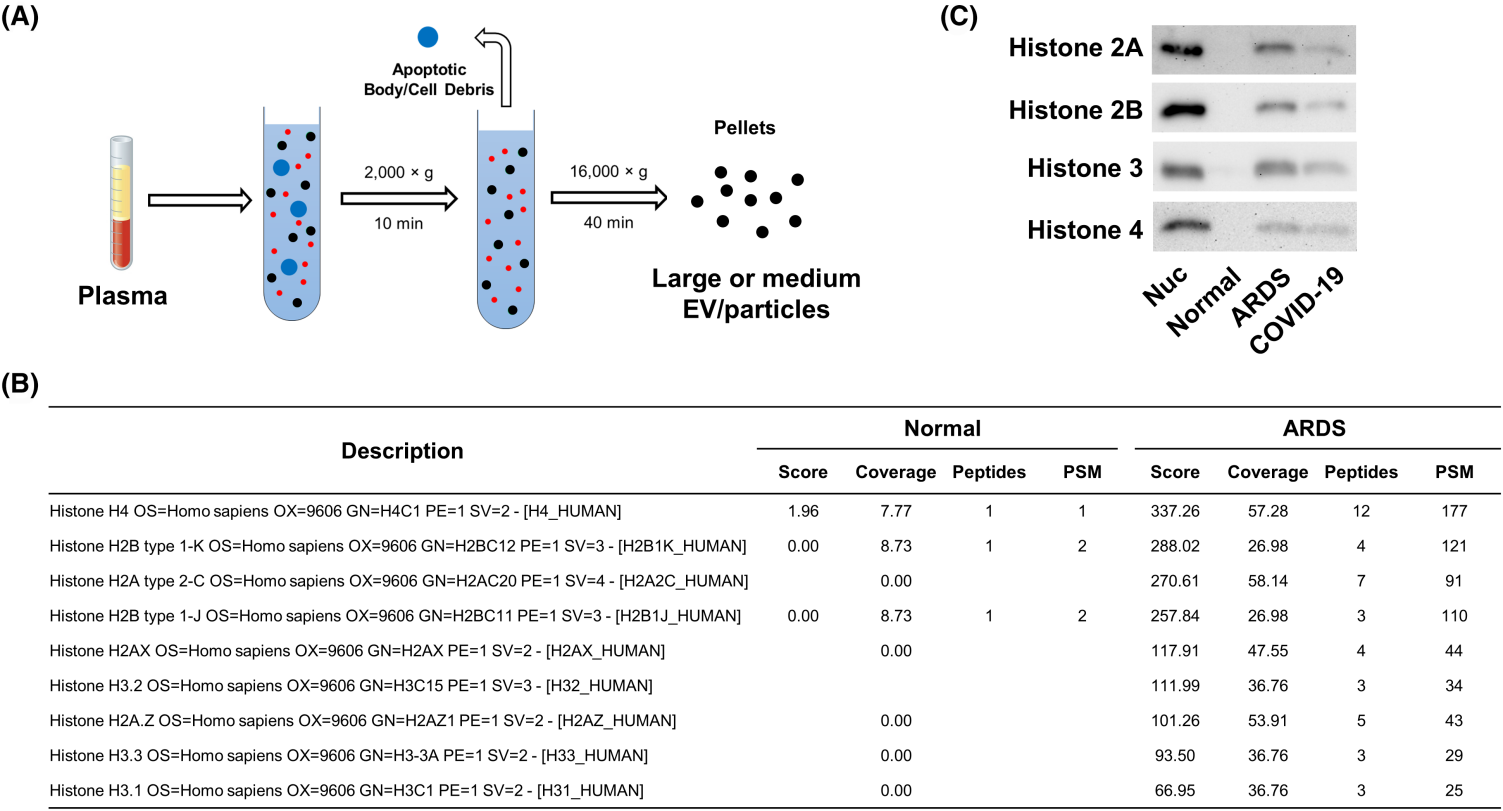
The presence of extracellular nucleosomes in the plasma of ALI/ARDS patients. (A) Schema showing the experimental procedure of obtaining pellets from plasma specimens. (B) LC‐MS/MS data showing histone proteins in the pellets from 400 μL pooled plasma of normal and ARDS groups (*n* = 4 per group). (C) Detection of histones H2A, H2B, H3, and H4 in the pellets from 500 μL pooled plasma of normal, ARDS, and COVID‐19 groups (*n* = 5 per group) using Western blotting. Nucleosomes (Nuc) of 1.5 μg were used as a positive control.

We began our investigation by comparing the differential protein expression profile in circulating extracellular vesicles (EVs) and particles from ARDS patients versus normal individuals. The pellets containing large/medium EVs and extracellular particles were collected using sequential centrifugation as described in Figure 1A. Data from LC‐MS/MS analysis revealed a significant increase in the histone proteins within the pellets (Figure 1B and Table S1). Of note, the total PSM values were very similar in these two groups (13439 in the normal group and 14977 ARDS group), suggesting the differences in the histone proteins are caused by the pathological condition not due to systematic bias, such as loading amount differences. This finding was validated in both ARDS and COVID‐19 patients using Western blot analysis (Figure 1C). Previously, Jeppesen and colleagues reported that extracellular histones belong to nonvesicular components and their release is through an amphisome‐dependent mechanism.^22^ Therefore, we hypothesized that these extracellular histones in the pellets are DNA‐binding histones existing in the form of nucleosomes.

### 3.2 Increased circulating nucleosomes in COVID‐19 patients

**FIGURE 2:**
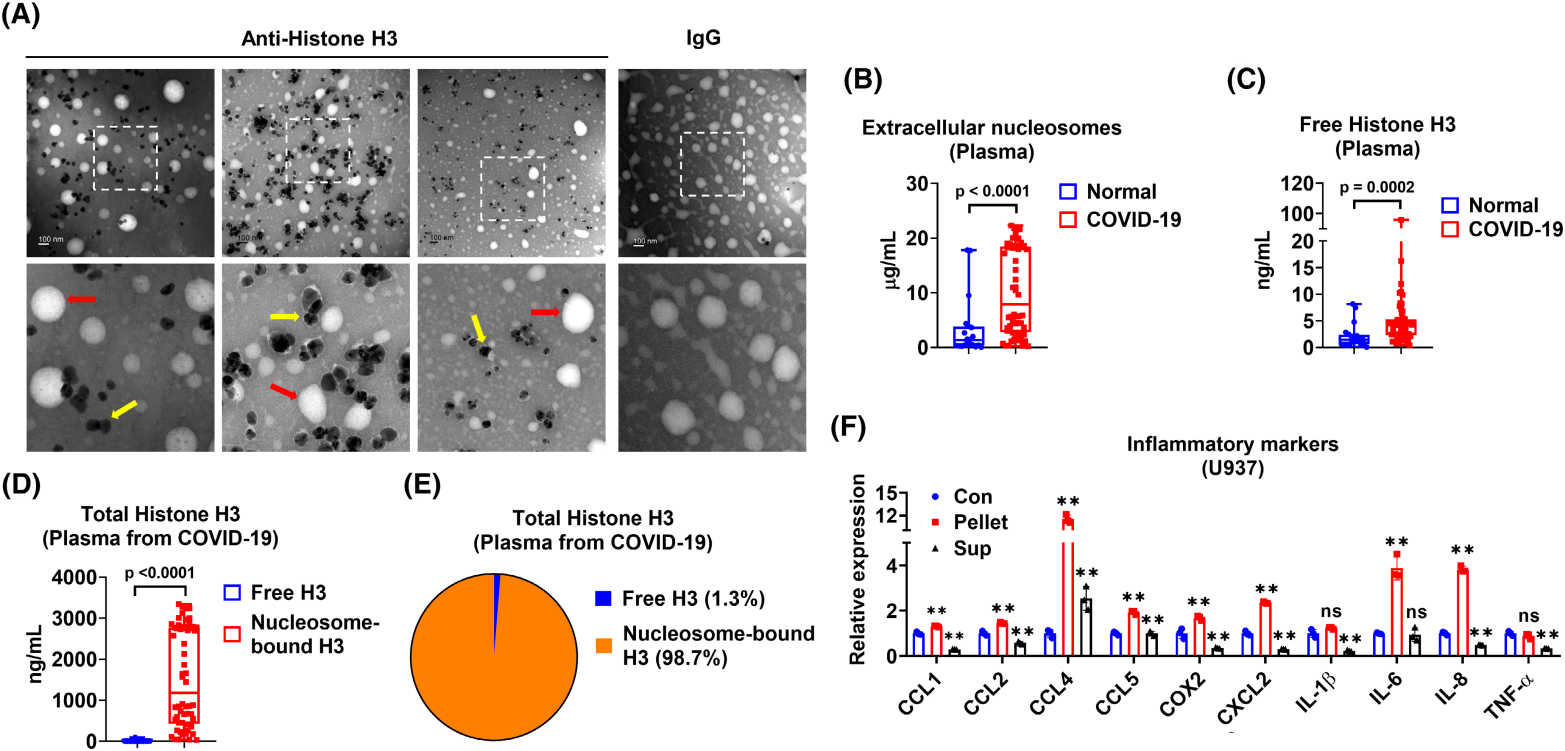
Increased circulating nucleosomes in COVID‐19 patients. (A) Pellets were collected individually from 100 μL plasma of COVID‐19 patients (*n* = 3). Immunogold labeling TEM was performed using an antibody against histone H3. Mouse IgG was used as a negative control. The red arrows indicate EVs, and the yellow arrows indicate nucleosomes. (B, C) The concentration of nucleosomes (B) and free histone H3 (C) in plasma specimens from normal individuals (*n* = 20) and COVID‐19 patients (*n* = 60) were detected using ELISA. (D, E) Comparison of concentrations (D) or percentages (E) of free histone H3 and that of nucleosome‐bound histone H3) in plasma specimens from COVID‐19 patients. (F) U937 macrophages (*n* = 3 per group) cultured in 2 mL RPMI media were treated with pellets or supernatant (Sup) separated from 100 μL pooled plasma of COVID‐19 patients (*n* = 3) for 24 h. Relative mRNA levels of inflammatory markers were detected using RT‐qPCR. In panels B–D, the boxes in the boxplots with all points show the medians with the 25th and 75th percentiles, and the whiskers show the 10th and 90th percentiles. In panel F, results are presented as mean ± SD of 3 independent experiments. ns, *p* > .05; **p < .01.

We validated the existence of nucleosomes in the extracellular particles using an independent cohort of samples from COVID‐19 patients. Pellets from the individual plasma specimens were examined using immunogold labeling TEM. The nucleosomes were indicated by a yellow arrow and the EVs were indicated by a red arrow (Figure 2A). Then, we quantified the plasma nucleosomes and free histone H3. COVID‐19 patients had significantly higher plasma nucleosomes when compared to normal subjects (Figure 2B; normal vs. COVID‐19, 3.433 μg/mL vs. 10.30 μg/mL, *p* < .0001). Similarly, the COVID‐19 group also had significantly higher free histone H3 in the circulation when compared to the normal group (Figure 2C; normal vs. COVID‐19, 2.128 ng/mL vs. 6.326 ng/mL, *p* = .0002). Then, we calculated the amount of nucleosome‐bound histone H3 as described in the Materials and Methods section based on nucleosome structure.^23^ We found that the majority of circulating Histone H3 was in the form of nucleosome rather than free form (Figure 2D,E). Functionally, we observed that the separated pellets from the plasma of COVID‐19 patients had a pro‐inflammatory effect (Figure 2F). In contrast, the supernatant reduced the expression of inflammatory markers (Figure 2F), which might be due to steroid treatment in COVID‐19 patients.^24^

### 3.3 Nucleosomes induce macrophage activation

**FIGURE 3:**
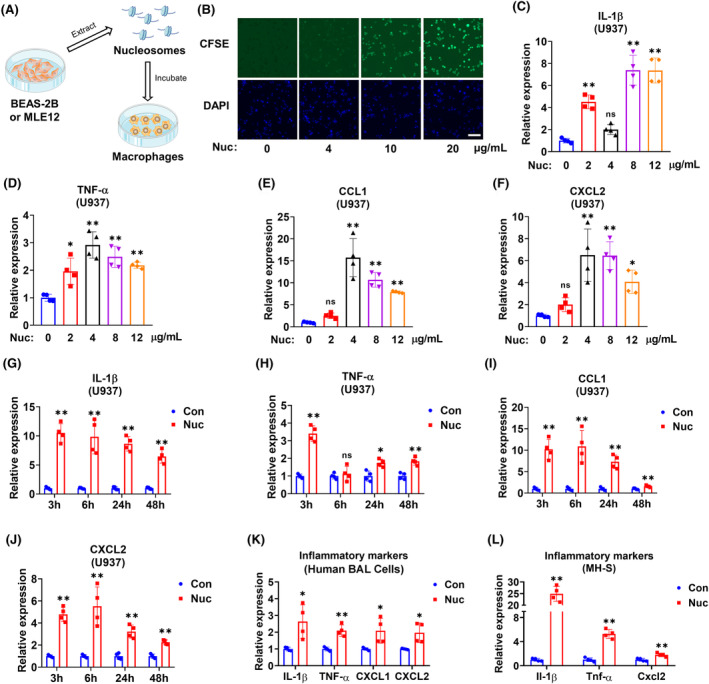
Nucleosomes induce macrophage activation. (A) Schematic illustration of the design of in vitro experiments. (B) U937 macrophages (*n* = 3 per group) were incubated with indicated concentrations of CFSE‐labeled nucleosomes (Nuc) for 1 h. DAPI was used to stain the cell nuclei. The images were taken using a fluorescence microscope. Scale bar = 100 μm. (C–F) U937 macrophages (*n* = 4 per group) were incubated with indicated concentrations of nucleosomes for 24 h. Relative mRNA levels of IL‐1β (C), TNF‐α (D), CCL1 (E), and CXCL2 (F) were detected using RT‐qPCR. (G–J) U937 macrophages (*n* = 4 per group) were treated with 4 μg/mL nucleosomes for the period as indicated. Relative mRNA levels of IL‐1β (G), TNF‐α (H), CCL1 (I), and CXCL2 (J) were detected using RT‐qPCR. (K, L) Primary human BAL cells (*n* = 4 per group) (K) or MH‐S macrophages (*n* = 4 per group) (L) were treated with 4 μg/mL nucleosomes for 6 h. Relative mRNA levels of IL‐1β, TNF‐α, CXCL1, and CXCL2 were detected using RT‐qPCR. Data are presented as mean ± SD of four independent experiments. ns, *p* > .05; *p < .05; **p < .01.

We extracted nucleosomes from normal lung epithelial cells and evaluated their role in macrophages (Figure 3A). First, we observed the interaction between CFSE‐labeled nucleosomes and cultured macrophages (Figure 3B). Functionally, nucleosomes can induce macrophage activation in both dose‐dependent (Figure 3C–F) and time‐dependent manners (Figure 3G–J). A similar pro‐inflammatory effect was observed in primary human BAL cells and murine MH‐S cells (Figure 3K,L).

### 3.4 Nucleosomes mediate macrophage–epithelial cell interaction

**FIGURE 4:**
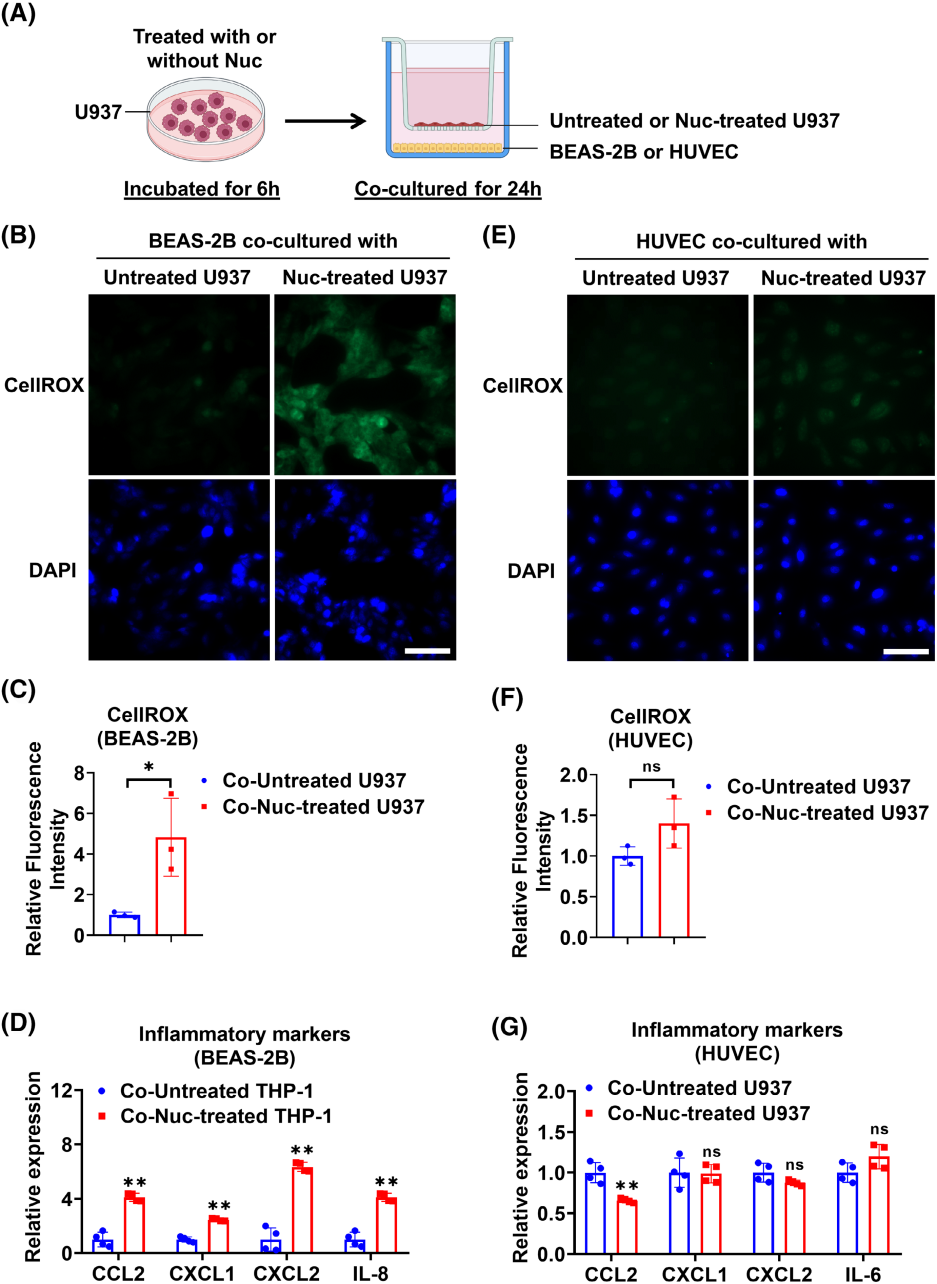
Nucleosomes mediate macrophage–epithelial cell interaction. (A) Schematic illustration of the BEAS‐2B or HUVEC co‐cultured with untreated or 4 μg/mL nucleosome (Nuc)‐treated U937. (B–D) BEAS‐2B and U937 were co‐cultured for 24 h. The cellular ROS levels in BEAS‐2B cells (*n* = 3 per group) were stained using CellROX (B) and the relative fluorescence intensity was quantified (C). DAPI was used to stain the cell nuclei. The images were taken using a fluorescence microscope. Scale bar = 100 μm. Relative mRNA levels of CCL2, CXCL1, CXCL2, and IL‐8 in BEAS‐2B cells (*n* = 4 per group) were detected using RT‐qPCR (D). (E–G) HUVEC and U937 were co‐cultured for 24 h. The cellular ROS levels in HUVEC cells (*n* = 3 per group) were stained (E) and quantified (F). DAPI was used to stain the cell nuclei. The images were taken using a fluorescence microscope. Scale bar = 100 μm. Relative mRNA levels of CCL2, CXCL1, CXCL2, and IL‐6 in HUVEC cells (*n* = 4 per group) were detected using RT‐qPCR (G). Data are presented as mean ± SD of 3–4 independent experiments. ns, *p* > .05; *p < .05; **p < .01.

To understand the effect of nucleosome‐activated macrophages on other compartmental lung cells, we conducted in vitro experiments using a co‐culture model as shown in Figure 4A. When co‐cultured with nucleosome‐activated U937, BEAS‐2B epithelial cells showed increased ROS generation (Figure 4B,C) and upregulated cytokine and chemokine expression (Figure 4D). In contrast, nucleosome‐activated U937 did not significantly change the ROS production and pro‐inflammatory gene expression in HUVEC (Figure 4E–G). Together, these results provide evidence for the cross‐talk between nucleosome‐activated macrophages and lung epithelial cells.

### 3.5 Intratracheal administration of nucleosomes causes lung inflammation

**FIGURE 5:**
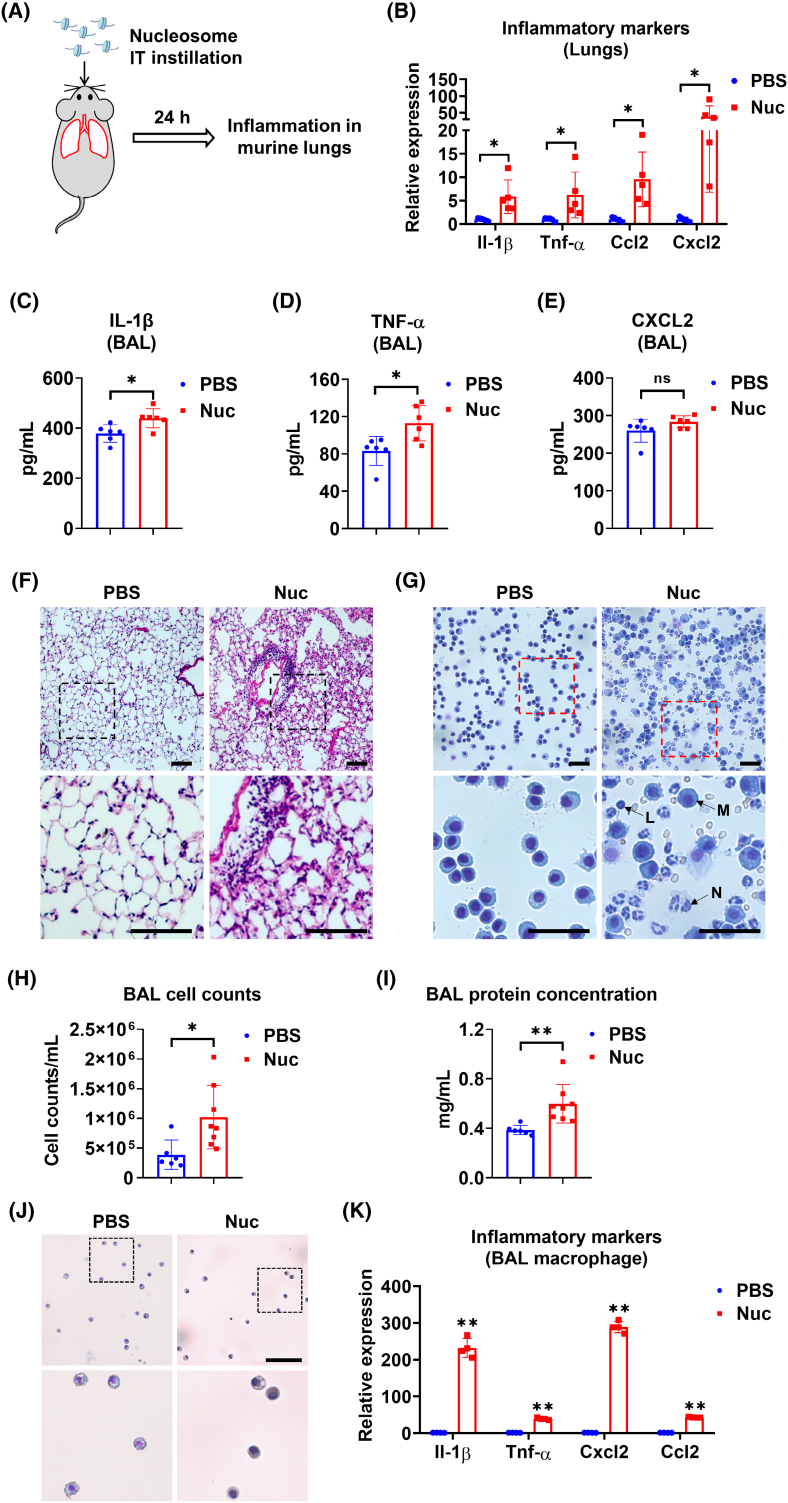
Intratracheal administration of nucleosomes induces lung inflammation. (A) Schematic illustration of IT administration of nucleosomes. (B–K) Mice (*n* = 4–8 per group) received 50 μL PBS with or without 50 μg nucleosome (Nuc) via IT. Mice were euthanized 24 h after treatment. Relative mRNA levels of Il‐1β, Tnf‐α, CCL2, and Cxcl2 were detected in harvested lungs (*n* = 5 per group) using RT‐qPCR (B). BAL (*n* = 6 per group) IL‐1β (C), TNF‐α (D), and CXCL2 (E) concentrations were measured using ELISA. H&E staining (*n* = 4 per group) was performed on lung sections (F) and BAL cells (G). Scale bar = 100 μm and 50 μm, respectively. L, Lymphocytes; M, Macrophage; N, Neutrophil. Total numbers of leukocytes (*n* = 6 for PBS group and *n* = 8 for Nuc group) were counted in the BAL (H). The BAL protein concentration (*n* = 6 for PBS group and *n* = 8 for Nuc group) was measured (I). BAL macrophages (*n* = 4 per group) were enriched (J) and relative mRNA levels of Il‐1β, Tnf‐α, Cxcl2, and Ccl2 were detected using RT‐qPCR (K). Scale bar = 100 μm. Data are mean ± SD. ns, *p* > .05; *p < .05, **p < .01.

Lung inflammation was measured after nucleosome administration via IT (Figure 5A). Our data showed the expression of Il‐1β, Tnf‐α, Ccl2, and Cxcl2 in lung tissues was significantly induced by nucleosomes (Figure 5B). The secretion of IL‐1β, and TNF‐α but not CXCL2 in BAL was also increased (Figure 5C–E). The increased lung inflammation in nucleosome‐treated mice was also reflected by H&E staining in the lung section and BAL cells (Figure 5F,G), as well as a significant increase in the BAL cell counts and protein concentration (Figure 5H,I). Meanwhile, we enriched BAL macrophages (Figure 5J) and found highly increased pro‐inflammatory markers in BAL macrophages from nucleosome‐treated mice (Figure 5K).

### 3.6 Intravenous administration of nucleosomes causes inflammation in various organs

**FIGURE 6:**
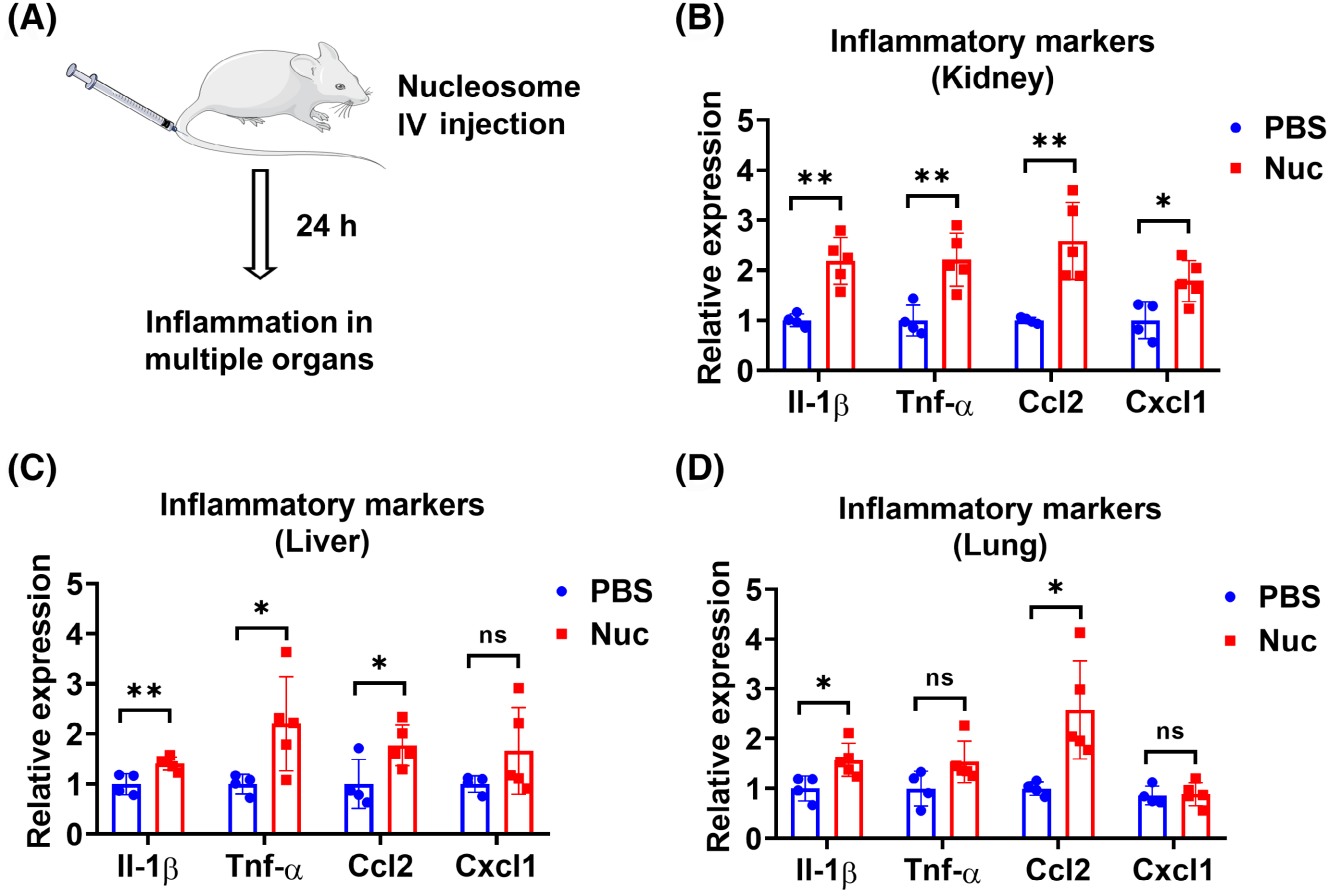
Intravenous administration of nucleosomes causes inflammation in various organs. (A) A schematic illustration of IV administration of nucleosomes. Mice (*n* = 4–5 per group) received 300 μL PBS with or without 300 μg nucleosomes (Nuc) via IV. Mice were euthanized 24 h after treatment. (B–D) Relative mRNA levels (*n* = 4 for PBS group and *n* = 5 for Nuc group) of Il‐1β, Tnf‐α, Ccl2, and Cxcl1 in kidneys (B), livers (C), and lungs (D) using RT‐qPCR. Results represent mean ± SD. ns, *p* > .05; *p < .05, **p < .01.

We further tested the inflammatory effect of nucleosomes after intravenous injection (IV). (Figure 6A). Our data showed that nucleosomes upregulated the pro‐inflammatory markers in kidneys, livers, and lungs (Figure 6B–D), implying that circulating nucleosomes may elicit systemic inflammation, affecting multiple organs beyond the site of administration.

### 3.7 Nucleosomes cause inflammation through the activation of NF‐κB signaling

**FIGURE 7:**
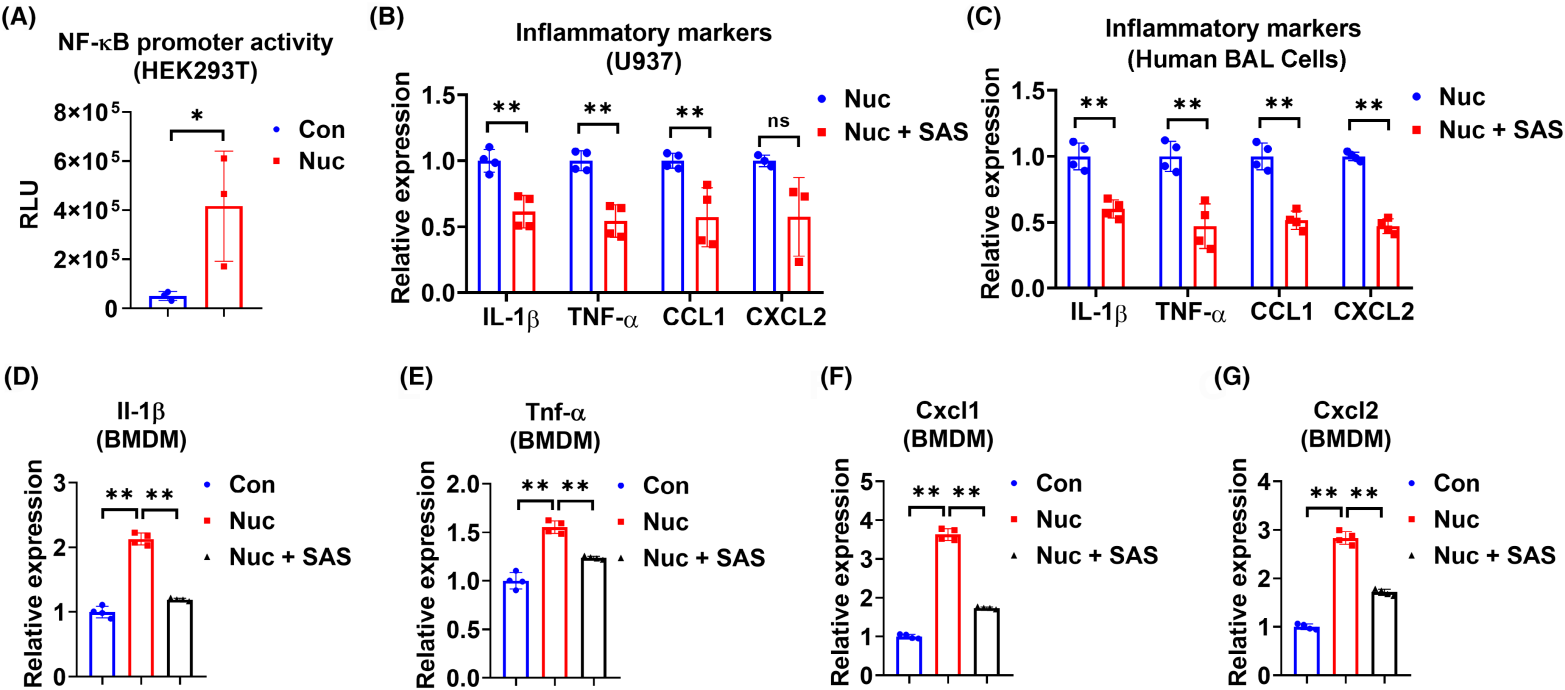
Nucleosomes activate NF‐κB signaling in macrophages. (A) NF‐κB luciferase reporter plasmid was transfected into HEK293T cells and then cells were treated with or without 4 μg/mL nucleosomes (Nuc). Forty‐eight hours after transfection, a luciferase assay (*n* = 3 per group) was performed to determine the NF‐κB promoter activity. (B, C) Cells were preincubated with 20 μM sulfasalazine for 4 h and then treated with 4 μg/mL nucleosomes for an additional 24 h. Relative mRNA levels of Il‐1β, Tnf‐α, Ccl1, and Cxcl2 were detected in U937 macrophages (*n* = 4 per group) (B) and primary human BAL cells (*n* = 4 per group) (C) using RT‐qPCR. (D–F) BMDM (*n* = 4 per group) were preincubated with 20 μM sulfasalazine for 4 h and then treated with 4 μg/mL nucleosomes for an additional 24 h. Relative mRNA levels of Il‐1β (D), Tnf‐α (E), Cxcl1 (F), and Cxcl2 (G) were detected using RT‐qPCR. Data are presented as mean ± SD of 3–4 independent experiments. ns, *p* > .05; *p < .05; **p < .01.

Using luciferase reporter assay, we found that nucleosomes significantly enhanced NF‐κB promoter activity (Figure 7A). We preincubated cells with sulfasalazine, which is a known inhibitor of NF‐κB activation.^25^ Our data showed that sulfasalazine significantly attenuated the nucleosome‐induced macrophage activation (Figure 7B–G).

## 4 DISCUSSION

In this study, we found that increased circulating nucleosomes as the leading form of extracellular histones in COVID‐19 patients. Functionally, nucleosomes activated macrophages and induced inflammation in different organs. Mechanistically, we observed nucleosomes activating the NF‐κB signaling, while inhibition of NF‐κB by sulfasalazine attenuated nucleosome‐induced macrophage activation.

Recent studies have highlighted the potential involvement of nucleosomes in lung inflammation and ARDS.^6^, ^8^ However, a robust mechanistic link between the circulating nucleosomes and lung inflammation contributing to ALI is missing. Our analysis of plasma specimens from ARDS and COVID‐19 cohorts, following centrifugation at specific speeds tailored to precipitate cell‐released structures, revealed pellets containing mixtures of extracellular particles and EVs, implicating these components in the disease pathogenesis. Evidently, the substantial presence of histone proteins primarily represented histones bound to various structures, particularly nucleosomes, emphasizing the prevalence of extracellular, circulating nucleosomes in both ARDS and COVID‐19 patients.

The amplified presence of nucleosomes in the plasma samples collected from ALI patients prompted us to further explore how cells, particularly macrophages, respond immunologically to nucleosome exposure. Given their pivotal role as the first‐line defense innate immune cells,^26^ macrophages were selected for this study due to their involvement in recognizing immunogenic particles, regulating immunogenic reactions and adaptive immunity, and engulfing and degrading particulates.^26^, ^27^ One strength of our study lies in the use of species‐matched, lung epithelial cell‐derived nucleosomes. Previous basic studies predominantly relied on *Escherichia coli*‐produced recombinant histones or calf thymus histones.^4^, ^7^, ^8^, ^9^ Owing to the origins and characteristics of these histone proteins, it is plausible that they may harbor endotoxin contaminants. Even minute quantities of residual endotoxins possess the potential to elicit significant inflammation by activating CD1c + dendritic cells.^28^ This contamination can significantly confound the outcomes of experiments and lead to exaggerated results and outcomes. In contrast, one strength of our study is that we adopted a distinct approach wherein nucleosomes are extracted from untreated (naive) epithelial cells, circumventing the potential confounding effects of endotoxin contamination.

There exists a gap in our understanding of the immunological significance of the interaction between macrophages and nucleosomes. Previous research has highlighted the role of nucleosomes in modulating immune responses and inflammation. Xu et al. demonstrated that extracellular nucleosomes released during cell death can serve as a potent trigger, activating innate immune pathways and promoting inflammation in various disease contexts, including ALI and sepsis.^7^ By administering nucleosomes directly into the lung via the IT route, we mimicked the local release of nucleosomes within the pulmonary microenvironment, which is particularly relevant in the context of lung injury and respiratory diseases. The pronounced upregulation of pro‐inflammatory markers at both gene and protein levels we observed following nucleosome administration aligns with other reports from preclinical models of ALI, where extracellular nucleosomes have been shown to induce cytokine production, recruit immune cells, and exacerbate tissue damage.^29^, ^30^ Moreover, our investigation into the systemic effects of nucleosomes following IV administration yielded compelling insights into their ability to induce inflammation across multiple organs. A similar effect has been documented in a few other studies, where circulating nucleosomes were implicated in systemic inflammatory responses and organ dysfunction, particularly in the context of sepsis and systemic inflammatory diseases.^4^, ^5^, ^31^, ^32^

Extracellular histones are released into the extracellular space in two major forms: free histones and DNA‐bound histones (nucleosomes).^4^, ^8^ The lack of a distinction between free histones and nucleosomes leads to ambiguity in extracellular histone studies.^4^, ^8^ Indeed, in some instances, these terms were used interchangeably, further complicating the interpretation of experimental findings. Even though current techniques can distinguish these two forms and quantify nucleosomes, no studies have reported their actual concentrations in the blood under normal or disease conditions. For the first time, our study quantified the concentrations of circulating nucleosomes from COVID‐19 patients and revealed nucleosomes as the predominant form of extracellular histones.

The landscape of research on the inflammatory properties of nucleosomes has been significantly influenced by a seminal study conducted by Xu et al., wherein the group administered 50–100 mg/kg of nucleosomes to mice.^7^ Considering that an average mouse typically possesses approximately 80 μL/g of blood volume, the administered doses of nucleosomes translate to concentrations ranging from 625 to 1250 μg/mL. In contrast, our analysis of plasma samples from severely infected COVID‐19 patients revealed that even amidst severe inflammation, the mean plasma concentration of nucleosomes was approximately 8 μg/mL. Thus, the dosages utilized by Xu et al. were notably 150–300 times higher than those observed in severe COVID‐19 patients. These exceedingly high doses lack a clear rationale and fail to align with pathophysiological or clinical evidence. Although such markedly supraphysiological doses are undoubtedly capable of eliciting inflammation, they do not accurately mirror the physiological conditions observed in clinical settings. In contrast, our experimental approach incorporated doses that more closely resemble the concentrations observed during acute inflammation, both in vitro and in vivo. By adopting doses that better mimic real‐world scenarios, our study offers a more clinically relevant perspective on the inflammatory effects of nucleosomes, providing valuable insights into the pathophysiology of inflammatory conditions.

This study has several limitations. First of all, the sample size of the COVID‐19 cohort in this study was small. We did not observe significant correlations between nucleosome levels and clinical outcomes, such as mortality or the partial pressure of oxygen/fraction of inspired oxygen ratio (PaO_2_/FiO_2_). Thus, we were unable to conclude if circulating nucleosomes can reflect the severity and prognosis of COVID‐19. Second, we reported the increased circulating nucleosomes in patients with ALI/ARDS. Nevertheless, the kinetics of circulating nucleosomes in the patients were still unknown. Third, pooled plasma samples were used in several experiments for screening purposes. Thus, statistical analysis was not conducted when using the pooled samples. Further studies are required to quantify the data using individual samples. Fourth, when calculating nucleosome concentrations, we only accounted for the weight of histone proteins, omitting consideration of the weight of the short DNA sequence. The variable number of nucleotides and the unique DNA sequence associated with each nucleosome introduce potential variability that were not fully addressed in our analysis. Fifth, the detailed molecular mechanism by which nucleosomes activated the macrophages was not fully addressed. For instance, the specific receptor(s) responsible for nucleosome binding and cellular entry remain unidentified in our study. We have yet to determine the specific histone protein modifications or DNA sequences that trigger the transition of cellular nucleosomes to pathogenic extracellular (circulating) nucleosomes. Understanding these molecular mechanisms could provide crucial insights into disease pathogenesis. Due to the essential role of histone proteins in the survival of all species, we were unable to conduct in vivo experiments using histone knockout mice, which could have provided further mechanistic understanding. Finally, we have not pinpointed the predominant cell types responsible for nucleosome release, such as epithelial cells or neutrophils. Clarifying the cellular sources of circulating nucleosomes would enhance our understanding of their origins and potential implications in disease processes. Acknowledging and addressing these limitations will be integral to advancing our understanding of nucleosome biology and its relevance in disease contexts.

Collectively, our studies revealed that nucleosomes are the major form of released histones in circulation and identified their function in macrophage activation and lung inflammation in the pathogenesis of ALI.

## AUTHOR CONTRIBUTIONS

D.Z. and S.D. designed the research. SD performed experiments. S.D., D.Z., Y.Z., S.A., P.R.S., S.I., W.Z., G.R., N.R., M.J.L.‐Z., V.M.‐G., L.J.‐A., A.C.‐L., T.S.R.‐R., J.Z., and X.W. collected, analyzed, and interpreted data. S.D., P.R.S., S.I., and D.Z. wrote the manuscript.

## DISCLOSURES

The authors have declared that no conflict of interest exists.

## Supporting information


**Table S1.**.

## Data Availability

The data that support the findings of this study are available in the Materials and Methods, Results, and/or Supplemental Material of this article.

## References

[1] He YQ , Zhou CC , Yu LY , et al. Natural product derived phytochemicals in managing acute lung injury by multiple mechanisms. Pharmacol Res. 2021;163:105224. 33007416 10.1016/j.phrs.2020.105224 PMC7522693

[2] Jia Q , Yang Y , Chen X , Yao S , Hu Z . Emerging roles of mechanosensitive ion channels in acute lung injury/acute respiratory distress syndrome. Respir Res. 2022;23:366. 36539808 10.1186/s12931-022-02303-3 PMC9764320

[3] Liu X , Ling X , He J , et al. Piezo1‐targeted aerosol inhalation nanoparticles for acute lung injury. J Mater Sci Technol. 2023;141:21‐31.

[4] Silk E , Zhao H , Weng H , Ma D . The role of extracellular histone in organ injury. Cell Death Dis. 2017;8:e2812. 28542146 10.1038/cddis.2017.52 PMC5520745

[5] Chen Q , Ye L , Jin Y , et al. Circulating nucleosomes as a predictor of sepsis and organ dysfunction in critically ill patients. Int J Infect Dis. 2012;16:e558‐e564. 22609014 10.1016/j.ijid.2012.03.007

[6] Yehya N , Fazelinia H , Lawrence GG , et al. Plasma nucleosomes are associated with mortality in pediatric acute respiratory distress syndrome. Crit Care Med. 2021;49:1149‐1158. 33729723 10.1097/CCM.0000000000004923 PMC8217097

[7] Xu J , Zhang X , Pelayo R , et al. Extracellular histones are major mediators of death in sepsis. Nat Med. 2009;15:1318‐1321. 19855397 10.1038/nm.2053 PMC2783754

[8] Marsman G , Zeerleder S , Luken BM . Extracellular histones, cell‐free DNA, or nucleosomes: differences in immunostimulation. Cell Death Dis. 2016;7:e2518. 27929534 10.1038/cddis.2016.410 PMC5261016

[9] Bosmann M , Grailer JJ , Ruemmler R , et al. Extracellular histones are essential effectors of C5aR‐ and C5L2‐mediated tissue damage and inflammation in acute lung injury. FASEB J. 2013;27:5010‐5021. 23982144 10.1096/fj.13-236380 PMC3834784

[10] Wang H , Zang C , Ren M , et al. Cellular uptake of extracellular nucleosomes induces innate immune responses by binding and activating cGMP‐AMP synthase (cGAS). Sci Rep. 2020;10:15385. 32958884 10.1038/s41598-020-72393-w PMC7505961

[11] McAnena P , Brown JA , Kerin MJ . Circulating nucleosomes and nucleosome modifications as biomarkers in cancer. Cancers. 2017;9:5. 28075351 10.3390/cancers9010005 PMC5295776

[12] Shabrish S , Mittra I . Cytokine storm as a cellular response to dsDNA breaks: a new proposal. Front Immunol. 2021;12:622738. 33597956 10.3389/fimmu.2021.622738 PMC7882731

[13] Dolan C , Miller T , Jill J , et al. Characterizing circulating nucleosomes in the plasma of dogs with lymphoma. BMC Vet Res. 2021;17:276. 34399763 10.1186/s12917-021-02991-x PMC8365961

[14] Bauden M , Pamart D , Ansari D , et al. Circulating nucleosomes as epigenetic biomarkers in pancreatic cancer. Clin Epigenetics. 2015;7:106. 26451166 10.1186/s13148-015-0139-4 PMC4597435

[15] Almuntashiri S , Han Y , Youngblood HA , et al. Identification of circulating microvesicle‐encapsulated miR‐223 as a potential novel biomarker for ARDS. Physiol Rep. 2022;10:e15494. 36353917 10.14814/phy2.15494 PMC9647359

[16] Zhang D , Lee H , Wang X , et al. A potential role of microvesicle‐containing miR‐223/142 in lung inflammation. Thorax. 2019;74:865‐874. 31331947 10.1136/thoraxjnl-2018-212994 PMC7036165

[17] Han Y , Zhu Y , Almuntashiri S , et al. Extracellular vesicle‐encapsulated CC16 as novel nanotherapeutics for treatment of acute lung injury. Mol Ther. 2023;31:1346‐1364. 36635966 10.1016/j.ymthe.2023.01.009 PMC10188639

[18] Salahuddin S , Thomson E , Meziane O , et al. Processing of bronchoalveolar lavage fluid and matched blood for alveolar macrophage and CD4+ T‐cell Immunophenotyping and HIV reservoir assessment. J Vis Exp. 2019;148:e59427. 10.3791/59427 31282892

[19] Zhang X , Goncalves R , Mosser DM . The isolation and characterization of murine macrophages. Curr Protoc Immunol. 2008;14:14 11 11‐14 11 14. 10.1002/0471142735.im1401s83 PMC2834554 19016445

[20] Zhu Y , Han Y , Almuntashiri S , et al. Dysregulation of miR‐103a mediates cigarette smoking‐induced lipid‐laden macrophage formation. Am J Respir Cell Mol Biol. 2022;67:695‐707. 36066909 10.1165/rcmb.2022-0202OC PMC9743184

[21] Han Y , Zhu Y , Youngblood HA , et al. Nebulization of extracellular vesicles: a promising small RNA delivery approach for lung diseases. J Control Release. 2022;352:556‐569. 36341934 10.1016/j.jconrel.2022.10.052 PMC13092262

[22] Jeppesen DK , Fenix AM , Franklin JL , et al. Reassessment of exosome composition. Cell. 2019;177:428‐445.e418. 30951670 10.1016/j.cell.2019.02.029 PMC6664447

[23] McGinty RK , Tan S . Nucleosome structure and function. Chem Rev. 2015;115:2255‐2273. 25495456 10.1021/cr500373h PMC4378457

[24] RECOVERY Collaborative Group , Horby P , Lim WS , et al. Dexamethasone in hospitalized patients with Covid‐19. N Engl J Med. 2021;384:693‐704. 32678530 10.1056/NEJMoa2021436 PMC7383595

[25] Weber CK , Liptay S , Wirth T , Adler G , Schmid RM . Suppression of NF‐kappaB activity by sulfasalazine is mediated by direct inhibition of IkappaB kinases alpha and beta. Gastroenterology. 2000;119:1209‐1218. 11054378 10.1053/gast.2000.19458

[26] Vijay K . Chapter 1: Macrophages: the potent immunoregulatory innate immune cells. In: Khalid Hussain B , ed. Macrophage Activation. IntechOpen; 2019.

[27] Chen S , Saeed A , Liu Q , et al. Macrophages in immunoregulation and therapeutics. Signal Transduct Target Ther. 2023;8:207. 37211559 10.1038/s41392-023-01452-1 PMC10200802

[28] Schwarz H , Schmittner M , Duschl A , Horejs‐Hoeck J . Residual endotoxin contaminations in recombinant proteins are sufficient to activate human CD1c+ dendritic cells. PLoS ONE. 2014;9:e113840. 25478795 10.1371/journal.pone.0113840 PMC4257590

[29] Saffarzadeh M , Juenemann C , Queisser MA , et al. Neutrophil extracellular traps directly induce epithelial and endothelial cell death: a predominant role of histones. PLoS ONE. 2012;7:e32366. 22389696 10.1371/journal.pone.0032366 PMC3289648

[30] Abrams ST , Zhang N , Dart C , et al. Human CRP defends against the toxicity of circulating histones. J Immunol. 2013;191:2495‐2502. 23894199 10.4049/jimmunol.1203181

[31] Zeerleder S , Zwart B , Wuillemin WA , et al. Elevated nucleosome levels in systemic inflammation and sepsis. Crit Care Med. 2003;31:1947‐1951. 12847387 10.1097/01.CCM.0000074719.40109.95

[32] Wildhagen KC , Wiewel MA , Schultz MJ , et al. Extracellular histone H3 levels are inversely correlated with antithrombin levels and platelet counts and are associated with mortality in sepsis patients. Thromb Res. 2015;136:542‐547. 26232351 10.1016/j.thromres.2015.06.035

