# Supplementary material for: The impact of circulating nucleosomes on inflammation in acute lung injury
Source: FASEB J. 2024 Nov 29;38(23):e70214. doi: 10.1096/fj.202401571RR (PMC11606509; doi:10.1096/fj.202401571RR)
Supplement: Supplementary file 1 — Table S1.. [file FSB2-38-e70214-s001.pdf]

Supplementary Table 1 Multiconsensus from normal and ARDS groups

Accession	Description	ΣCoverage	Σ# Proteins	Σ# Unique Peptides	Σ# Peptides	Σ# PSMs	Score A2	Coverage A2	# Peptides A2	# PSM A2	Score B2	Coverage B2	# Peptides B2	# PSM B2	# AAs	MW [kDa]	calc. pI
P01834	Immunoglobulin kappa constant OS=Homo sapiens OX=9606 GN=IGKC PE=1 SV=2 - [IGKC_HUMAN]	82.24	1	4	10	498	548.35	82.24	10	159	1079.89	82.24	10	339	107	11.8	6.52
P0DOY2	Immunoglobulin lambda constant 2 OS=Homo sapiens OX=9606 GN=IGLC2 PE=1 SV=1 - [IGLC2_HUMAN]	77.36	4	3	7	248	335.47	65.09	5	109	443.07	77.36	7	139	106	11.3	7.24
P08319	All-trans-retinol dehydrogenase [NAD(+)] ADH4 OS=Homo sapiens OX=9606 GN=ADH4 PE=1 SV=5 - [ADH4_HUMAN]	77.11	1	17	18	143		0.00			448.89	77.11	18	143	380	40.2	7.94
P02679	Fibrinogen gamma chain OS=Homo sapiens OX=9606 GN=FGG PE=1 SV=3 - [FIBG_HUMAN]	74.39	1	31	31	738	1140.91	72.63	28	400	978.19	74.17	28	338	453	51.5	5.62
P02768	Albumin OS=Homo sapiens OX=9606 GN=ALB PE=1 SV=2 - [ALBU_HUMAN]	73.23	1	49	49	2723	4520.42	70.94	47	1531	3601.02	67.82	44	1192	609	69.3	6.28
P01024	Complement C3 OS=Homo sapiens OX=9606 GN=C3 PE=1 SV=2 - [CO3_HUMAN]	70.05	1	88	88	1056	2359.51	70.05	88	775	882.37	53.76	59	281	1663	187.0	6.40
P60709	Actin, cytoplasmic 1 OS=Homo sapiens OX=9606 GN=ACTB PE=1 SV=1 - [ACTB_HUMAN]	69.33	7	9	15	150	169.27	41.07	10	60	280.22	67.47	14	90	375	41.7	5.48
P02675	Fibrinogen beta chain OS=Homo sapiens OX=9606 GN=FGB PE=1 SV=2 - [FIBB_HUMAN]	68.84	1	29	29	969	1636.46	65.99	27	514	1426.64	66.19	28	455	491	55.9	8.27
P01876	Immunoglobulin heavy constant alpha 1 OS=Homo sapiens OX=9606 GN=IGHA1 PE=1 SV=2 - [IGHA1_HUMAN]	68.56	1	10	13	279	400.96	62.32	12	117	555.23	66.57	12	162	353	37.6	6.51
P0CG04	Immunoglobulin lambda constant 1 OS=Homo sapiens OX=9606 GN=IGLC1 PE=1 SV=1 - [IGLC1_HUMAN]	67.92	3	1	5	181	260.12	65.09	4	83	335.81	67.92	5	98	106	11.3	7.87
P0DJI8	Serum amyloid A-1 protein OS=Homo sapiens OX=9606 GN=SAA1 PE=1 SV=1 - [SAA1_HUMAN]	66.39	1	5	9	93	4.93	11.48	1	2	303.09	66.39	9	91	122	13.5	6.79
P01857	Immunoglobulin heavy constant gamma 1 OS=Homo sapiens OX=9606 GN=IGHG1 PE=1 SV=1 - [IGHG1_HUMAN]	65.15	2	9	18	704	744.12	62.73	15	265	1346.79	65.15	18	439	330	36.1	8.19
P02647	Apolipoprotein A-I OS=Homo sapiens OX=9606 GN=APOA1 PE=1 SV=1 - [APOA1_HUMAN]	62.92	1	24	24	302	500.91	62.92	24	178	330.41	60.67	20	124	267	30.8	5.76
P35527	Keratin, type I cytoskeletal 9 OS=Homo sapiens OX=9606 GN=KRT9 PE=1 SV=3 - [K1C9_HUMAN]	61.64	1	19	19	96	187.33	51.04	18	60	105.32	51.85	14	36	623	62.0	5.24
P04114	Apolipoprotein B-100 OS=Homo sapiens OX=9606 GN=APOB PE=1 SV=2 - [APOB_HUMAN]	60.79	1	232	232	4669	6943.86	57.94	218	2366	7012.70	58.10	217	2303	4563	515.3	7.05
P02766	Transthyretin OS=Homo sapiens OX=9606 GN=TTR PE=1 SV=1 - [TTHY_HUMAN]	59.86	1	6	6	23	62.92	55.78	5	17	25.04	35.37	3	6	147	15.9	5.76
P01591	Immunoglobulin J chain OS=Homo sapiens OX=9606 GN=JCHAIN PE=1 SV=4 - [IGJ_HUMAN]	59.75	1	7	7	47	46.69	59.75	6	19	73.88	59.75	7	28	159	18.1	5.24
P01009	Alpha-1-antitrypsin OS=Homo sapiens OX=9606 GN=SERPINA1 PE=1 SV=3 - [A1AT_HUMAN]	59.57	1	24	24	305	513.43	55.02	22	179	377.20	57.42	21	126	418	46.7	5.59
P00739	Haptoglobin-related protein OS=Homo sapiens OX=9606 GN=HPR PE=2 SV=2 - [HPTR_HUMAN]	59.48	1	6	19	179	286.35	59.20	17	112	159.63	42.24	13	67	348	39.0	7.09
P02649	Apolipoprotein E OS=Homo sapiens OX=9606 GN=APOE PE=1 SV=1 - [APOE_HUMAN]	59.31	1	18	18	289	466.21	59.31	18	154	388.70	54.89	17	135	317	36.1	5.73
Q16777	Histone H2A type 2-C OS=Homo sapiens OX=9606 GN=H2AC20 PE=1 SV=4 - [H2A2C_HUMAN]	58.14	2	5	7	91		0.00			270.61	58.14	7	91	129	14.0	10.90
P01871	Immunoglobulin heavy constant mu OS=Homo sapiens OX=9606 GN=IGHM PE=1 SV=4 - [IGHM_HUMAN]	58.06	2	19	19	739	652.35	53.64	18	239	1386.31	58.06	19	500	453	49.4	6.77
P62805	Histone H4 OS=Homo sapiens OX=9606 GN=H4C1 PE=1 SV=2 - [H4_HUMAN]	57.28	1	12	12	178	1.96	7.77	1	1	337.26	57.28	12	177	103	11.4	11.36
P02787	Serotransferrin OS=Homo sapiens OX=9606 GN=TF PE=1 SV=3 - [TRFE_HUMAN]	56.59	1	33	33	261	410.54	52.01	29	136	370.31	53.58	30	125	698	77.0	7.12
P06702	Protein S100-A9 OS=Homo sapiens OX=9606 GN=S100A9 PE=1 SV=1 - [S10A9_HUMAN]	56.14	1	4	4	13	5.38	24.56	2	2	34.35	56.14	4	11	114	13.2	6.13
P27169	Serum paraoxonase/arylesterase 1 OS=Homo sapiens OX=9606 GN=PON1 PE=1 SV=3 - [PON1_HUMAN]	54.08	2	11	11	105	153.97	52.11	10	52	146.57	47.89	10	53	355	39.7	5.22
P0C0S5	Histone H2A.Z OS=Homo sapiens OX=9606 GN=H2AZ1 PE=1 SV=2 - [H2AZ_HUMAN]	53.91	2	3	5	43		0.00			101.26	53.91	5	43	128	13.5	10.58
A0A075B6S5	Immunoglobulin kappa variable 1-27 OS=Homo sapiens OX=9606 GN=IGKV1-27 PE=3 SV=1 - [KV127_HUMAN]	53.85	1	2	3	4	4.11	13.68	1	1	10.98	53.85	3	3	117	12.7	8.29
P68871	Hemoglobin subunit beta OS=Homo sapiens OX=9606 GN=HBB PE=1 SV=2 - [HBB_HUMAN]	53.74	4	4	6	93	29.98	28.57	3	10	227.71	53.74	6	83	147	16.0	7.28
P01860	Immunoglobulin heavy constant gamma 3 OS=Homo sapiens OX=9606 GN=IGHG3 PE=1 SV=2 - [IGHG3_HUMAN]	52.79	1	6	15	420	418.45	41.91	13	166	737.95	52.79	15	254	377	41.3	7.90
P04406	Glyceraldehyde-3-phosphate dehydrogenase OS=Homo sapiens OX=9606 GN=GAPDH PE=1 SV=3 - [G3P_HUMAN]	51.64	1	11	11	45	13.44	13.43	3	5	131.09	51.64	11	40	335	36.0	8.46
P04003	C4b-binding protein alpha chain OS=Homo sapiens OX=9606 GN=C4BPA PE=1 SV=2 - [C4BPA_HUMAN]	51.26	1	23	23	192	345.54	51.26	22	123	192.90	45.23	18	69	597	67.0	7.30
P13645	Keratin, type I cytoskeletal 10 OS=Homo sapiens OX=9606 GN=KRT10 PE=1 SV=6 - [K1C10_HUMAN]	51.20	7	22	23	211	454.34	51.20	22	133	263.82	47.26	18	78	584	58.8	5.21
P01861	Immunoglobulin heavy constant gamma 4 OS=Homo sapiens OX=9606 GN=IGHG4 PE=1 SV=1 - [IGHG4_HUMAN]	51.07	1	4	11	264	326.93	51.07	11	127	396.26	45.87	10	137	327	35.9	7.36
P00450	Ceruloplasmin OS=Homo sapiens OX=9606 GN=CP PE=1 SV=1 - [CERU_HUMAN]	50.80	1	34	34	293	611.41	50.80	34	188	324.67	40.00	25	105	1065	122.1	5.72
P00738	Haptoglobin OS=Homo sapiens OX=9606 GN=HP PE=1 SV=1 - [HPT_HUMAN]	50.74	1	8	21	182	218.89	48.77	18	92	232.08	46.80	17	90	406	45.2	6.58
A0A0C4DH38	Immunoglobulin heavy variable 5-51 OS=Homo sapiens OX=9606 GN=IGHV5-51 PE=3 SV=1 - [HV551_HUMAN]	50.43	2	4	4	24	11.32	37.61	3	4	58.94	50.43	4	20	117	12.7	8.27
P01023	Alpha-2-macroglobulin OS=Homo sapiens OX=9606 GN=A2M PE=1 SV=3 - [A2MG_HUMAN]	50.41	2	48	48	303	237.92	32.84	29	88	634.87	50.34	47	215	1474	163.2	6.46
P01859	Immunoglobulin heavy constant gamma 2 OS=Homo sapiens OX=9606 GN=IGHG2 PE=1 SV=2 - [IGHG2_HUMAN]	50.31	1	5	12	294	402.85	50.31	12	153	377.89	50.31	12	141	326	35.9	7.59
P0DJI9	Serum amyloid A-2 protein OS=Homo sapiens OX=9606 GN=SAA2 PE=1 SV=1 - [SAA2_HUMAN]	50.00	1	3	7	39	4.93	11.48	1	2	112.32	50.00	7	37	122	13.5	9.14
P0DOX7	Immunoglobulin kappa light chain OS=Homo sapiens OX=9606 PE=1 SV=1 - [IGK_HUMAN]	49.53	1	2	8	420	466.09	49.53	8	134	895.12	49.53	8	286	214	23.4	7.17
P00352	Retinal dehydrogenase 1 OS=Homo sapiens OX=9606 GN=ALDH1A1 PE=1 SV=2 - [AL1A1_HUMAN]	49.50	1	15	15	46		0.00			150.35	49.50	15	46	501	54.8	6.73
P69905	Hemoglobin subunit alpha OS=Homo sapiens OX=9606 GN=HBA1 PE=1 SV=2 - [HBA_HUMAN]	49.30	1	5	5	114	15.55	17.61	2	11	252.91	49.30	5	103	142	15.2	8.68
P31327	Carbamoyl-phosphate synthase [ammonia], mitochondrial OS=Homo sapiens OX=9606 GN=CPS1 PE=1 SV=2 - [CPSM_HUM	49.20	2	47	47	237		0.00			675.52	49.20	47	237	1500	164.8	6.74
P23528	Cofilin-1 OS=Homo sapiens OX=9606 GN=CFL1 PE=1 SV=3 - [COF1_HUMAN]	48.19	3	5	5	18	35.40	43.98	4	10	23.36	39.76	4	8	166	18.5	8.09
P02749	Beta-2-glycoprotein 1 OS=Homo sapiens OX=9606 GN=APOH PE=1 SV=3 - [APOH_HUMAN]	48.12	1	12	12	73	147.61	48.12	12	51	70.21	33.91	7	22	345	38.3	7.97
P02652	Apolipoprotein A-II OS=Homo sapiens OX=9606 GN=APOA2 PE=1 SV=1 - [APOA2_HUMAN]	48.00	1	4	4	38	79.90	48.00	4	22	59.04	47.00	3	16	100	11.2	6.62
P16104	Histone H2AX OS=Homo sapiens OX=9606 GN=H2AX PE=1 SV=2 - [H2AX_HUMAN]	47.55	3	2	4	44		0.00			117.91	47.55	4	44	143	15.1	10.74
P0DOX2	Immunoglobulin alpha-2 heavy chain OS=Homo sapiens OX=9606 PE=1 SV=2 - [IGA2_HUMAN]	46.59	6	7	11	136	192.61	42.42	10	61	249.60	37.36	9	75	455	48.9	6.67
P01714	Immunoglobulin lambda variable 3-19 OS=Homo sapiens OX=9606 GN=IGLV3-19 PE=1 SV=2 - [LV319_HUMAN]	46.43	1	2	2	3					8.99	46.43	2	3	112	12.0	4.96
P00747	Plasminogen OS=Homo sapiens OX=9606 GN=PLG PE=1 SV=2 - [PLMN_HUMAN]	46.05	2	25	25	141	257.83	43.83	23	83	160.59	38.02	19	58	810	90.5	7.24
P0C0L5	Complement C4-B OS=Homo sapiens OX=9606 GN=C4B PE=1 SV=2 - [CO4B_HUMAN]	45.99	1	3	52	310	716.15	44.27	50	231	267.21	32.05	33	79	1744	192.6	7.27
P01614	Immunoglobulin kappa variable 2D-40 OS=Homo sapiens OX=9606 GN=IGKV2D-40 PE=1 SV=2 - [KVD40_HUMAN]	45.45	6	1	2	16	10.28	45.45	2	3	38.98	10.74	1	13	121	13.3	4.61
P0C0L4	Complement C4-A OS=Homo sapiens OX=9606 GN=C4A PE=1 SV=2 - [CO4A_HUMAN]	45.24	1	2	51	309	705.06	43.52	49	228	271.60	32.05	33	81	1744	192.7	7.08
P01008	Antithrombin-III OS=Homo sapiens OX=9606 GN=SERPINC1 PE=1 SV=1 - [ANT3_HUMAN]	45.04	1	18	18	59	141.52	45.04	18	53	17.09	16.59	4	6	464	52.6	6.71
P02790	Hemopexin OS=Homo sapiens OX=9606 GN=HPX PE=1 SV=2 - [HEMO_HUMAN]	44.16	1	14	14	98	162.65	44.16	13	53	141.11	38.10	11	45	462	51.6	7.02
P02765	Alpha-2-HS-glycoprotein OS=Homo sapiens OX=9606 GN=AHSG PE=1 SV=2 - [FETUA_HUMAN]	43.60	1	9	9	251	533.31	43.60	9	145	378.62	43.60	9	106	367	39.3	5.72
P08697	Alpha-2-antiplasmin OS=Homo sapiens OX=9606 GN=SERPINF2 PE=1 SV=3 - [A2AP_HUMAN]	43.58	1	12	12	52	72.77	29.53	9	26	88.16	34.42	9	26	491	54.5	6.29
P02655	Apolipoprotein C-II OS=Homo sapiens OX=9606 GN=APOC2 PE=1 SV=1 - [APOC2_HUMAN]	43.56	1	4	4	33	74.44	43.56	4	25	23.28	32.67	3	8	101	11.3	4.72
P00325	All-trans-retinol dehydrogenase [NAD(+)] ADH1B OS=Homo sapiens OX=9606 GN=ADH1B PE=1 SV=2 - [ADH1B_HUMAN]	43.20	1	4	12	70		0.00			227.57	43.20	12	70	375	39.8	8.29
O75636	Ficolin-3 OS=Homo sapiens OX=9606 GN=FCN3 PE=1 SV=2 - [FCN3_HUMAN]	43.14	1	9	9	81	13.85	23.41	3	4	253.83	43.14	9	77	299	32.9	6.67
P05062	Fructose-bisphosphate aldolase B OS=Homo sapiens OX=9606 GN=ALDOB PE=1 SV=2 - [ALDOB_HUMAN]	43.13	1	9	9	36		0.00			110.84	43.13	9	36	364	39.4	7.87
P06727	Apolipoprotein A-IV OS=Homo sapiens OX=9606 GN=APOA4 PE=1 SV=4 - [APOA4_HUMAN]	42.93	1	17	17	92	142.82	42.93	17	58	80.31	35.10	13	34	396	45.3	5.38
P59665	Neutrophil defensin 1 OS=Homo sapiens OX=9606 GN=DEFA1 PE=1 SV=1 - [DEF1_HUMAN]	42.55	2	2	2	3	0.00	9.57	1	1	8.87	32.98	1	2	94	10.2	6.99
P02748	Complement component C9 OS=Homo sapiens OX=9606 GN=C9 PE=1 SV=2 - [CO9_HUMAN]	42.22	1	16	16	99	169.95	37.75	15	63	105.58	31.13	11	36	559	63.1	5.59
Q86T20	Small integral membrane protein 29 OS=Homo sapiens OX=9606 GN=SMIM29 PE=2 SV=3 - [SIM29_HUMAN]	42.16	1	1	1	1	2.83	42.16	1	1		0.00			102	11.5	7.05
O75874	Isocitrate dehydrogenase [NADP] cytoplasmic OS=Homo sapiens OX=9606 GN=IDH1 PE=1 SV=2 - [IDHC_HUMAN]	41.79	2	13	13	47		0.00			104.55	41.79	13	47	414	46.6	7.01
P00734	Prothrombin OS=Homo sapiens OX=9606 GN=F2 PE=1 SV=2 - [THRB_HUMAN]	41.48	1	18	18	126	256.39	41.48	17	80	129.88	36.98	15	46	622	70.0	5.90
P08519	Apolipoprotein(a) OS=Homo sapiens OX=9606 GN=LPA PE=1 SV=1 - [APOA_HUMAN]	41.29	2	41	41	528	1233.20	41.29	41	417	346.28	37.40	25	111	4548	501.0	5.88
P06396	Gelsolin OS=Homo sapiens OX=9606 GN=GSN PE=1 SV=1 - [GELS_HUMAN]	41.18	1	17	17	97	190.17	38.11	16	60	133.96	32.35	13	37	782	85.6	6.28
Q14624	Inter-alpha-trypsin inhibitor heavy chain H4 OS=Homo sapiens OX=9606 GN=ITIH4 PE=1 SV=4 - [ITIH4_HUMAN]	40.97	1	28	28	162	330.69	36.45	26	109	157.95	31.61	18	53	930	103.3	6.98
Q96PD5	N-acetylmuramoyl-L-alanine amidase OS=Homo sapiens OX=9606 GN=PGLYRP2 PE=1 SV=1 - [PGRP2_HUMAN]	40.80	1	12	12	54	109.82	40.80	12	36	61.80	30.73	8	18	576	62.2	7.55
P01619	Immunoglobulin kappa variable 3-20 OS=Homo sapiens OX=9606 GN=IGKV3-20 PE=1 SV=2 - [KV320_HUMAN]	40.52	1	2	3	29	42.34	32.76	2	13	55.64	40.52	3	16	116	12.5	4.96
P02774	Vitamin D-binding protein OS=Homo sapiens OX=9606 GN=GC PE=1 SV=2 - [VTDB_HUMAN]	40.51	1	14	14	84	147.92	40.51	14	54	86.31	29.96	9	30	474	52.9	5.45
P01615	Immunoglobulin kappa variable 2D-28 OS=Homo sapiens OX=9606 GN=IGKV2D-28 PE=1 SV=2 - [KVD28_HUMAN]	40.00	6	1	2	18	9.90	40.00	2	3	49.39	40.00	2	15	120	12.9	5.94
O43866	CD5 antigen-like OS=Homo sapiens OX=9606 GN=CD5L PE=1 SV=1 - [CD5L_HUMAN]	39.48	1	9	9	54	55.49	29.39	6	19	101.85	39.48	9	35	347	38.1	5.47
A0A0B4J2H0	Immunoglobulin heavy variable 1-69D OS=Homo sapiens OX=9606 GN=IGHV1-69D PE=1 SV=1 - [HV69D_HUMAN]	39.32	3	1	3	19	14.30	9.40	1	6	38.46	39.32	3	13	117	12.7	8.47
P80748	Immunoglobulin lambda variable 3-21 OS=Homo sapiens OX=9606 GN=IGLV3-21 PE=1 SV=2 - [LV321_HUMAN]	39.32	2	2	2	18	22.87	13.68	1	7	42.10	39.32	2	11	117	12.4	5.29
P04264	Keratin, type II cytoskeletal 1 OS=Homo sapiens OX=9606 GN=KRT1 PE=1 SV=6 - [K2C1_HUMAN]	39.29	7	17	19	184	350.63	38.51	18	115	201.39	37.58	18	69	644	66.0	8.12
P02746	Complement C1q subcomponent subunit B OS=Homo sapiens OX=9606 GN=C1QB PE=1 SV=3 - [C1QB_HUMAN]	38.74	1	6	6	55	40.68	29.25	5	13	126.19	38.74	6	42	253	26.7	8.63
P06733	Alpha-enolase OS=Homo sapiens OX=9606 GN=ENO1 PE=1 SV=2 - [ENOA_HUMAN]	38.25	2	8	11	65	5.63	4.15	1	2	189.09	38.25	11	63	434	47.1	7.39
P07360	Complement component C8 gamma chain OS=Homo sapiens OX=9606 GN=C8G PE=1 SV=3 - [CO8G_HUMAN]	37.62	1	6	6	19	31.91	37.62	6	11	23.26	32.18	4	8	202	22.3	8.31
Q16851	UTP--glucose-1-phosphate uridylyltransferase OS=Homo sapiens OX=9606 GN=UGP2 PE=1 SV=5 - [UGPA_HUMAN]	37.60	1	12	12	43		0.00			129.63	37.60	12	43	508	56.9	8.15
P00736	Complement C1r subcomponent OS=Homo sapiens OX=9606 GN=C1R PE=1 SV=2 - [C1R_HUMAN]	37.59	2	18	18	114	156.97	33.62	15	52	187.82	37.59	18	62	705	80.1	6.21
P01706	Immunoglobulin lambda variable 2-11 OS=Homo sapiens OX=9606 GN=IGLV2-11 PE=1 SV=2 - [LV211_HUMAN]	36.97	1	2	2	4	3.20	30.25	1	1	7.76	36.97	2	3	119	12.6	7.24
P01011	Alpha-1-antichymotrypsin OS=Homo sapiens OX=9606 GN=SERPINA3 PE=1 SV=2 - [AACT_HUMAN]	36.88	1	12	12	95	91.51	31.44	10	35	184.54	36.88	12	60	423	47.6	5.52
P68431	Histone H3.1 OS=Homo sapiens OX=9606 GN=H3C1 PE=1 SV=2 - [H31_HUMAN]	36.76	3	1	3	25		0.00			66.95	36.76	3	25	136	15.4	11.12
P84243	Histone H3.3 OS=Homo sapiens OX=9606 GN=H3-3A PE=1 SV=2 - [H33_HUMAN]	36.76	3	1	3	29		0.00			93.50	36.76	3	29	136	15.3	11.27
Q71DI3	Histone H3.2 OS=Homo sapiens OX=9606 GN=H3C15 PE=1 SV=3 - [H32_HUMAN]	36.76	3	1	3	34					111.99	36.76	3	34	136	15.4	11.27
P35908	Keratin, type II cytoskeletal 2 epidermal OS=Homo sapiens OX=9606 GN=KRT2 PE=1 SV=2 - [K22E_HUMAN]	36.62	9	17	19	83	111.41	31.46	14	46	99.04	31.14	16	37	639	65.4	8.00
P02656	Apolipoprotein C-III OS=Homo sapiens OX=9606 GN=APOC3 PE=1 SV=1 - [APOC3_HUMAN]	36.36	1	4	4	92	189.68	36.36	4	63	91.71	34.34	2	29	99	10.8	5.41
O95445	Apolipoprotein M OS=Homo sapiens OX=9606 GN=APOM PE=1 SV=2 - [APOM_HUMAN]	36.17	1	4	4	18	12.30	36.17	4	11	3.47	3.72	1	7	188	21.2	6.01
P10643	Complement component C7 OS=Homo sapiens OX=9606 GN=C7 PE=1 SV=2 - [CO7_HUMAN]	35.94	1	18	18	76	39.04	15.66	8	16	191.13	35.94	18	60	843	93.5	6.48
P01042	Kininogen-1 OS=Homo sapiens OX=9606 GN=KNG1 PE=1 SV=2 - [KNG1_HUMAN]	35.87	1	16	16	89	146.96	33.07	15	50	116.61	29.97	14	39	644	71.9	6.81
P27105	Stomatin OS=Homo sapiens OX=9606 GN=STOM PE=1 SV=3 - [STOM_HUMAN]	35.76	1	6	6	25	18.14	20.49	4	7	59.08	35.76	6	18	288	31.7	7.88
P06899	Histone H2B type 1-J OS=Homo sapiens OX=9606 GN=H2BC11 PE=1 SV=3 - [H2B1J_HUMAN]	35.71	9	1	4	112	0.00	8.73	1	2	257.84	26.98	3	110	126	13.9	10.32
O60814	Histone H2B type 1-K OS=Homo sapiens OX=9606 GN=H2BC12 PE=1 SV=3 - [H2B1K_HUMAN]	35.71	11	2	5	123	0.00	8.73	1	2	288.02	26.98	4	121	126	13.9	10.32
P19652	Alpha-1-acid glycoprotein 2 OS=Homo sapiens OX=9606 GN=ORM2 PE=1 SV=2 - [A1AG2_HUMAN]	35.32	1	3	5	31	33.22	35.32	5	15	33.50	35.32	5	16	201	23.6	5.11
P04217	Alpha-1B-glycoprotein OS=Homo sapiens OX=9606 GN=A1BG PE=1 SV=4 - [A1BG_HUMAN]	35.15	1	10	10	44	51.69	28.89	8	17	78.66	33.33	9	27	495	54.2	5.86
Q9Y315	Deoxyribose-phosphate aldolase OS=Homo sapiens OX=9606 GN=DERA PE=1 SV=2 - [DEOC_HUMAN]	34.91	1	7	7	13		0.00			35.82	34.91	7	13	318	35.2	8.94
P01782	Immunoglobulin heavy variable 3-9 OS=Homo sapiens OX=9606 GN=IGHV3-9 PE=1 SV=2 - [HV309_HUMAN]	34.75	5	3	3	7					17.89	34.75	3	7	118	12.9	7.08
P05090	Apolipoprotein D OS=Homo sapiens OX=9606 GN=APOD PE=1 SV=1 - [APOD_HUMAN]	34.39	1	8	8	89	144.48	26.46	5	47	118.41	34.39	7	42	189	21.3	5.15
P02671	Fibrinogen alpha chain OS=Homo sapiens OX=9606 GN=FGA PE=1 SV=2 - [FIBA_HUMAN]	33.72	1	26	26	899	1446.13	33.72	25	513	1128.98	29.68	21	386	866	94.9	6.01
P04004	Vitronectin OS=Homo sapiens OX=9606 GN=VTN PE=1 SV=1 - [VTNC_HUMAN]	33.68	1	10	10	88	189.97	33.68	10	66	64.31	20.29	6	22	478	54.3	5.80
P00751	Complement factor B OS=Homo sapiens OX=9606 GN=CFB PE=1 SV=2 - [CFAB_HUMAN]	33.64	1	17	17	82	150.70	33.64	17	50	98.16	23.17	12	32	764	85.5	7.06
P30041	Peroxiredoxin-6 OS=Homo sapiens OX=9606 GN=PRDX6 PE=1 SV=3 - [PRDX6_HUMAN]	33.48	1	5	5	14		0.00			40.78	33.48	5	14	224	25.0	6.38
P01742	Immunoglobulin heavy variable 1-69 OS=Homo sapiens OX=9606 GN=IGHV1-69 PE=1 SV=2 - [HV169_HUMAN]	33.33	3	1	3	22	14.30	9.40	1	6	45.82	33.33	3	16	117	12.7	9.17
P02763	Alpha-1-acid glycoprotein 1 OS=Homo sapiens OX=9606 GN=ORM1 PE=1 SV=1 - [A1AG1_HUMAN]	33.33	1	4	6	58	39.49	27.36	5	22	67.79	33.33	6	36	201	23.5	5.02
P02760	Protein AMBP OS=Homo sapiens OX=9606 GN=AMBP PE=1 SV=1 - [AMBP_HUMAN]	32.95	1	7	7	57	102.86	32.95	7	33	67.24	26.42	6	24	352	39.0	6.25
P05166	Propionyl-CoA carboxylase beta chain, mitochondrial OS=Homo sapiens OX=9606 GN=PCCB PE=1 SV=3 - [PCCB_HUMAN]	32.65	1	10	10	21		0.00			59.32	32.65	10	21	539	58.2	7.64
P32119	Peroxiredoxin-2 OS=Homo sapiens OX=9606 GN=PRDX2 PE=1 SV=5 - [PRDX2_HUMAN]	32.32	2	5	5	10		0.00			23.15	32.32	5	10	198	21.9	5.97
A0A0C4DH67	Immunoglobulin kappa variable 1-8 OS=Homo sapiens OX=9606 GN=IGKV1-8 PE=3 SV=1 - [KV108_HUMAN]	32.17	2	2	3	5	4.11	13.91	1	1	14.55	32.17	3	4	115	12.5	9.01
P68366	Tubulin alpha-4A chain OS=Homo sapiens OX=9606 GN=TUBA4A PE=1 SV=1 - [TBA4A_HUMAN]	31.92	2	3	8	18	14.84	13.17	3	5	35.15	31.92	8	13	448	49.9	5.06
P07358	Complement component C8 beta chain OS=Homo sapiens OX=9606 GN=C8B PE=1 SV=3 - [CO8B_HUMAN]	31.81	1	13	13	46	68.06	23.52	10	28	39.92	26.57	11	18	591	67.0	8.13
A0A0C4DH73	Immunoglobulin kappa variable 1-12 OS=Homo sapiens OX=9606 GN=IGKV1-12 PE=3 SV=1 - [KV112_HUMAN]	31.62	4	3	3	15	12.43	13.68	1	4	38.63	31.62	3	11	117	12.6	8.31
P01701	Immunoglobulin lambda variable 1-51 OS=Homo sapiens OX=9606 GN=IGLV1-51 PE=1 SV=2 - [LV151_HUMAN]	31.62	1	2	2	2	0.00	6.84	1	1	2.85	24.79	1	1	117	12.2	7.03
P04430	Immunoglobulin kappa variable 1-16 OS=Homo sapiens OX=9606 GN=IGKV1-16 PE=1 SV=2 - [KV116_HUMAN]	31.62	1	2	2	5	4.95	13.68	1	2	7.47	31.62	2	3	117	12.6	8.16
P19823	Inter-alpha-trypsin inhibitor heavy chain H2 OS=Homo sapiens OX=9606 GN=ITIH2 PE=1 SV=2 - [ITIH2_HUMAN]	31.29	1	18	18	144	292.27	31.29	18	88	177.77	23.47	11	56	946	106.4	6.86
P18428	Lipopolysaccharide-binding protein OS=Homo sapiens OX=9606 GN=LBP PE=1 SV=3 - [LBP_HUMAN]	30.98	1	11	11	78	77.24	28.07	9	26	174.21	28.90	10	52	481	53.3	6.70
P0DPH7	Tubulin alpha-3C chain OS=Homo sapiens OX=9606 GN=TUBA3C PE=1 SV=1 - [TBA3C_HUMAN]	30.89	5	3	8	21	14.84	13.11	3	5	41.33	30.89	8	16	450	49.9	5.10
P08603	Complement factor H OS=Homo sapiens OX=9606 GN=CFH PE=1 SV=4 - [CFAH_HUMAN]	30.79	2	23	23	80	145.72	26.81	20	53	68.08	21.69	16	27	1231	139.0	6.61
P31151	Protein S100-A7 OS=Homo sapiens OX=9606 GN=S100A7 PE=1 SV=4 - [S10A7_HUMAN]	30.69	2	2	2	4	8.08	30.69	2	4		0.00			101	11.5	6.77
P07327	Alcohol dehydrogenase 1A OS=Homo sapiens OX=9606 GN=ADH1A PE=1 SV=2 - [ADH1A_HUMAN]	30.67	1	3	10	40		0.00			124.70	30.67	10	40	375	39.8	7.94
P02747	Complement C1q subcomponent subunit C OS=Homo sapiens OX=9606 GN=C1QC PE=1 SV=3 - [C1QC_HUMAN]	30.61	1	5	5	59	40.59	25.71	4	14	120.89	30.61	5	45	245	25.8	8.41
P02042	Hemoglobin subunit delta OS=Homo sapiens OX=9606 GN=HBD PE=1 SV=2 - [HBD_HUMAN]	30.61	4	1	3	23	6.82	6.80	1	3	44.78	30.61	3	20	147	16.0	8.05
Q9HD89	Resistin OS=Homo sapiens OX=9606 GN=RETN PE=1 SV=1 - [RETN_HUMAN]	30.56	1	2	2	3		0.00			9.36	30.56	2	3	108	11.4	6.86
P02753	Retinol-binding protein 4 OS=Homo sapiens OX=9606 GN=RBP4 PE=1 SV=3 - [RET4_HUMAN]	30.35	1	4	4	8	18.77	30.35	4	7	0.00	5.47	1	1	201	23.0	6.07
P07099	Epoxide hydrolase 1 OS=Homo sapiens OX=9606 GN=EPHX1 PE=1 SV=1 - [HYEP_HUMAN]	30.33	1	7	7	10		0.00			24.68	30.33	7	10	455	52.9	7.25
P01019	Angiotensinogen OS=Homo sapiens OX=9606 GN=AGT PE=1 SV=1 - [ANGT_HUMAN]	30.31	1	9	9	56	70.80	22.47	8	29	84.65	28.45	8	27	485	53.1	6.32
P06331	Immunoglobulin heavy variable 4-34 OS=Homo sapiens OX=9606 GN=IGHV4-34 PE=1 SV=2 - [HV434_HUMAN]	30.08	9	2	2	20	19.59	13.01	1	6	42.76	30.08	2	14	123	13.8	9.33
P63104	14-3-3 protein zeta/delta OS=Homo sapiens OX=9606 GN=YWHAZ PE=1 SV=1 - [1433Z_HUMAN]	29.80	7	5	5	10	8.92	18.37	3	3	19.67	22.04	4	7	245	27.7	4.79
P0DMV8	Heat shock 70 kDa protein 1A OS=Homo sapiens OX=9606 GN=HSPA1A PE=1 SV=1 - [HS71A_HUMAN]	29.64	4	11	11	23		0.00			71.43	29.64	11	23	641	70.0	5.66
Q01629	Interferon-induced transmembrane protein 2 OS=Homo sapiens OX=9606 GN=IFITM2 PE=1 SV=2 - [IFM2_HUMAN]	29.55	3	2	2	2		0.00			5.82	29.55	2	2	132	14.6	7.42
P02730	Band 3 anion transport protein OS=Homo sapiens OX=9606 GN=SLC4A1 PE=1 SV=3 - [B3AT_HUMAN]	29.42	1	16	16	79	7.68	8.01	3	3	232.49	29.42	16	76	911	101.7	5.19
Q00796	Sorbitol dehydrogenase OS=Homo sapiens OX=9606 GN=SORD PE=1 SV=4 - [DHSO_HUMAN]	29.41	1	6	6	21		0.00			71.47	29.41	6	21	357	38.3	7.97
P20851	C4b-binding protein beta chain OS=Homo sapiens OX=9606 GN=C4BPB PE=1 SV=1 - [C4BPB_HUMAN]	29.37	1	6	6	18	37.60	29.37	6	15	6.56	15.08	3	3	252	28.3	5.14
P19827	Inter-alpha-trypsin inhibitor heavy chain H1 OS=Homo sapiens OX=9606 GN=ITIH1 PE=1 SV=3 - [ITIH1_HUMAN]	29.31	1	18	18	87	206.29	27.88	17	65	69.19	18.11	10	22	911	101.3	6.79
P02751	Fibronectin OS=Homo sapiens OX=9606 GN=FN1 PE=1 SV=5 - [FINC_HUMAN]	28.99	1	39	39	226	266.55	20.39	28	86	477.87	27.82	37	140	2477	272.2	5.50
Q14520	Hyaluronan-binding protein 2 OS=Homo sapiens OX=9606 GN=HABP2 PE=1 SV=1 - [HABP2_HUMAN]	28.93	1	12	12	77	25.60	12.68	4	11	170.15	28.93	12	66	560	62.6	6.54
P60660	Myosin light polypeptide 6 OS=Homo sapiens OX=9606 GN=MYL6 PE=1 SV=2 - [MYL6_HUMAN]	28.48	3	3	3	5	4.63	18.54	2	2	8.06	20.53	2	3	151	16.9	4.65
P13929	Beta-enolase OS=Homo sapiens OX=9606 GN=ENO3 PE=1 SV=5 - [ENOB_HUMAN]	28.34	2	4	7	48	5.63	4.15	1	2	153.39	28.34	7	46	434	47.0	7.71
P10909	Clusterin OS=Homo sapiens OX=9606 GN=CLU PE=1 SV=1 - [CLUS_HUMAN]	27.39	1	11	11	117	218.78	27.39	11	74	146.49	23.16	7	43	449	52.5	6.27
P07357	Complement component C8 alpha chain OS=Homo sapiens OX=9606 GN=C8A PE=1 SV=2 - [CO8A_HUMAN]	27.23	1	9	9	29	55.39	27.23	9	18	32.35	15.58	5	11	584	65.1	6.47
P07737	Profilin-1 OS=Homo sapiens OX=9606 GN=PFN1 PE=1 SV=2 - [PROF1_HUMAN]	27.14	1	3	3	7		0.00			13.73	27.14	3	7	140	15.0	8.27
P01031	Complement C5 OS=Homo sapiens OX=9606 GN=C5 PE=1 SV=4 - [CO5_HUMAN]	26.91	1	30	30	77	109.37	22.08	25	46	77.08	19.87	20	31	1676	188.2	6.52
P05546	Heparin cofactor 2 OS=Homo sapiens OX=9606 GN=SERPIND1 PE=1 SV=3 - [HEP2_HUMAN]	26.45	1	11	11	75	115.86	26.45	11	50	54.22	21.24	8	25	499	57.0	6.90
P35542	Serum amyloid A-4 protein OS=Homo sapiens OX=9606 GN=SAA4 PE=1 SV=2 - [SAA4_HUMAN]	26.15	1	3	3	25	27.20	26.15	3	10	41.48	26.15	3	15	130	14.7	9.07
P31025	Lipocalin-1 OS=Homo sapiens OX=9606 GN=LCN1 PE=1 SV=1 - [LCN1_HUMAN]	26.14	2	4	4	31	44.02	22.16	3	27	5.09	16.48	3	4	176	19.2	5.58
P00167	Cytochrome b5 OS=Homo sapiens OX=9606 GN=CYB5A PE=1 SV=2 - [CYB5_HUMAN]	26.12	1	2	2	4					11.12	26.12	2	4	134	15.3	4.96
P01624	Immunoglobulin kappa variable 3-15 OS=Homo sapiens OX=9606 GN=IGKV3-15 PE=1 SV=2 - [KV315_HUMAN]	26.09	4	2	2	15	8.36	26.09	2	3	30.43	26.09	2	12	115	12.5	5.19
A0A0A0MRZ8	Immunoglobulin kappa variable 3D-11 OS=Homo sapiens OX=9606 GN=IGKV3D-11 PE=3 SV=6 - [KVD11_HUMAN]	26.09	2	2	2	8	7.27	18.26	1	2	15.26	26.09	2	6	115	12.6	5.29
P0DP02	Immunoglobulin heavy variable 3-30-3 OS=Homo sapiens OX=9606 GN=IGHV3-30-3 PE=3 SV=1 - [HVC33_HUMAN]	25.64	6	1	2	25	33.19	9.40	1	11	39.17	25.64	2	14	117	13.0	8.94
P00966	Argininosuccinate synthase OS=Homo sapiens OX=9606 GN=ASS1 PE=1 SV=2 - [ASSY_HUMAN]	25.49	1	6	6	12		0.00			37.59	25.49	6	12	412	46.5	8.02
P04196	Histidine-rich glycoprotein OS=Homo sapiens OX=9606 GN=HRG PE=1 SV=1 - [HRG_HUMAN]	25.14	1	11	11	55	116.72	25.14	11	40	41.05	18.29	7	15	525	59.5	7.50
P55058	Phospholipid transfer protein OS=Homo sapiens OX=9606 GN=PLTP PE=1 SV=1 - [PLTP_HUMAN]	24.95	1	8	8	39	4.42	3.04	1	4	114.66	24.95	8	35	493	54.7	7.01
P05109	Protein S100-A8 OS=Homo sapiens OX=9606 GN=S100A8 PE=1 SV=1 - [S10A8_HUMAN]	24.73	1	2	2	6					15.81	24.73	2	6	93	10.8	7.03
P10809	60 kDa heat shock protein, mitochondrial OS=Homo sapiens OX=9606 GN=HSPD1 PE=1 SV=2 - [CH60_HUMAN]	24.61	1	9	9	22		0.00			69.09	24.61	9	22	573	61.0	5.87
A1L3X4	Putative metallothionein MT1DP OS=Homo sapiens OX=9606 GN=MT1DP PE=5 SV=1 - [MT1DP_HUMAN]	24.49	1	1	1	1		0.00			0.00	24.49	1	1	49	5.0	7.97
A0A0C4DH31	Immunoglobulin heavy variable 1-18 OS=Homo sapiens OX=9606 GN=IGHV1-18 PE=3 SV=1 - [HV118_HUMAN]	23.93	1	1	2	12	8.77	23.93	2	3	25.38	23.93	2	9	117	12.8	8.84
P13796	Plastin-2 OS=Homo sapiens OX=9606 GN=LCP1 PE=1 SV=6 - [PLSL_HUMAN]	23.44	1	8	8	15		0.00			41.06	23.44	8	15	627	70.2	5.43

Q15485	Ficolin-2 OS=Homo sapiens OX=9606 GN=FCN2 PE=1 SV=2 - [FCN2_HUMAN]	23.32	1	3	3	7		0.00			26.95	23.32	3	7	313	34.0	6.77
P80723	Brain acid soluble protein 1 OS=Homo sapiens OX=9606 GN=BASP1 PE=1 SV=2 - [BASP1_HUMAN]	22.91	1	2	2	6	5.64	22.91	2	2	16.97	22.91	2	4	227	22.7	4.63
P23141	Liver carboxylesterase 1 OS=Homo sapiens OX=9606 GN=CES1 PE=1 SV=2 - [EST1_HUMAN]	22.57	2	9	9	12		0.00			31.75	22.57	9	12	567	62.5	6.60
P59768	Guanine nucleotide-binding protein G(I)/G(S)/G(O) subunit gamma-2 OS=Homo sapiens OX=9606 GN=GNG2 PE=1 SV=2 -	22.54	1	1	1	1		0.00			2.68	22.54	1	1	71	7.8	7.99
Q12805	EGF-containing fibulin-like extracellular matrix protein 1 OS=Homo sapiens OX=9606 GN=EFEMP1 PE=1 SV=2 - [FBLN3_HU	22.52	1	7	7	14	3.10	4.67	1	1	29.08	22.52	7	13	493	54.6	5.07
P01743	Immunoglobulin heavy variable 1-46 OS=Homo sapiens OX=9606 GN=IGHV1-46 PE=1 SV=2 - [HV146_HUMAN]	22.22	3	1	2	18	14.30	9.40	1	6	32.85	22.22	2	12	117	12.9	8.92
P02786	Transferrin receptor protein 1 OS=Homo sapiens OX=9606 GN=TFRC PE=1 SV=2 - [TFR1_HUMAN]	21.97	1	12	12	36	2.23	1.32	1	1	109.06	21.97	12	35	760	84.8	6.61
P36957	Dihydrolipoyllysine-residue succinyltransferase component of 2-oxoglutarate dehydrogenase complex, mitochondrial OS=Ho	21.63	1	5	5	10		0.00			27.81	21.63	5	10	453	48.7	8.95
A0A0C4DH25	Immunoglobulin kappa variable 3D-20 OS=Homo sapiens OX=9606 GN=IGKV3D-20 PE=3 SV=1 - [KVD20_HUMAN]	21.55	1	1	2	26	38.10	21.55	2	13	43.01	21.55	2	13	116	12.5	4.59
P12273	Prolactin-inducible protein OS=Homo sapiens OX=9606 GN=PIP PE=1 SV=1 - [PIP_HUMAN]	21.23	1	2	2	4	12.06	21.23	2	4		0.00			146	16.6	8.05
P68032	Actin, alpha cardiac muscle 1 OS=Homo sapiens OX=9606 GN=ACTC1 PE=1 SV=1 - [ACTC_HUMAN]	21.22	8	1	7	88	91.04	21.22	7	37	132.27	19.36	6	51	377	42.0	5.39
P0CG48	Polyubiquitin-C OS=Homo sapiens OX=9606 GN=UBC PE=1 SV=3 - [UBC_HUMAN]	21.02	4	1	1	6	5.67	21.02	1	2	10.39	21.02	1	4	685	77.0	7.66
P07225	Vitamin K-dependent protein S OS=Homo sapiens OX=9606 GN=PROS1 PE=1 SV=1 - [PROS_HUMAN]	20.56	1	10	10	40	72.07	20.56	9	28	30.33	13.46	6	12	676	75.1	5.67
P09110	3-ketoacyl-CoA thiolase, peroxisomal OS=Homo sapiens OX=9606 GN=ACAA1 PE=1 SV=2 - [THIK_HUMAN]	20.52	1	6	6	15		0.00			39.19	20.52	6	15	424	44.3	8.44
P23083	Immunoglobulin heavy variable 1-2 OS=Homo sapiens OX=9606 GN=IGHV1-2 PE=1 SV=2 - [HV102_HUMAN]	20.51	1	1	2	10	5.85	9.40	1	2	20.16	20.51	2	8	117	13.1	9.13
P08185	Corticosteroid-binding globulin OS=Homo sapiens OX=9606 GN=SERPINA6 PE=1 SV=1 - [CBG_HUMAN]	20.49	1	4	4	11	13.65	16.05	3	5	19.92	14.32	3	6	405	45.1	6.04
P07900	Heat shock protein HSP 90-alpha OS=Homo sapiens OX=9606 GN=HSP90AA1 PE=1 SV=5 - [HS90A_HUMAN]	20.49	6	7	10	23		0.00			62.87	20.49	10	23	732	84.6	5.02
P02745	Complement C1q subcomponent subunit A OS=Homo sapiens OX=9606 GN=C1QA PE=1 SV=2 - [C1QA_HUMAN]	20.41	1	2	2	13	12.58	9.39	1	3	42.32	20.41	2	10	245	26.0	9.11
P04921	Glycophorin-C OS=Homo sapiens OX=9606 GN=GYPC PE=1 SV=1 - [GLPC_HUMAN]	20.31	1	1	1	3		0.00			9.62	20.31	1	3	128	13.8	4.84
P68371	Tubulin beta-4B chain OS=Homo sapiens OX=9606 GN=TUBB4B PE=1 SV=1 - [TBB4B_HUMAN]	20.22	9	5	5	7	2.80	3.82	1	1	16.54	20.22	5	6	445	49.8	4.89
P23142	Fibulin-1 OS=Homo sapiens OX=9606 GN=FBLN1 PE=1 SV=4 - [FBLN1_HUMAN]	19.91	1	9	9	28	15.98	10.24	5	7	60.01	18.35	8	21	703	77.2	5.22
P06312	Immunoglobulin kappa variable 4-1 OS=Homo sapiens OX=9606 GN=IGKV4-1 PE=1 SV=1 - [KV401_HUMAN]	19.83	1	3	3	10	9.36	19.83	2	4	11.84	19.83	3	6	121	13.4	5.25
Q9BTL3	RNA guanine-N7 methyltransferase activating subunit OS=Homo sapiens OX=9606 GN=RAMAC PE=1 SV=1 - [RAMAC_HUM	19.49	1	1	1	1	0.00	19.49	1	1		0.00			118	14.4	8.94
Q9Y490	Talin-1 OS=Homo sapiens OX=9606 GN=TLN1 PE=1 SV=3 - [TLN1_HUMAN]	19.48	1	23	23	37	57.61	13.66	17	22	41.75	10.31	13	15	2541	269.6	6.07
P22792	Carboxypeptidase N subunit 2 OS=Homo sapiens OX=9606 GN=CPN2 PE=1 SV=3 - [CPN2_HUMAN]	19.45	1	6	6	8	8.91	12.66	4	4	12.25	14.31	4	4	545	60.5	5.99
P04275	von Willebrand factor OS=Homo sapiens OX=9606 GN=VWF PE=1 SV=4 - [VWF_HUMAN]	19.45	1	31	31	88	3.31	0.53	1	1	250.16	19.45	31	87	2813	309.1	5.48
O14791	Apolipoprotein L1 OS=Homo sapiens OX=9606 GN=APOL1 PE=1 SV=5 - [APOL1_HUMAN]	19.35	1	5	5	47	60.46	19.35	5	22	68.07	19.35	5	25	398	43.9	5.81
Q08380	Galectin-3-binding protein OS=Homo sapiens OX=9606 GN=LGALS3BP PE=1 SV=1 - [LG3BP_HUMAN]	19.32	1	6	6	11		0.00			31.88	19.32	6	11	585	65.3	5.27
P09871	Complement C1s subcomponent OS=Homo sapiens OX=9606 GN=C1S PE=1 SV=1 - [C1S_HUMAN]	19.19	1	8	8	25	37.87	15.41	6	12	37.40	14.83	7	13	688	76.6	4.96
P24752	Acetyl-CoA acetyltransferase, mitochondrial OS=Homo sapiens OX=9606 GN=ACAT1 PE=1 SV=1 - [THIL_HUMAN]	18.97	1	5	5	8		0.00			23.06	18.97	5	8	427	45.2	8.85
P61626	Lysozyme C OS=Homo sapiens OX=9606 GN=LYZ PE=1 SV=1 - [LYSC_HUMAN]	18.92	1	1	1	4	7.12	18.92	1	2	8.36	18.92	1	2	148	16.5	9.16
Q9Y584	Mitochondrial import inner membrane translocase subunit Tim22 OS=Homo sapiens OX=9606 GN=TIMM22 PE=1 SV=2 - [T	18.56	1	1	1	1	3.39	18.56	1	1		0.00			194	20.0	7.59
P61769	Beta-2-microglobulin OS=Homo sapiens OX=9606 GN=B2M PE=1 SV=1 - [B2MG_HUMAN]	18.49	1	1	1	1		0.00			3.52	18.49	1	1	119	13.7	6.52
P14209	CD99 antigen OS=Homo sapiens OX=9606 GN=CD99 PE=1 SV=1 - [CD99_HUMAN]	18.38	1	1	1	1	0.00	18.38	1	1		0.00			185	18.8	4.75
Q9UGM5	Fetuin-B OS=Homo sapiens OX=9606 GN=FETUB PE=1 SV=2 - [FETUB_HUMAN]	18.32	1	4	4	13	18.26	18.32	4	7	12.04	18.32	4	6	382	42.0	6.83
P05165	Propionyl-CoA carboxylase alpha chain, mitochondrial OS=Homo sapiens OX=9606 GN=PCCA PE=1 SV=4 - [PCCA_HUMAN]	18.27	1	9	9	23		0.00			58.69	18.27	9	23	728	80.0	7.52
Q8WXA2	Prostate and testis expressed protein 1 OS=Homo sapiens OX=9606 GN=PATE1 PE=1 SV=1 - [PATE1_HUMAN]	18.25	1	1	1	1	0.00	18.25	1	1		0.00			126	14.3	7.93
P00326	Alcohol dehydrogenase 1C OS=Homo sapiens OX=9606 GN=ADH1C PE=1 SV=2 - [ADH1G_HUMAN]	18.13	1	1	4	19		0.00			53.75	18.13	4	19	375	39.8	8.29
P25311	Zinc-alpha-2-glycoprotein OS=Homo sapiens OX=9606 GN=AZGP1 PE=1 SV=2 - [ZA2G_HUMAN]	18.12	1	3	3	8	18.19	18.12	3	5	7.69	11.07	2	3	298	34.2	6.05
P29622	Kallistatin OS=Homo sapiens OX=9606 GN=SERPINA4 PE=1 SV=3 - [KAIN_HUMAN]	18.03	1	5	5	11	23.44	18.03	5	11		0.00			427	48.5	7.75
P01602	Immunoglobulin kappa variable 1-5 OS=Homo sapiens OX=9606 GN=IGKV1-5 PE=1 SV=2 - [KV105_HUMAN]	17.95	1	2	2	12	13.48	17.95	2	5	25.66	17.95	2	7	117	12.8	8.28
Q9BWD1	Acetyl-CoA acetyltransferase, cytosolic OS=Homo sapiens OX=9606 GN=ACAT2 PE=1 SV=2 - [THIC_HUMAN]	17.88	1	3	3	4		0.00			9.23	17.88	3	4	397	41.3	6.92
P11021	Endoplasmic reticulum chaperone BiP OS=Homo sapiens OX=9606 GN=HSPA5 PE=1 SV=2 - [BIP_HUMAN]	17.28	1	7	7	15		0.00			38.04	17.28	7	15	654	72.3	5.16
P01721	Immunoglobulin lambda variable 6-57 OS=Homo sapiens OX=9606 GN=IGLV6-57 PE=1 SV=2 - [LV657_HUMAN]	17.09	1	1	1	3		0.00			10.07	17.09	1	3	117	12.6	4.78
Q6W349	Putative uncharacterized protein encoded by LINC00575 OS=Homo sapiens OX=9606 GN=LINC00575 PE=5 SV=1 - [CD011_	17.02	1	1	1	1					3.16	17.02	1	1	94	10.1	5.67
Q9H2W1	Membrane-spanning 4-domains subfamily A member 6A OS=Homo sapiens OX=9606 GN=MS4A6A PE=1 SV=1 - [M4A6A_H	16.94	1	1	1	1		0.00			0.00	16.94	1	1	248	26.9	7.06
O60830	Mitochondrial import inner membrane translocase subunit Tim17-B OS=Homo sapiens OX=9606 GN=TIMM17B PE=1 SV=1	16.86	1	1	1	1					4.31	16.86	1	1	172	18.3	9.03
Q562R1	Beta-actin-like protein 2 OS=Homo sapiens OX=9606 GN=ACTBL2 PE=1 SV=2 - [ACTBL_HUMAN]	16.76	1	1	4	37	40.26	6.38	2	16	56.97	16.76	4	21	376	42.0	5.59
P0DOX3	Immunoglobulin delta heavy chain OS=Homo sapiens OX=9606 PE=1 SV=1 - [IGD_HUMAN]	16.60	2	6	6	14		0.00			38.84	16.60	6	14	512	56.2	8.02
P14618	Pyruvate kinase PKM OS=Homo sapiens OX=9606 GN=PKM PE=1 SV=4 - [KPYM_HUMAN]	16.57	1	5	6	7		0.00			19.44	16.57	6	7	531	57.9	7.84
P02741	C-reactive protein OS=Homo sapiens OX=9606 GN=CRP PE=1 SV=1 - [CRP_HUMAN]	16.52	1	4	4	14					37.45	16.52	4	14	224	25.0	5.63
P00915	Carbonic anhydrase 1 OS=Homo sapiens OX=9606 GN=CA1 PE=1 SV=2 - [CAH1_HUMAN]	16.48	1	2	2	2		0.00			5.28	16.48	2	2	261	28.9	7.12
P21333	Filamin-A OS=Homo sapiens OX=9606 GN=FLNA PE=1 SV=4 - [FLNA_HUMAN]	16.40	1	24	24	63	75.70	12.28	18	30	95.85	11.94	17	33	2647	280.6	6.06
Q5T292	Transmembrane protein 273 OS=Homo sapiens OX=9606 GN=TMEM273 PE=3 SV=1 - [TM273_HUMAN]	16.19	1	1	1	2	1.83	16.19	1	1	2.47	16.19	1	1	105	11.3	9.55
Q6ZP80	Transmembrane protein 182 OS=Homo sapiens OX=9606 GN=TMEM182 PE=1 SV=2 - [TM182_HUMAN]	16.16	1	1	1	1		0.00			4.30	16.16	1	1	229	25.9	6.93
Q9Y3A6	Transmembrane emp24 domain-containing protein 5 OS=Homo sapiens OX=9606 GN=TMED5 PE=1 SV=1 - [TMED5_HUMA	16.16	1	1	1	1					3.21	16.16	1	1	229	26.0	4.84
P35579	Myosin-9 OS=Homo sapiens OX=9606 GN=MYH9 PE=1 SV=4 - [MYH9_HUMAN]	16.07	3	16	19	40	40.00	8.01	10	16	70.21	12.91	15	24	1960	226.4	5.60
P04439	HLA class I histocompatibility antigen, A alpha chain OS=Homo sapiens OX=9606 GN=HLA-A PE=1 SV=2 - [HLAA_HUMAN]	15.62	2	3	3	3		0.00			8.18	15.62	3	3	365	40.8	6.00
Q06033	Inter-alpha-trypsin inhibitor heavy chain H3 OS=Homo sapiens OX=9606 GN=ITIH3 PE=1 SV=2 - [ITIH3_HUMAN]	15.62	1	8	8	22	33.88	12.02	6	11	26.45	13.26	6	11	890	99.8	5.74
P04278	Sex hormone-binding globulin OS=Homo sapiens OX=9606 GN=SHBG PE=1 SV=2 - [SHBG_HUMAN]	15.42	1	3	3	6	11.32	9.45	2	4	3.93	11.44	2	2	402	43.8	6.71
Q5XKR4	Homeobox protein orthopedia OS=Homo sapiens OX=9606 GN=OTP PE=1 SV=1 - [OTP_HUMAN]	15.38	1	1	1	1		0.00			4.41	15.38	1	1	325	34.1	9.47
A0A0B4J1X5	Immunoglobulin heavy variable 3-74 OS=Homo sapiens OX=9606 GN=IGHV3-74 PE=3 SV=1 - [HV374_HUMAN]	15.38	5	1	2	29	38.33	15.38	2	15	37.23	15.38	2	14	117	12.8	8.66
P05106	Integrin beta-3 OS=Homo sapiens OX=9606 GN=ITGB3 PE=1 SV=2 - [ITB3_HUMAN]	15.36	1	7	7	14	12.65	6.35	4	6	24.37	12.56	5	8	788	87.0	5.24
P04899	Guanine nucleotide-binding protein G(i) subunit alpha-2 OS=Homo sapiens OX=9606 GN=GNAI2 PE=1 SV=3 - [GNAI2_HUI	15.21	2	3	3	6	0.00	4.23	1	1	10.02	15.21	3	5	355	40.4	5.54
P14625	Endoplasmin OS=Homo sapiens OX=9606 GN=HSP90B1 PE=1 SV=1 - [ENPL_HUMAN]	15.19	1	7	7	11		0.00			33.59	15.19	7	11	803	92.4	4.84
P53396	ATP-citrate synthase OS=Homo sapiens OX=9606 GN=ACLY PE=1 SV=3 - [ACLY_HUMAN]	15.17	1	11	11	18		0.00			49.03	15.17	11	18	1101	120.8	7.33
A0A0B4J1Y9	Immunoglobulin heavy variable 3-72 OS=Homo sapiens OX=9606 GN=IGHV3-72 PE=3 SV=1 - [HV372_HUMAN]	15.13	3	2	2	22	10.27	15.13	2	9	13.97	15.13	2	13	119	13.2	7.85
P00367	Glutamate dehydrogenase 1, mitochondrial OS=Homo sapiens OX=9606 GN=GLUD1 PE=1 SV=2 - [DHE3_HUMAN]	15.05	2	5	5	8					20.65	15.05	5	8	558	61.4	7.80
Q8WWA1	Transmembrane protein 40 OS=Homo sapiens OX=9606 GN=TMEM40 PE=1 SV=2 - [TMM40_HUMAN]	15.02	1	1	1	3	0.00	15.02	1	3		0.00			233	25.5	5.59
P0C7Q2	Age-related maculopathy susceptibility protein 2 OS=Homo sapiens OX=9606 GN=ARMS2 PE=1 SV=1 - [ARMS2_HUMAN]	14.95	1	1	1	1		0.00			1.78	14.95	1	1	107	11.4	8.92
O00391	Sulfhydryl oxidase 1 OS=Homo sapiens OX=9606 GN=QSOX1 PE=1 SV=3 - [QSOX1_HUMAN]	14.86	1	7	7	8		0.00			19.50	14.86	7	8	747	82.5	8.92
P42765	3-ketoacyl-CoA thiolase, mitochondrial OS=Homo sapiens OX=9606 GN=ACAA2 PE=1 SV=2 - [THIM_HUMAN]	14.86	1	3	3	5		0.00			14.02	14.86	3	5	397	41.9	8.09
Q96KP4	Cytosolic non-specific dipeptidase OS=Homo sapiens OX=9606 GN=CNDP2 PE=1 SV=2 - [CNDP2_HUMAN]	14.74	1	4	4	7		0.00			19.72	14.74	4	7	475	52.8	5.97
P61009	Signal peptidase complex subunit 3 OS=Homo sapiens OX=9606 GN=SPCS3 PE=1 SV=1 - [SPCS3_HUMAN]	14.44	1	1	1	1	3.72	14.44	1	1					180	20.3	8.62
Q9H4B7	Tubulin beta-1 chain OS=Homo sapiens OX=9606 GN=TUBB1 PE=1 SV=1 - [TBB1_HUMAN]	14.41	1	4	4	5	5.32	8.43	2	2	9.66	5.99	2	3	451	50.3	5.17
P15169	Carboxypeptidase N catalytic chain OS=Homo sapiens OX=9606 GN=CPN1 PE=1 SV=1 - [CBPN_HUMAN]	14.19	1	3	3	7	13.57	14.19	3	4	8.42	14.19	3	3	458	52.3	7.34
P55056	Apolipoprotein C-IV OS=Homo sapiens OX=9606 GN=APOC4 PE=1 SV=1 - [APOC4_HUMAN]	14.17	1	2	2	11	16.48	14.17	2	11					127	14.5	8.92
P05155	Plasma protease C1 inhibitor OS=Homo sapiens OX=9606 GN=SERPING1 PE=1 SV=2 - [IC1_HUMAN]	14.00	1	5	5	17	21.66	8.20	3	9	26.99	14.00	5	8	500	55.1	6.55
Q8IZ83	Aldehyde dehydrogenase family 16 member A1 OS=Homo sapiens OX=9606 GN=ALDH16A1 PE=1 SV=2 - [A16A1_HUMAN]	13.97	1	6	6	15		0.00			42.96	13.97	6	15	802	85.1	6.79
P13671	Complement component C6 OS=Homo sapiens OX=9606 GN=C6 PE=1 SV=3 - [CO6_HUMAN]	13.70	1	7	7	10	22.84	13.70	7	9	2.06	1.39	1	1	934	104.7	6.76
P01593	Immunoglobulin kappa variable 1D-33 OS=Homo sapiens OX=9606 GN=IGKV1D-33 PE=1 SV=2 - [KVD33_HUMAN]	13.68	1	1	1	7	4.32	13.68	1	1	20.72	13.68	1	6	117	12.8	4.78
A0A0C4DH34	Immunoglobulin heavy variable 4-28 OS=Homo sapiens OX=9606 GN=IGHV4-28 PE=3 SV=1 - [HV428_HUMAN]	13.68	1	1	1	2	1.93	13.68	1	1	2.15	13.68	1	1	117	13.1	9.29
A0A0B4J2D9	Immunoglobulin kappa variable 1D-13 OS=Homo sapiens OX=9606 GN=IGKV1D-13 PE=3 SV=1 - [KVD13_HUMAN]	13.68	1	1	1	4	5.31	13.68	1	2	7.16	13.68	1	2	117	12.6	7.84
P02654	Apolipoprotein C-I OS=Homo sapiens OX=9606 GN=APOC1 PE=1 SV=1 - [APOC1_HUMAN]	13.25	1	1	1	2	5.43	13.25	1	2		0.00			83	9.3	8.47
Q5TEC6	Histone HIST2H3PS2 OS=Homo sapiens OX=9606 GN=H3-2 PE=1 SV=1 - [H3PS2_HUMAN]	13.24	2	1	2	18		0.00			33.10	13.24	2	18	136	15.4	11.27
P07996	Thrombospondin-1 OS=Homo sapiens OX=9606 GN=THBS1 PE=1 SV=2 - [TSP1_HUMAN]	13.08	1	10	10	17	3.06	1.54	1	1	43.94	13.08	10	16	1170	129.3	4.94
P05156	Complement factor I OS=Homo sapiens OX=9606 GN=CFI PE=1 SV=2 - [CFAI_HUMAN]	13.04	1	5	5	11	18.83	13.04	5	8	6.25	8.06	3	3	583	65.7	7.50
P51884	Lumican OS=Homo sapiens OX=9606 GN=LUM PE=1 SV=2 - [LUM_HUMAN]	13.02	1	3	3	5	3.49	4.44	1	2	6.72	13.02	3	3	338	38.4	6.61
P51946	Cyclin-H OS=Homo sapiens OX=9606 GN=CCNH PE=1 SV=1 - [CCNH_HUMAN]	13.00	1	1	1	1		0.00			3.68	13.00	1	1	323	37.6	7.15
P01599	Immunoglobulin kappa variable 1-17 OS=Homo sapiens OX=9606 GN=IGKV1-17 PE=1 SV=2 - [KV117_HUMAN]	12.82	1	1	1	3	1.86	12.82	1	1	6.40	12.82	1	2	117	12.8	8.68
P04424	Argininosuccinate lyase OS=Homo sapiens OX=9606 GN=ASL PE=1 SV=4 - [ARLY_HUMAN]	12.72	1	4	4	8		0.00			23.23	12.72	4	8	464	51.6	6.48
Q96B77	Transmembrane protein 186 OS=Homo sapiens OX=9606 GN=TMEM186 PE=1 SV=1 - [TM186_HUMAN]	12.68	1	1	1	1		0.00			2.67	12.68	1	1	213	24.9	9.86
P62879	Guanine nucleotide-binding protein G(I)/G(S)/G(T) subunit beta-2 OS=Homo sapiens OX=9606 GN=GNB2 PE=1 SV=3 - [GI	12.65	3	3	3	4		0.00			9.37	12.65	3	4	340	37.3	6.00
Q96QH8	Sperm acrosome-associated protein 5 OS=Homo sapiens OX=9606 GN=SPACA5 PE=2 SV=1 - [LYZL5_HUMAN]	12.58	1	1	1	1		0.00			2.54	12.58	1	1	159	17.9	6.42
O75352	Mannose-P-dolichol utilization defect 1 protein OS=Homo sapiens OX=9606 GN=MPDU1 PE=1 SV=2 - [MPU1_HUMAN]	12.55	1	1	1	1	3.79	12.55	1	1		0.00			247	26.6	8.94
P08514	Integrin alpha-IIb OS=Homo sapiens OX=9606 GN=ITGA2B PE=1 SV=3 - [ITA2B_HUMAN]	12.51	1	9	9	18	7.23	2.79	2	4	29.50	12.51	9	14	1039	113.3	5.38
Q8NGI1	Putative olfactory receptor 56B2 OS=Homo sapiens OX=9606 GN=OR56B2P PE=5 SV=1 - [O56B2_HUMAN]	12.42	1	1	1	1		0.00			0.00	12.42	1	1	322	35.9	8.70
P01889	HLA class I histocompatibility antigen, B alpha chain OS=Homo sapiens OX=9606 GN=HLA-B PE=1 SV=3 - [HLAB_HUMAN]	12.15	2	3	3	7	2.25	3.87	1	1	18.25	8.29	2	6	362	40.4	5.85
O14907	Tax1-binding protein 3 OS=Homo sapiens OX=9606 GN=TAX1BP3 PE=1 SV=2 - [TX1B3_HUMAN]	12.10	1	1	1	2		0.00			2.26	12.10	1	2	124	13.7	8.48
Q96KR6	Protein FAM210B, mitochondrial OS=Homo sapiens OX=9606 GN=FAM210B PE=1 SV=2 - [F210B_HUMAN]	11.98	1	1	1	2		0.00			6.14	11.98	1	2	192	20.4	10.43
Q5JXX7	Transmembrane protein 31 OS=Homo sapiens OX=9606 GN=TMEM31 PE=1 SV=1 - [TMM31_HUMAN]	11.90	1	1	1	1	2.97	11.90	1	1		0.00			168	19.8	6.54
P18085	ADP-ribosylation factor 4 OS=Homo sapiens OX=9606 GN=ARF4 PE=1 SV=3 - [ARF4_HUMAN]	11.67	4	1	1	1		0.00			2.14	11.67	1	1	180	20.5	7.14
Q3LXA3	Triokinase/FMN cyclase OS=Homo sapiens OX=9606 GN=TKFC PE=1 SV=2 - [TKFC_HUMAN]	11.65	1	5	5	14		0.00			34.18	11.65	5	14	575	58.9	7.49
P30043	Flavin reductase (NADPH) OS=Homo sapiens OX=9606 GN=BLVRB PE=1 SV=3 - [BLVRB_HUMAN]	11.65	1	1	1	1		0.00			2.92	11.65	1	1	206	22.1	7.65
P01034	Cystatin-C OS=Homo sapiens OX=9606 GN=CST3 PE=1 SV=1 - [CYTC_HUMAN]	11.64	1	1	1	1		0.00			2.25	11.64	1	1	146	15.8	8.75
P43652	Afamin OS=Homo sapiens OX=9606 GN=AFM PE=1 SV=1 - [AFAM_HUMAN]	11.52	1	4	4	12	7.96	5.01	2	3	23.10	11.52	4	9	599	69.0	5.90
Q7Z4W1	L-xylulose reductase OS=Homo sapiens OX=9606 GN=DCXR PE=1 SV=2 - [DCXR_HUMAN]	11.48	1	2	2	3		0.00			6.94	11.48	2	3	244	25.9	8.10
Q9NYW5	Taste receptor type 2 member 4 OS=Homo sapiens OX=9606 GN=TAS2R4 PE=1 SV=1 - [TA2R4_HUMAN]	11.37	1	1	1	1		0.00			3.72	11.37	1	1	299	33.8	9.82
Q6JVE6	Epididymal-specific lipocalin-10 OS=Homo sapiens OX=9606 GN=LCN10 PE=1 SV=1 - [LCN10_HUMAN]	11.23	1	1	1	1	3.10	11.23	1	1		0.00			187	20.7	10.35
Q16378	Proline-rich protein 4 OS=Homo sapiens OX=9606 GN=PRR4 PE=1 SV=3 - [PROL4_HUMAN]	11.19	1	1	1	4	8.16	11.19	1	2	7.00	11.19	1	2	134	15.1	7.06
P01700	Immunoglobulin lambda variable 1-47 OS=Homo sapiens OX=9606 GN=IGLV1-47 PE=1 SV=2 - [LV147_HUMAN]	11.11	1	1	1	6		0.00			13.68	11.11	1	6	117	12.3	5.91
Q86UX7	Fermitin family homolog 3 OS=Homo sapiens OX=9606 GN=FERMT3 PE=1 SV=1 - [URP2_HUMAN]	11.09	1	4	4	8	5.90	8.70	3	4	10.51	6.75	3	4	667	75.9	6.98
P35520	Cystathionine beta-synthase OS=Homo sapiens OX=9606 GN=CBS PE=1 SV=2 - [CBS_HUMAN]	11.07	1	3	3	5		0.00			11.26	11.07	3	5	551	60.5	6.65
Q96H72	Zinc transporter ZIP13 OS=Homo sapiens OX=9606 GN=SLC39A13 PE=1 SV=3 - [S39AD_HUMAN]	11.05	1	1	1	1	3.46	11.05	1	1		0.00			371	39.0	5.31
O43736	Integral membrane protein 2A OS=Homo sapiens OX=9606 GN=ITM2A PE=1 SV=2 - [ITM2A_HUMAN]	11.03	1	1	1	1	2.79	11.03	1	1		0.00			263	29.7	5.80
P07148	Fatty acid-binding protein, liver OS=Homo sapiens OX=9606 GN=FABP1 PE=1 SV=1 - [FABPL_HUMAN]	11.02	1	1	1	1		0.00			2.53	11.02	1	1	127	14.2	7.18
Q9BZM3	GS homeobox 2 OS=Homo sapiens OX=9606 GN=GSX2 PE=1 SV=2 - [GSX2_HUMAN]	10.86	1	1	1	1	2.69	10.86	1	1		0.00			304	32.0	8.97
P55082	Microfibril-associated glycoprotein 3 OS=Homo sapiens OX=9606 GN=MFAP3 PE=1 SV=1 - [MFAP3_HUMAN]	10.77	1	1	1	1		0.00			3.87	10.77	1	1	362	40.1	5.00
Q96KN8	Phospholipase A and acyltransferase 5 OS=Homo sapiens OX=9606 GN=PLAAT5 PE=1 SV=2 - [PLAT5_HUMAN]	10.75	1	1	1	1	3.58	10.75	1	1		0.00			279	30.3	8.24
P02788	Lactotransferrin OS=Homo sapiens OX=9606 GN=LTF PE=1 SV=6 - [TRFL_HUMAN]	10.70	1	4	4	10	11.96	6.20	2	5	12.82	10.70	4	5	710	78.1	8.12
A0A0C4DH24	Immunoglobulin kappa variable 6-21 OS=Homo sapiens OX=9606 GN=IGKV6-21 PE=3 SV=1 - [KV621_HUMAN]	10.53	1	1	1	2		0.00			4.13	10.53	1	2	114	12.4	7.28
Q9NX47	E3 ubiquitin-protein ligase MARCHF5 OS=Homo sapiens OX=9606 GN=MARCHF5 PE=1 SV=1 - [MARH5_HUMAN]	10.43	1	1	1	1		0.00			0.00	10.43	1	1	278	31.2	8.70
P02743	Serum amyloid P-component OS=Homo sapiens OX=9606 GN=APCS PE=1 SV=2 - [SAMP_HUMAN]	10.31	1	2	2	3		0.00			5.39	10.31	2	3	223	25.4	6.54
Q8IXM6	Nurim OS=Homo sapiens OX=9606 GN=NRM PE=1 SV=1 - [NRM_HUMAN]	10.31	1	1	1	1		0.00			2.76	10.31	1	1	262	29.4	8.63
F8VTS6	Ret finger protein-like 4A-like protein 1 OS=Homo sapiens OX=9606 GN=RFPL4AL1 PE=2 SV=1 - [RFAL1_HUMAN]	10.10	1	1	1	3	6.81	10.10	1	2	2.93	10.10	1	1	287	32.2	7.50
Q9Y664	KICSTOR complex protein kaptin OS=Homo sapiens OX=9606 GN=KPTN PE=1 SV=2 - [KPTN_HUMAN]	10.09	1	2	2	2	2.97	10.09	2	2		0.00			436	48.0	5.26
Q86UQ8	Transcription factor NF-E4 OS=Homo sapiens OX=9606 GN=NFE4 PE=1 SV=1 - [NFE4_HUMAN]	10.06	1	1	1	1	0.00	10.06	1	1		0.00			179	19.0	9.32
Q9H1C3	Glycosyltransferase 8 domain-containing protein 2 OS=Homo sapiens OX=9606 GN=GLT8D2 PE=2 SV=1 - [GL8D2_HUMAN]	10.03	1	1	1	1		0.00			4.56	10.03	1	1	349	40.0	7.06
P07988	Pulmonary surfactant-associated protein B OS=Homo sapiens OX=9606 GN=SFTPB PE=1 SV=3 - [PSPB_HUMAN]	9.97	1	2	2	2		0.00			4.62	9.97	2	2	381	42.1	5.49
Q9NVD7	Alpha-parvin OS=Homo sapiens OX=9606 GN=PARVA PE=1 SV=1 - [PARVA_HUMAN]	9.95	1	1	1	1	2.84	9.95	1	1		0.00			372	42.2	5.95
O75367	Core histone macro-H2A.1 OS=Homo sapiens OX=9606 GN=MACROH2A1 PE=1 SV=4 - [H2AY_HUMAN]	9.95	2	2	2	6		0.00			17.97	9.95	2	6	372	39.6	9.79
P55735	Protein SEC13 homolog OS=Homo sapiens OX=9606 GN=SEC13 PE=1 SV=3 - [SEC13_HUMAN]	9.94	1	2	2	3		0.00			8.57	9.94	2	3	322	35.5	5.48
P08238	Heat shock protein HSP 90-beta OS=Homo sapiens OX=9606 GN=HSP90AB1 PE=1 SV=4 - [HS90B_HUMAN]	9.81	6	2	5	11		0.00			30.29	9.81	5	11	724	83.2	5.03
Q9NV31	U3 small nucleolar ribonucleoprotein protein IMP3 OS=Homo sapiens OX=9606 GN=IMP3 PE=1 SV=1 - [IMP3_HUMAN]	9.78	1	1	1	1		0.00			3.70	9.78	1	1	184	21.8	9.50
P55084	Trifunctional enzyme subunit beta, mitochondrial OS=Homo sapiens OX=9606 GN=HADHB PE=1 SV=3 - [ECHB_HUMAN]	9.70	1	1	1	1		0.00			3.20	9.70	1	1	474	51.3	9.41
Q8NGT5	Olfactory receptor 9A2 OS=Homo sapiens OX=9606 GN=OR9A2 PE=2 SV=1 - [OR9A2_HUMAN]	9.68	1	1	1	1	3.38	9.68	1	1		0.00			310	35.3	8.51
P29966	Myristoylated alanine-rich C-kinase substrate OS=Homo sapiens OX=9606 GN=MARCKS PE=1 SV=4 - [MARCS_HUMAN]	9.64	1	1	1	2		0.00			6.79	9.64	1	2	332	31.5	4.45
Q9BTP6	Zinc finger BED domain-containing protein 2 OS=Homo sapiens OX=9606 GN=ZBED2 PE=1 SV=2 - [ZBED2_HUMAN]	9.63	1	1	1	1					3.99	9.63	1	1	218	25.1	7.06
Q8NGK0	Olfactory receptor 51G2 OS=Homo sapiens OX=9606 GN=OR51G2 PE=2 SV=1 - [O51G2_HUMAN]	9.55	1	1	1	1		0.00			3.53	9.55	1	1	314	35.0	8.59
P34896	Serine hydroxymethyltransferase, cytosolic OS=Homo sapiens OX=9606 GN=SHMT1 PE=1 SV=1 - [GLYC_HUMAN]	9.52	1	3	3	4		0.00			7.06	9.52	3	4	483	53.0	7.71
A0A0C4DH36	Probable non-functional immunoglobulin heavy variable 3-38 OS=Homo sapiens OX=9606 GN=IGHV3-38 PE=1 SV=1 - [HV:	9.48	1	1	1	1		0.00			2.53	9.48	1	1	116	12.8	9.25
P01764	Immunoglobulin heavy variable 3-23 OS=Homo sapiens OX=9606 GN=IGHV3-23 PE=1 SV=2 - [HV323_HUMAN]	9.40	2	1	1	6	17.96	9.40	1	6		0.00			117	12.6	8.28
A0A0C4DH35	Probable non-functional immunoglobulin heavy variable 3-35 OS=Homo sapiens OX=9606 GN=IGHV3-35 PE=1 SV=1 - [HV:	9.40	1	1	1	4	7.72	9.40	1	3	2.34	9.40	1	1	117	12.8	7.88
A0A0J9YX35	Immunoglobulin heavy variable 3-64D OS=Homo sapiens OX=9606 GN=IGHV3-64D PE=3 SV=1 - [HV64D_HUMAN]	9.40	1	1	1	1	2.33	9.40	1	1		0.00			117	12.8	7.85
Q8NGL6	Olfactory receptor 4A15 OS=Homo sapiens OX=9606 GN=OR4A15 PE=3 SV=3 - [O4A15_HUMAN]	9.30	1	1	1	1		0.00			3.13	9.30	1	1	344	38.8	8.76
P16157	Ankyrin-1 OS=Homo sapiens OX=9606 GN=ANK1 PE=1 SV=3 - [ANK1_HUMAN]	9.25	1	12	12	21		0.00			50.04	9.25	12	21	1881	206.1	6.01
Q9UJJ9	N-acetylglucosamine-1-phosphotransferase subunit gamma OS=Homo sapiens OX=9606 GN=GNPTG PE=1 SV=1 - [GNPTG_	9.18	1	1	1	1	4.47	9.18	1	1		0.00			305	34.0	6.95
P16671	Platelet glycoprotein 4 OS=Homo sapiens OX=9606 GN=CD36 PE=1 SV=2 - [CD36_HUMAN]	9.11	1	3	3	6	2.47	2.75	1	1	13.56	9.11	3	5	472	53.0	7.96
P12272	Parathyroid hormone-related protein OS=Homo sapiens OX=9606 GN=PTHLH PE=1 SV=1 - [PTHR_HUMAN]	9.04	1	1	1	1	2.62	9.04	1	1		0.00			177	20.2	10.18
Q96PS1	FANCD2 opposite strand protein OS=Homo sapiens OX=9606 GN=FANCD2OS PE=2 SV=1 - [FACOS_HUMAN]	9.04	1	1	1	1		0.00			2.88	9.04	1	1	177	20.2	9.33
O00559	Receptor-binding cancer antigen expressed on SiSo cells OS=Homo sapiens OX=9606 GN=EBAG9 PE=1 SV=1 - [RCAS1_HU	8.92	1	1	1	2	2.69	8.92	1	2		0.00			213	24.4	6.29
P60953	Cell division control protein 42 homolog OS=Homo sapiens OX=9606 GN=CDC42 PE=1 SV=2 - [CDC42_HUMAN]	8.90	1	1	1	2		0.00			5.97	8.90	1	2	191	21.2	6.55
P09467	Fructose-1,6-bisphosphatase 1 OS=Homo sapiens OX=9606 GN=FBP1 PE=1 SV=5 - [F16P1_HUMAN]	8.88	1	2	2	3		0.00			8.74	8.88	2	3	338	36.8	6.99
Q9Y6R7	IgGFc-binding protein OS=Homo sapiens OX=9606 GN=FCGBP PE=1 SV=3 - [FCGBP_HUMAN]	8.84	1	14	14	21		0.00			56.95	8.84	14	21	5405	571.6	5.34
Q6UX06	Olfactomedin-4 OS=Homo sapiens OX=9606 GN=OLFM4 PE=1 SV=1 - [OLFM4_HUMAN]	8.82	1	3	3	5		0.00			12.26	8.82	3	5	510	57.2	5.69
P02549	Spectrin alpha chain, erythrocytic 1 OS=Homo sapiens OX=9606 GN=SPTA1 PE=1 SV=5 - [SPTA1_HUMAN]	8.81	1	14	14	18		0.00			46.06	8.81	14	18	2419	279.8	5.05
P00748	Coagulation factor XII OS=Homo sapiens OX=9606 GN=F12 PE=1 SV=3 - [FA12_HUMAN]	8.78	1	3	3	6	8.92	6.18	2	3	7.45	5.53	2	3	615	67.7	7.74
Q9Y2Z9	Ubiquinone biosynthesis monooxygenase COQ6, mitochondrial OS=Homo sapiens OX=9606 GN=COQ6 PE=1 SV=2 - [COQ6	8.76	1	1	1	1	2.63	8.76	1	1		0.00			468	50.8	7.30
Q9UF02	Voltage-dependent calcium channel gamma-5 subunit OS=Homo sapiens OX=9606 GN=CACNG5 PE=2 SV=2 - [CCG5_HUM	8.73	1	1	1	6	0.00	8.73	1	6		0.00			275	30.9	7.33
P21810	Biglycan OS=Homo sapiens OX=9606 GN=BGN PE=1 SV=2 - [PGS1_HUMAN]	8.70	1	2	2	2		0.00			3.07	8.70	2	2	368	41.6	7.52
Q9Y536	Peptidyl-prolyl cis-trans isomerase A-like 4A OS=Homo sapiens OX=9606 GN=PPIAL4A PE=2 SV=1 - [PAL4A_HUMAN]	8.54	2	1	1	2	4.58	8.54	1	2		0.00			164	18.2	9.22
Q96DC7	Transmembrane and coiled-coil domain-containing protein 6 OS=Homo sapiens OX=9606 GN=TMCO6 PE=1 SV=2 - [TMCO	8.52	1	1	1	1		0.00			0.00	8.52	1	1	493	54.4	5.81

Q14654	ATP-sensitive inward rectifier potassium channel 11 OS=Homo sapiens OX=9606 GN=KCNJ11 PE=1 SV=2 - [KCJ11_HUMAN	8.46	1	1	1	1		0.00			3.95	8.46	1	1	390	43.5	8.00
P49908	Selenoprotein P OS=Homo sapiens OX=9606 GN=SELENOP PE=1 SV=3 - [SEPP1_HUMAN]	8.40	1	2	2	2	5.30	8.40	2	2		0.00			381	43.2	7.87
P05164	Myeloperoxidase OS=Homo sapiens OX=9606 GN=MPO PE=1 SV=1 - [PERM_HUMAN]	8.32	1	4	4	5		0.00			11.15	8.32	4	5	745	83.8	8.97
P08567	Pleckstrin OS=Homo sapiens OX=9606 GN=PLEK PE=1 SV=3 - [PLEK_HUMAN]	8.29	1	2	2	2	5.82	8.29	2	2		0.00			350	40.1	8.28
Q9UBQ7	Glyoxylate reductase/hydroxypyruvate reductase OS=Homo sapiens OX=9606 GN=GRHPR PE=1 SV=1 - [GRHPR_HUMAN]	8.23	1	2	2	3		0.00			7.34	8.23	2	3	328	35.6	7.39
Q14154	DAP3-binding cell death enhancer 1 OS=Homo sapiens OX=9606 GN=DELE1 PE=1 SV=3 - [DELE1_HUMAN]	8.16	1	1	1	1		0.00			3.59	8.16	1	1	515	55.9	9.25
Q8IY45	Protein AMN1 homolog OS=Homo sapiens OX=9606 GN=AMN1 PE=2 SV=4 - [AMN1_HUMAN]	8.14	1	1	1	1		0.00			3.12	8.14	1	1	258	28.4	7.85
P82251	b(0,+)-type amino acid transporter 1 OS=Homo sapiens OX=9606 GN=SLC7A9 PE=1 SV=1 - [BAT1_HUMAN]	8.01	1	1	1	1	2.52	8.01	1	1		0.00			487	53.4	8.12
P52797	Ephrin-A3 OS=Homo sapiens OX=9606 GN=EFNA3 PE=1 SV=1 - [EFNA3_HUMAN]	7.98	1	1	1	1		0.00			3.11	7.98	1	1	238	26.3	8.62
P00488	Coagulation factor XIII A chain OS=Homo sapiens OX=9606 GN=F13A1 PE=1 SV=4 - [F13A_HUMAN]	7.92	1	3	3	7	14.73	7.92	3	6	2.05	2.32	1	1	732	83.2	6.09
Q5D1E8	Endoribonuclease ZC3H12A OS=Homo sapiens OX=9606 GN=ZC3H12A PE=1 SV=1 - [ZC12A_HUMAN]	7.85	1	1	1	1	4.62	7.85	1	1		0.00			599	65.7	6.93
P11166	Solute carrier family 2, facilitated glucose transporter member 1 OS=Homo sapiens OX=9606 GN=SLC2A1 PE=1 SV=2 - [GT	7.72	1	2	2	5		0.00			12.81	7.72	2	5	492	54.0	8.72
Q70UQ0	Inhibitor of nuclear factor kappa-B kinase-interacting protein OS=Homo sapiens OX=9606 GN=IKBIP PE=1 SV=1 - [IKIP_H	7.71	1	1	1	1	0.00	7.71	1	1		0.00			350	39.3	9.17
O94889	Kelch-like protein 18 OS=Homo sapiens OX=9606 GN=KLHL18 PE=1 SV=3 - [KLH18_HUMAN]	7.67	1	1	1	1		0.00			4.45	7.67	1	1	574	63.6	5.60
A6NIZ1	Ras-related protein Rap-1b-like protein OS=Homo sapiens OX=9606 PE=2 SV=1 - [RP1BL_HUMAN]	7.61	3	2	2	6	5.29	6.52	1	2	11.98	7.61	2	4	184	20.9	5.48
P80404	4-aminobutyrate aminotransferase, mitochondrial OS=Homo sapiens OX=9606 GN=ABAT PE=1 SV=3 - [GABT_HUMAN]	7.60	1	2	2	2		0.00			5.38	7.60	2	2	500	56.4	7.96
A0A0A0MS15	Immunoglobulin heavy variable 3-49 OS=Homo sapiens OX=9606 GN=IGHV3-49 PE=3 SV=1 - [HV349_HUMAN]	7.56	1	1	1	6	3.74	7.56	1	2	8.19	7.56	1	4	119	13.0	8.62
Q9H0F6	Sharpin OS=Homo sapiens OX=9606 GN=SHARPIN PE=1 SV=1 - [SHRPN_HUMAN]	7.49	1	1	1	1	2.39	7.49	1	1					387	39.9	5.80
Q96FV0	Leucine-rich repeat-containing protein 46 OS=Homo sapiens OX=9606 GN=LRRC46 PE=2 SV=1 - [LRC46_HUMAN]	7.48	1	1	1	1	3.29	7.48	1	1		0.00			321	35.3	4.92
Q8NH41	Olfactory receptor 4K15 OS=Homo sapiens OX=9606 GN=OR4K15 PE=2 SV=2 - [OR4KF_HUMAN]	7.47	1	1	1	1	2.86	7.47	1	1		0.00			348	39.1	8.87
Q2M2I5	Keratin, type I cytoskeletal 24 OS=Homo sapiens OX=9606 GN=KRT24 PE=1 SV=1 - [K1C24_HUMAN]	7.43	1	1	2	2	3.21	5.33	1	1	2.22	2.10	1	1	525	55.1	4.96
Q9H867	Protein-lysine methyltransferase METTL21D OS=Homo sapiens OX=9606 GN=VCPKMT PE=1 SV=2 - [MT21D_HUMAN]	7.42	1	1	1	1		0.00			2.16	7.42	1	1	229	25.8	4.92
P51689	Arylsulfatase D OS=Homo sapiens OX=9606 GN=ARSD PE=1 SV=2 - [ARSD_HUMAN]	7.42	1	1	1	1		0.00			3.03	7.42	1	1	593	64.8	7.23
A0A075B6I0	Immunoglobulin lambda variable 8-61 OS=Homo sapiens OX=9606 GN=IGLV8-61 PE=3 SV=7 - [LV861_HUMAN]	7.38	1	1	1	2		0.00			3.64	7.38	1	2	122	12.8	4.55
Q2M3D2	Exocyst complex component 3-like protein 2 OS=Homo sapiens OX=9606 GN=EXOC3L2 PE=1 SV=1 - [EX3L2_HUMAN]	7.33	1	1	1	1	2.40	7.33	1	1		0.00			409	45.8	7.71
P15153	Ras-related C3 botulinum toxin substrate 2 OS=Homo sapiens OX=9606 GN=RAC2 PE=1 SV=1 - [RAC2_HUMAN]	7.29	1	1	1	1		0.00			2.26	7.29	1	1	192	21.4	7.61
P30084	Enoyl-CoA hydratase, mitochondrial OS=Homo sapiens OX=9606 GN=ECHS1 PE=1 SV=4 - [ECHM_HUMAN]	7.24	1	1	1	2		0.00			5.03	7.24	1	2	290	31.4	8.07
Q3LI59	Keratin-associated protein 21-2 OS=Homo sapiens OX=9606 GN=KRTAP21-2 PE=1 SV=2 - [KR212_HUMAN]	7.23	1	1	1	1	0.00	7.23	1	1		0.00			83	8.6	8.00
Q9H477	Ribokinase OS=Homo sapiens OX=9606 GN=RBKS PE=1 SV=1 - [RBSK_HUMAN]	7.14	1	1	1	1		0.00			3.00	7.14	1	1	322	34.1	5.05
P06732	Creatine kinase M-type OS=Homo sapiens OX=9606 GN=CKM PE=1 SV=2 - [KCRM_HUMAN]	7.09	1	1	1	1		0.00			3.17	7.09	1	1	381	43.1	7.25
O15446	DNA-directed RNA polymerase I subunit RPA34 OS=Homo sapiens OX=9606 GN=POLR1G PE=1 SV=1 - [RPA34_HUMAN]	7.06	1	1	1	1	4.54	7.06	1	1		0.00			510	55.0	8.51
O00624	Sodium-dependent phosphate transport protein 3 OS=Homo sapiens OX=9606 GN=SLC17A2 PE=2 SV=2 - [NPT3_HUMAN]	7.06	1	1	1	1	3.47	7.06	1	1		0.00			439	47.2	8.51
Q14493	Histone RNA hairpin-binding protein OS=Homo sapiens OX=9606 GN=SLBP PE=1 SV=1 - [SLBP_HUMAN]	7.04	1	1	1	1	0.00	7.04	1	1		0.00			270	31.3	7.47
P56199	Integrin alpha-1 OS=Homo sapiens OX=9606 GN=ITGA1 PE=1 SV=2 - [ITA1_HUMAN]	7.04	1	2	2	2	6.73	7.04	2	2		0.00			1179	130.8	6.29
P23381	Tryptophan--tRNA ligase, cytoplasmic OS=Homo sapiens OX=9606 GN=WARS1 PE=1 SV=2 - [SYWC_HUMAN]	7.01	1	2	2	2		0.00			5.19	7.01	2	2	471	53.1	6.23
Q9Y6K0	Choline/ethanolaminephosphotransferase 1 OS=Homo sapiens OX=9606 GN=CEPT1 PE=1 SV=1 - [CEPT1_HUMAN]	6.97	1	1	1	1	2.50	6.97	1	1		0.00			416	46.5	8.21
Q14CZ0	UPF0472 protein C16orf72 OS=Homo sapiens OX=9606 GN=C16orf72 PE=1 SV=1 - [CP072_HUMAN]	6.91	1	1	1	1	2.94	6.91	1	1		0.00			275	30.9	7.91
Q6DN72	Fc receptor-like protein 6 OS=Homo sapiens OX=9606 GN=FCRL6 PE=1 SV=2 - [FCRL6_HUMAN]	6.91	1	1	1	2		0.00			3.15	6.91	1	2	434	47.7	7.58
P10646	Tissue factor pathway inhibitor OS=Homo sapiens OX=9606 GN=TFPI PE=1 SV=1 - [TFPI1_HUMAN]	6.91	1	1	1	1		0.00			3.59	6.91	1	1	304	35.0	8.25
P14770	Platelet glycoprotein IX OS=Homo sapiens OX=9606 GN=GP9 PE=1 SV=3 - [GPIX_HUMAN]	6.78	1	1	1	1	2.65	6.78	1	1		0.00			177	19.0	6.34
P00480	Ornithine transcarbamylase, mitochondrial OS=Homo sapiens OX=9606 GN=OTC PE=1 SV=3 - [OTC_HUMAN]	6.78	1	2	2	2		0.00			4.22	6.78	2	2	354	39.9	8.63
Q8NGQ5	Olfactory receptor 9Q1 OS=Homo sapiens OX=9606 GN=OR9Q1 PE=2 SV=1 - [OR9Q1_HUMAN]	6.77	1	1	1	1		0.00			2.75	6.77	1	1	310	34.7	6.07
P49683	Prolactin-releasing peptide receptor OS=Homo sapiens OX=9606 GN=PRLHR PE=1 SV=3 - [PRLHR_HUMAN]	6.76	1	1	1	1	0.00	6.76	1	1		0.00			370	41.1	9.25
P26038	Moesin OS=Homo sapiens OX=9606 GN=MSN PE=1 SV=3 - [MOES_HUMAN]	6.76	3	3	3	7		0.00			13.63	6.76	3	7	577	67.8	6.40
A6NIJ9	Olfactory receptor 6C70 OS=Homo sapiens OX=9606 GN=OR6C70 PE=3 SV=1 - [O6C70_HUMAN]	6.73	1	1	1	2		0.00			0.00	6.73	1	2	312	35.4	8.92
Q9BYJ0	Fibroblast growth factor-binding protein 2 OS=Homo sapiens OX=9606 GN=FGFBP2 PE=1 SV=1 - [FGFP2_HUMAN]	6.73	1	1	1	1		0.00			0.00	6.73	1	1	223	24.6	8.87
Q99592	Zinc finger and BTB domain-containing protein 18 OS=Homo sapiens OX=9606 GN=ZBTB18 PE=1 SV=1 - [ZBT18_HUMAN]	6.70	1	2	2	2	2.08	6.70	2	2		0.00			522	58.3	5.69
P18206	Vinculin OS=Homo sapiens OX=9606 GN=VCL PE=1 SV=4 - [VINC_HUMAN]	6.70	1	4	4	5	2.39	1.32	1	1	10.75	5.38	3	4	1134	123.7	5.66
O60701	UDP-glucose 6-dehydrogenase OS=Homo sapiens OX=9606 GN=UGDH PE=1 SV=1 - [UGDH_HUMAN]	6.68	1	2	2	2		0.00			5.14	6.68	2	2	494	55.0	7.12
P24534	Elongation factor 1-beta OS=Homo sapiens OX=9606 GN=EEF1B2 PE=1 SV=3 - [EF1B_HUMAN]	6.67	1	1	1	1		0.00			2.22	6.67	1	1	225	24.7	4.67
O15524	Suppressor of cytokine signaling 1 OS=Homo sapiens OX=9606 GN=SOCS1 PE=1 SV=1 - [SOCS1_HUMAN]	6.64	1	1	1	1		0.00			2.37	6.64	1	1	211	23.5	10.98
Q9BWP8	Collectin-11 OS=Homo sapiens OX=9606 GN=COLEC11 PE=1 SV=1 - [COL11_HUMAN]	6.64	1	1	1	1	2.13	6.64	1	1		0.00			271	28.6	5.41
P22352	Glutathione peroxidase 3 OS=Homo sapiens OX=9606 GN=GPX3 PE=1 SV=2 - [GPX3_HUMAN]	6.64	1	1	1	3		0.00			6.44	6.64	1	3	226	25.5	8.13
O15321	Transmembrane 9 superfamily member 1 OS=Homo sapiens OX=9606 GN=TM9SF1 PE=2 SV=2 - [TM9S1_HUMAN]	6.60	1	1	1	1	3.49	6.60	1	1		0.00			606	68.8	7.17
Q15582	Transforming growth factor-beta-induced protein ig-h3 OS=Homo sapiens OX=9606 GN=TGFBI PE=1 SV=1 - [BGH3_HUMA	6.59	1	3	3	5		0.00			12.33	6.59	3	5	683	74.6	7.71
Q15084	Protein disulfide-isomerase A6 OS=Homo sapiens OX=9606 GN=PDIA6 PE=1 SV=1 - [PDIA6_HUMAN]	6.59	1	2	2	3		0.00			8.83	6.59	2	3	440	48.1	5.08
Q16698	2,4-dienoyl-CoA reductase [(3E)-enoyl-CoA-producing], mitochondrial OS=Homo sapiens OX=9606 GN=DECR1 PE=1 SV=1	6.57	1	1	1	1		0.00			2.21	6.57	1	1	335	36.0	9.28
Q16822	Phosphoenolpyruvate carboxykinase [GTP], mitochondrial OS=Homo sapiens OX=9606 GN=PCK2 PE=1 SV=4 - [PCKGM_HU	6.56	1	3	3	3		0.00			7.82	6.56	3	3	640	70.7	7.62
P59998	Actin-related protein 2/3 complex subunit 4 OS=Homo sapiens OX=9606 GN=ARPC4 PE=1 SV=3 - [ARPC4_HUMAN]	6.55	1	1	1	1		0.00			2.01	6.55	1	1	168	19.7	8.43
Q96AP0	Adrenocortical dysplasia protein homolog OS=Homo sapiens OX=9606 GN=ACD PE=1 SV=4 - [ACD_HUMAN]	6.55	1	1	1	1		0.00			3.43	6.55	1	1	458	48.9	5.30
Q8N5Y8	Protein mono-ADP-ribosyltransferase PARP16 OS=Homo sapiens OX=9606 GN=PARP16 PE=1 SV=2 - [PAR16_HUMAN]	6.52	1	1	1	1		0.00			3.06	6.52	1	1	322	36.4	9.06
Q8IVS2	Malonyl-CoA-acyl carrier protein transacylase, mitochondrial OS=Homo sapiens OX=9606 GN=MCAT PE=1 SV=2 - [FABD_H	6.41	1	1	1	1	2.49	6.41	1	1		0.00			390	42.9	8.72
Q6PKX4	Docking protein 6 OS=Homo sapiens OX=9606 GN=DOK6 PE=1 SV=1 - [DOK6_HUMAN]	6.34	1	2	2	2	4.51	6.34	2	2		0.00			331	38.3	8.48
P09972	Fructose-bisphosphate aldolase C OS=Homo sapiens OX=9606 GN=ALDOC PE=1 SV=2 - [ALDOC_HUMAN]	6.32	1	1	1	1		0.00			2.48	6.32	1	1	364	39.4	6.87
Q1W209	Embryonic stem cell-related gene protein OS=Homo sapiens OX=9606 GN=ESRG PE=2 SV=2 - [ESRG_HUMAN]	6.31	1	1	1	1		0.00			0.00	6.31	1	1	222	24.2	9.09
Q6Q788	Apolipoprotein A-V OS=Homo sapiens OX=9606 GN=APOA5 PE=1 SV=1 - [APOA5_HUMAN]	6.28	1	1	1	1		0.00			3.94	6.28	1	1	366	41.2	6.43
Q8N5S3	Uncharacterized protein C2orf73 OS=Homo sapiens OX=9606 GN=C2orf73 PE=2 SV=3 - [CB073_HUMAN]	6.27	1	1	1	1	2.34	6.27	1	1		0.00			287	32.1	8.97
Q9UMR3	T-box transcription factor TBX20 OS=Homo sapiens OX=9606 GN=TBX20 PE=1 SV=4 - [TBX20_HUMAN]	6.26	1	1	1	2	3.02	6.26	1	1	3.09	6.26	1	1	447	49.2	7.71
O75311	Glycine receptor subunit alpha-3 OS=Homo sapiens OX=9606 GN=GLRA3 PE=1 SV=2 - [GLRA3_HUMAN]	6.25	1	1	1	2		0.00			3.25	6.25	1	2	464	53.8	8.37
P16452	Protein 4.2 OS=Homo sapiens OX=9606 GN=EPB42 PE=1 SV=3 - [EPB42_HUMAN]	6.22	1	2	2	2		0.00			6.59	6.22	2	2	691	77.0	8.09
P19971	Thymidine phosphorylase OS=Homo sapiens OX=9606 GN=TYMP PE=1 SV=2 - [TYPH_HUMAN]	6.22	1	2	2	3		0.00			8.85	6.22	2	3	482	49.9	5.53
P31944	Caspase-14 OS=Homo sapiens OX=9606 GN=CASP14 PE=1 SV=2 - [CASPE_HUMAN]	6.20	1	1	1	1	2.03	6.20	1	1		0.00			242	27.7	5.58
Q6ZUI0	Tumor protein p63-regulated gene 1 protein OS=Homo sapiens OX=9606 GN=TPRG1 PE=1 SV=1 - [TPRG1_HUMAN]	6.18	1	1	1	1	2.23	6.18	1	1		0.00			275	31.2	7.09
Q96HR9	Receptor expression-enhancing protein 6 OS=Homo sapiens OX=9606 GN=REEP6 PE=1 SV=2 - [REEP6_HUMAN]	6.16	1	1	1	1		0.00			2.46	6.16	1	1	211	23.4	8.56
P48740	Mannan-binding lectin serine protease 1 OS=Homo sapiens OX=9606 GN=MASP1 PE=1 SV=3 - [MASP1_HUMAN]	6.15	1	3	3	4	9.85	6.15	3	4		0.00			699	79.2	5.49
Q8WV41	Sorting nexin-33 OS=Homo sapiens OX=9606 GN=SNX33 PE=1 SV=1 - [SNX33_HUMAN]	6.10	1	1	1	1	3.16	6.10	1	1		0.00			574	65.2	6.79
Q12946	Forkhead box protein F1 OS=Homo sapiens OX=9606 GN=FOXF1 PE=2 SV=2 - [FOXF1_HUMAN]	6.07	1	1	1	1		0.00			2.75	6.07	1	1	379	40.1	9.10
P67936	Tropomyosin alpha-4 chain OS=Homo sapiens OX=9606 GN=TPM4 PE=1 SV=3 - [TPM4_HUMAN]	6.05	1	1	1	1		0.00			3.24	6.05	1	1	248	28.5	4.69
P26022	Pentraxin-related protein PTX3 OS=Homo sapiens OX=9606 GN=PTX3 PE=1 SV=3 - [PTX3_HUMAN]	6.04	1	2	2	2		0.00			4.04	6.04	2	2	381	41.9	5.01
Q9UL15	BAG family molecular chaperone regulator 5 OS=Homo sapiens OX=9606 GN=BAG5 PE=1 SV=1 - [BAG5_HUMAN]	6.04	1	1	1	1		0.00			2.82	6.04	1	1	447	51.2	6.05
P0C7V7	Putative signal peptidase complex catalytic subunit SEC11B OS=Homo sapiens OX=9606 GN=SEC11B PE=5 SV=1 - [SC11B_	6.02	2	1	1	1		0.00			2.11	6.02	1	1	166	19.1	9.66
P07864	L-lactate dehydrogenase C chain OS=Homo sapiens OX=9606 GN=LDHC PE=1 SV=4 - [LDHC_HUMAN]	6.02	3	1	2	2		0.00			2.16	6.02	2	2	332	36.3	7.46
P00338	L-lactate dehydrogenase A chain OS=Homo sapiens OX=9606 GN=LDHA PE=1 SV=2 - [LDHA_HUMAN]	6.02	3	1	2	3		0.00			2.16	6.02	2	3	332	36.7	8.27
Q14943	Killer cell immunoglobulin-like receptor 3DS1 OS=Homo sapiens OX=9606 GN=KIR3DS1 PE=1 SV=2 - [KI3S1_HUMAN]	6.02	2	1	1	1	2.90	6.02	1	1		0.00			382	42.4	9.48
P05452	Tetranectin OS=Homo sapiens OX=9606 GN=CLEC3B PE=1 SV=3 - [TETN_HUMAN]	5.94	1	1	1	3	8.10	5.94	1	3		0.00			202	22.5	5.67
P41587	Vasoactive intestinal polypeptide receptor 2 OS=Homo sapiens OX=9606 GN=VIPR2 PE=1 SV=1 - [VIPR2_HUMAN]	5.94	1	1	1	1	3.90	5.94	1	1		0.00			438	49.4	7.94
Q6IMI6	Sulfotransferase 1C3 OS=Homo sapiens OX=9606 GN=SULT1C3 PE=1 SV=1 - [ST1C3_HUMAN]	5.92	1	1	1	1	2.65	5.92	1	1		0.00			304	35.9	6.92
Q9NVR2	Integrator complex subunit 10 OS=Homo sapiens OX=9606 GN=INTS10 PE=1 SV=2 - [INT10_HUMAN]	5.92	1	1	1	1	3.98	5.92	1	1		0.00			710	82.2	7.44
Q9Y261	Hepatocyte nuclear factor 3-beta OS=Homo sapiens OX=9606 GN=FOXA2 PE=1 SV=1 - [FOXA2_HUMAN]	5.91	1	1	1	1	3.29	5.91	1	1		0.00			457	48.3	8.69
Q5VUY0	Arylacetamide deacetylase-like 3 OS=Homo sapiens OX=9606 GN=AADACL3 PE=2 SV=5 - [ADCL3_HUMAN]	5.90	1	1	1	1	2.46	5.90	1	1		0.00			407	46.1	7.59
P01137	Transforming growth factor beta-1 proprotein OS=Homo sapiens OX=9606 GN=TGFB1 PE=1 SV=2 - [TGFB1_HUMAN]	5.90	1	1	1	1	2.39	5.90	1	1		0.00			390	44.3	8.53
A8MVZ5	Butyrophilin-like protein 10 OS=Homo sapiens OX=9606 GN=BTNL10 PE=5 SV=2 - [BTNLA_HUMAN]	5.84	1	1	1	1	2.30	5.84	1	1		0.00			291	32.6	8.34
Q99640	Membrane-associated tyrosine- and threonine-specific cdc2-inhibitory kinase OS=Homo sapiens OX=9606 GN=PKMYT1 PE=	5.81	1	1	1	1		0.00			2.92	5.81	1	1	499	54.5	6.93
P78417	Glutathione S-transferase omega-1 OS=Homo sapiens OX=9606 GN=GSTO1 PE=1 SV=2 - [GSTO1_HUMAN]	5.81	1	1	1	1		0.00			2.14	5.81	1	1	241	27.5	6.60
Q9GZZ8	Extracellular glycoprotein lacritin OS=Homo sapiens OX=9606 GN=LACRT PE=1 SV=1 - [LACRT_HUMAN]	5.80	1	1	1	3	5.17	5.80	1	3		0.00			138	14.2	5.50
Q5VZE5	N-alpha-acetyltransferase 35, NatC auxiliary subunit OS=Homo sapiens OX=9606 GN=NAA35 PE=1 SV=1 - [NAA35_HUMAN	5.79	1	1	1	1	4.47	5.79	1	1		0.00			725	83.6	7.05
O75828	Carbonyl reductase [NADPH] 3 OS=Homo sapiens OX=9606 GN=CBR3 PE=1 SV=3 - [CBR3_HUMAN]	5.78	2	1	1	1		0.00			2.84	5.78	1	1	277	30.8	6.18
O00401	Neural Wiskott-Aldrich syndrome protein OS=Homo sapiens OX=9606 GN=WASL PE=1 SV=2 - [WASL_HUMAN]	5.74	1	1	1	1		0.00			3.50	5.74	1	1	505	54.8	7.93
Q9UHG3	Prenylcysteine oxidase 1 OS=Homo sapiens OX=9606 GN=PCYOX1 PE=1 SV=3 - [PCYOX_HUMAN]	5.74	1	2	2	2		0.00			5.10	5.74	2	2	505	56.6	6.18
O95395	Beta-1,3-galactosyl-O-glycosyl-glycoprotein beta-1,6-N-acetylglucosaminyltransferase 3 OS=Homo sapiens OX=9606 GN=GC	5.71	1	1	1	1	3.94	5.71	1	1		0.00			438	50.8	8.25
P31937	3-hydroxyisobutyrate dehydrogenase, mitochondrial OS=Homo sapiens OX=9606 GN=HIBADH PE=1 SV=2 - [3HIDH_HUMA	5.65	1	1	1	1		0.00			1.66	5.65	1	1	336	35.3	8.13
Q9NSI2	Protein FAM207A OS=Homo sapiens OX=9606 GN=FAM207A PE=1 SV=2 - [F207A_HUMAN]	5.65	1	1	1	1		0.00			0.00	5.65	1	1	230	25.4	11.08
Q8IZ57	Neurensin-1 OS=Homo sapiens OX=9606 GN=NRSN1 PE=1 SV=1 - [NRSN1_HUMAN]	5.64	1	1	1	1		0.00			0.00	5.64	1	1	195	21.5	7.74
Q9Y5Z0	Beta-secretase 2 OS=Homo sapiens OX=9606 GN=BACE2 PE=1 SV=1 - [BACE2_HUMAN]	5.60	1	1	1	1		0.00			3.31	5.60	1	1	518	56.1	5.15
Q6P3X8	PiggyBac transposable element-derived protein 2 OS=Homo sapiens OX=9606 GN=PGBD2 PE=2 SV=1 - [PGBD2_HUMAN]	5.57	1	2	2	3		0.00			3.20	5.57	2	3	592	68.0	8.54
O00482	Nuclear receptor subfamily 5 group A member 2 OS=Homo sapiens OX=9606 GN=NR5A2 PE=1 SV=2 - [NR5A2_HUMAN]	5.55	1	1	1	2		0.00			4.28	5.55	1	2	541	61.3	7.88
Q3SX64	Outer dense fiber protein 3-like protein 2 OS=Homo sapiens OX=9606 GN=ODF3L2 PE=1 SV=2 - [OD3L2_HUMAN]	5.54	1	1	1	1	0.00	5.54	1	1		0.00			289	30.8	9.95
Q00534	Cyclin-dependent kinase 6 OS=Homo sapiens OX=9606 GN=CDK6 PE=1 SV=1 - [CDK6_HUMAN]	5.52	1	1	1	1	2.39	5.52	1	1		0.00			326	36.9	6.46
Q96KN2	Beta-Ala-His dipeptidase OS=Homo sapiens OX=9606 GN=CNDP1 PE=1 SV=4 - [CNDP1_HUMAN]	5.52	1	2	2	3	7.47	5.52	2	3		0.00			507	56.7	5.30
Q9Y2E6	E3 ubiquitin-protein ligase DTX4 OS=Homo sapiens OX=9606 GN=DTX4 PE=1 SV=2 - [DTX4_HUMAN]	5.49	1	1	1	1	2.90	5.49	1	1		0.00			619	67.2	9.60
P04075	Fructose-bisphosphate aldolase A OS=Homo sapiens OX=9606 GN=ALDOA PE=1 SV=2 - [ALDOA_HUMAN]	5.49	1	1	1	2		0.00			6.86	5.49	1	2	364	39.4	8.09
Q49AG3	Zinc finger BED domain-containing protein 5 OS=Homo sapiens OX=9606 GN=ZBED5 PE=2 SV=2 - [ZBED5_HUMAN]	5.48	1	2	2	2		0.00			5.45	5.48	2	2	693	78.9	8.27
Q96PM9	Zinc finger protein 385A OS=Homo sapiens OX=9606 GN=ZNF385A PE=1 SV=2 - [Z385A_HUMAN]	5.44	1	1	1	1		0.00			2.55	5.44	1	1	386	40.4	9.86
Q9H7B4	Histone-lysine N-methyltransferase SMYD3 OS=Homo sapiens OX=9606 GN=SMYD3 PE=1 SV=4 - [SMYD3_HUMAN]	5.37	1	1	1	1	2.72	5.37	1	1		0.00			428	49.1	7.25
A8MW95	Beclin-2 OS=Homo sapiens OX=9606 GN=BECN2 PE=1 SV=2 - [BECN2_HUMAN]	5.34	1	1	1	1		0.00			2.52	5.34	1	1	431	48.1	4.87
Q8NI99	Angiopoietin-related protein 6 OS=Homo sapiens OX=9606 GN=ANGPTL6 PE=1 SV=1 - [ANGL6_HUMAN]	5.32	1	1	1	1	0.00	5.32	1	1		0.00			470	51.7	8.53
Q13418	Integrin-linked protein kinase OS=Homo sapiens OX=9606 GN=ILK PE=1 SV=2 - [ILK_HUMAN]	5.31	1	2	2	2	4.21	5.31	2	2		0.00			452	51.4	8.07
P05543	Thyroxine-binding globulin OS=Homo sapiens OX=9606 GN=SERPINA7 PE=1 SV=2 - [THBG_HUMAN]	5.30	1	1	1	1	0.00	5.30	1	1		0.00			415	46.3	6.30
Q14392	Transforming growth factor beta activator LRRC32 OS=Homo sapiens OX=9606 GN=LRRC32 PE=1 SV=1 - [LRC32_HUMAN]	5.29	1	1	1	1		0.00			3.85	5.29	1	1	662	71.9	6.13
Q9NS39	Double-stranded RNA-specific editase B2 OS=Homo sapiens OX=9606 GN=ADARB2 PE=1 SV=1 - [RED2_HUMAN]	5.28	1	1	1	1		0.00			3.26	5.28	1	1	739	80.6	10.17
Q96K31	Uncharacterized protein C8orf76 OS=Homo sapiens OX=9606 GN=C8orf76 PE=2 SV=1 - [CH076_HUMAN]	5.26	1	1	1	1		0.00			2.63	5.26	1	1	380	43.2	5.43
Q7L1Q6	Basic leucine zipper and W2 domain-containing protein 1 OS=Homo sapiens OX=9606 GN=BZW1 PE=1 SV=1 - [BZW1_HUI	5.25	1	1	1	2	3.15	5.25	1	1	3.27	5.25	1	1	419	48.0	5.92
P52907	F-actin-capping protein subunit alpha-1 OS=Homo sapiens OX=9606 GN=CAPZA1 PE=1 SV=3 - [CAZA1_HUMAN]	5.24	1	1	1	1		0.00			2.42	5.24	1	1	286	32.9	5.69
P68104	Elongation factor 1-alpha 1 OS=Homo sapiens OX=9606 GN=EEF1A1 PE=1 SV=1 - [EF1A1_HUMAN]	5.19	2	1	1	2		0.00			2.94	5.19	1	2	462	50.1	9.01
Q7Z434	Mitochondrial antiviral-signaling protein OS=Homo sapiens OX=9606 GN=MAVS PE=1 SV=2 - [MAVS_HUMAN]	5.19	1	1	1	1		0.00			3.40	5.19	1	1	540	56.5	5.52
Q96D03	DNA damage-inducible transcript 4-like protein OS=Homo sapiens OX=9606 GN=DDIT4L PE=1 SV=1 - [DDT4L_HUMAN]	5.18	1	1	1	2		0.00			0.00	5.18	1	2	193	21.7	7.53
P01588	Erythropoietin OS=Homo sapiens OX=9606 GN=EPO PE=1 SV=1 - [EPO_HUMAN]	5.18	1	1	1	1		0.00			1.94	5.18	1	1	193	21.3	8.09
Q9H089	Large subunit GTPase 1 homolog OS=Homo sapiens OX=9606 GN=LSG1 PE=1 SV=2 - [LSG1_HUMAN]	5.17	1	1	1	1	4.04	5.17	1	1		0.00			658	75.2	6.38
Q6TFL4	Kelch-like protein 24 OS=Homo sapiens OX=9606 GN=KLHL24 PE=1 SV=1 - [KLH24_HUMAN]	5.17	2	2	2	2	2.56	4.17	1	1	1.42	1.00	1	1	600	68.3	6.39
Q8N1C3	Gamma-aminobutyric acid receptor subunit gamma-1 OS=Homo sapiens OX=9606 GN=GABRG1 PE=1 SV=2 - [GBRG1_HUI	5.16	2	1	1	1	3.36	5.16	1	1		0.00			465	53.6	8.13
Q9BW91	ADP-ribose pyrophosphatase, mitochondrial OS=Homo sapiens OX=9606 GN=NUDT9 PE=1 SV=1 - [NUDT9_HUMAN]	5.14	1	1	1	1		0.00			2.49	5.14	1	1	350	39.1	8.22
A8MV72	Putative UPF0607 protein ENSP00000382826 OS=Homo sapiens OX=9606 PE=5 SV=2 - [YH009_HUMAN]	5.14	4	1	1	1	2.08	5.14	1	1		0.00			311	34.1	10.58
P07237	Protein disulfide-isomerase OS=Homo sapiens OX=9606 GN=P4HB PE=1 SV=3 - [PDIA1_HUMAN]	5.12	1	2	2	2		0.00			5.36	5.12	2	2	508	57.1	4.87
Q9H2G2	STE20-like serine/threonine-protein kinase OS=Homo sapiens OX=9606 GN=SLK PE=1 SV=1 - [SLK_HUMAN]	5.10	1	2	2	2		0.00			6.08	5.10	2	2	1235	142.6	5.15
P08571	Monocyte differentiation antigen CD14 OS=Homo sapiens OX=9606 GN=CD14 PE=1 SV=2 - [CD14_HUMAN]	5.07	1	1	1	2		0.00			7.63	5.07	1	2	375	40.1	6.23
P51878	Caspase-5 OS=Homo sapiens OX=9606 GN=CASP5 PE=1 SV=3 - [CASP5_HUMAN]	5.07	1	1	1	1	2.85	5.07	1	1		0.00			434	49.7	9.16
Q9Y5W3	Krueppel-like factor 2 OS=Homo sapiens OX=9606 GN=KLF2 PE=1 SV=2 - [KLF2_HUMAN]	5.07	1	1	1	1	0.00	5.07	1	1		0.00			355	37.4	8.79
Q99496	E3 ubiquitin-protein ligase RING2 OS=Homo sapiens OX=9606 GN=RNF2 PE=1 SV=1 - [RING2_HUMAN]	5.06	1	1	1	1		0.00			0.00	5.06	1	1	336	37.6	6.84
Q8NB59	Synaptotagmin-14 OS=Homo sapiens OX=9606 GN=SYT14 PE=1 SV=2 - [SYT14_HUMAN]	5.05	1	1	1	1		0.00			2.47	5.05	1	1	555	62.2	6.44
Q99808	Equilibrative nucleoside transporter 1 OS=Homo sapiens OX=9606 GN=SLC29A1 PE=1 SV=3 - [S29A1_HUMAN]	5.04	1	1	1	1		0.00			3.10	5.04	1	1	456	50.2	8.29
Q8NHP6	Motile sperm domain-containing protein 2 OS=Homo sapiens OX=9606 GN=MOSPD2 PE=1 SV=1 - [MSPD2_HUMAN]	5.02	1	1	1	1	2.36	5.02	1	1		0.00			518	59.7	5.96
Q9NRM0	Solute carrier family 2, facilitated glucose transporter member 9 OS=Homo sapiens OX=9606 GN=SLC2A9 PE=1 SV=2 - [G	5.00	1	1	1	1	4.00	5.00	1	1		0.00			540	58.7	8.90
P00740	Coagulation factor IX OS=Homo sapiens OX=9606 GN=F9 PE=1 SV=2 - [FA9_HUMAN]	4.99	1	2	2	3	2.74	3.04	1	2	2.13	1.95	1	1	461	51.7	5.47
Q86YQ8	Copine-8 OS=Homo sapiens OX=9606 GN=CPNE8 PE=1 SV=2 - [CPNE8_HUMAN]	4.96	1	1	1	1	2.37	4.96	1	1		0.00			564	63.1	5.96
Q9UBR1	Beta-ureidopropionase OS=Homo sapiens OX=9606 GN=UPB1 PE=1 SV=1 - [BUP1_HUMAN]	4.95	1	1	1	1		0.00			2.51	4.95	1	1	384	43.1	6.54
P13798	Acylamino-acid-releasing enzyme OS=Homo sapiens OX=9606 GN=APEH PE=1 SV=4 - [ACPH_HUMAN]	4.92	1	2	2	2	0.00	3.42	1	1	1.70	1.50	1	1	732	81.2	5.48
P19235	Erythropoietin receptor OS=Homo sapiens OX=9606 GN=EPOR PE=1 SV=1 - [EPOR_HUMAN]	4.92	1	1	1	1	3.55	4.92	1	1		0.00			508	55.0	4.74
Q9UBB9	Tuftelin-interacting protein 11 OS=Homo sapiens OX=9606 GN=TFIP11 PE=1 SV=1 - [TFP11_HUMAN]	4.90	1	1	1	1		0.00			3.26	4.90	1	1	837	96.8	5.67
O95757	Heat shock 70 kDa protein 4L OS=Homo sapiens OX=9606 GN=HSPA4L PE=1 SV=3 - [HS74L_HUMAN]	4.89	1	1	1	2	4.22	4.89	1	2		0.00			839	94.5	5.88
Q9H7L9	Sin3 histone deacetylase corepressor complex component SDS3 OS=Homo sapiens OX=9606 GN=SUDS3 PE=1 SV=2 - [SDS	4.88	1	1	1	1	2.80	4.88	1	1		0.00			328	38.1	5.66
Q8ND61	Uncharacterized protein C3orf20 OS=Homo sapiens OX=9606 GN=C3orf20 PE=2 SV=2 - [CC020_HUMAN]	4.87	1	1	1	1	3.90	4.87	1	1		0.00			904	101.2	9.13
Q13948	Protein CASP OS=Homo sapiens OX=9606 GN=CUX1 PE=1 SV=2 - [CASP_HUMAN]	4.87	1	1	1	1		0.00			3.58	4.87	1	1	678	77.4	5.44
Q5JRV8	Transmembrane protein 255A OS=Homo sapiens OX=9606 GN=TMEM255A PE=2 SV=1 - [T255A_HUMAN]	4.87	1	1	1	1		0.00			1.68	4.87	1	1	349	38.4	7.99
Q01518	Adenylyl cyclase-associated protein 1 OS=Homo sapiens OX=9606 GN=CAP1 PE=1 SV=5 - [CAP1_HUMAN]	4.84	1	1	1	1	3.17	4.84	1	1		0.00			475	51.9	8.06
O94788	Retinal dehydrogenase 2 OS=Homo sapiens OX=9606 GN=ALDH1A2 PE=1 SV=3 - [AL1A2_HUMAN]	4.83	1	1	1	1		0.00			3.07	4.83	1	1	518	56.7	6.05
O95995	Dynein regulatory complex subunit 4 OS=Homo sapiens OX=9606 GN=GAS8 PE=1 SV=1 - [DRC4_HUMAN]	4.81	1	1	1	1		0.00			2.43	4.81	1	1	478	56.3	7.94
Q8NFA2	NADPH oxidase organizer 1 OS=Homo sapiens OX=9606 GN=NOXO1 PE=1 SV=1 - [NOXO1_HUMAN]	4.79	1	1	1	1		0.00			2.69	4.79	1	1	376	41.2	9.83
P54284	Voltage-dependent L-type calcium channel subunit beta-3 OS=Homo sapiens OX=9606 GN=CACNB3 PE=1 SV=1 - [CACB3_	4.75	1	1	1	1	2.61	4.75	1	1		0.00			484	54.5	6.35
P55895	V(D)J recombination-activating protein 2 OS=Homo sapiens OX=9606 GN=RAG2 PE=1 SV=1 - [RAG2_HUMAN]	4.74	1	1	1	1		0.00			2.94	4.74	1	1	527	59.2	5.87
Q03989	AT-rich interactive domain-containing protein 5A OS=Homo sapiens OX=9606 GN=ARID5A PE=1 SV=2 - [ARI5A_HUMAN]	4.71	1	1	1	1		0.00			3.20	4.71	1	1	594	64.0	9.25
Q02386	Zinc finger protein 45 OS=Homo sapiens OX=9606 GN=ZNF45 PE=1 SV=2 - [ZNF45_HUMAN]	4.69	1	1	1	1		0.00			4.49	4.69	1	1	682	78.2	8.66
O95201	Zinc finger protein 205 OS=Homo sapiens OX=9606 GN=ZNF205 PE=1 SV=2 - [ZN205_HUMAN]	4.69	1	1	1	1	2.46	4.69	1	1		0.00			554	60.6	8.54

Q96NU7	Probable imidazolonepropionase OS=Homo sapiens OX=9606 GN=AMDHD1 PE=1 SV=2 - [HUTI_HUMAN]	4.69	1	1	1	1		0.00			2.32	4.69	1	1	426	46.7	6.61
Q6IN97	Putative protein FRMPD2-like OS=Homo sapiens OX=9606 GN=FRMPD2B PE=5 SV=1 - [FRP2L_HUMAN]	4.69	2	1	1	1		0.00			0.00	4.69	1	1	320	35.1	6.24
Q93088	Betaine--homocysteine S-methyltransferase 1 OS=Homo sapiens OX=9606 GN=BHMT PE=1 SV=2 - [BHMT1_HUMAN]	4.68	1	1	1	1		0.00			3.43	4.68	1	1	406	45.0	7.03
Q15113	Procollagen C-endopeptidase enhancer 1 OS=Homo sapiens OX=9606 GN=PCOLCE PE=1 SV=2 - [PCOC1_HUMAN]	4.68	1	1	1	2	2.62	4.68	1	1	2.77	4.68	1	1	449	47.9	7.43
Q3V5L5	Alpha-1,6-mannosylglycoprotein 6-beta-N-acetylglucosaminyltransferase B OS=Homo sapiens OX=9606 GN=MGAT5B PE=1	4.67	1	1	1	1	3.13	4.67	1	1		0.00			792	89.5	8.35
P47974	mRNA decay activator protein ZFP36L2 OS=Homo sapiens OX=9606 GN=ZFP36L2 PE=1 SV=3 - [TISD_HUMAN]	4.66	1	1	1	1	2.75	4.66	1	1		0.00			494	51.0	8.16
Q14952	Killer cell immunoglobulin-like receptor 2DS3 OS=Homo sapiens OX=9606 GN=KIR2DS3 PE=2 SV=1 - [KI2S3_HUMAN]	4.61	2	1	1	1		0.00			0.00	4.61	1	1	304	33.7	6.58
P02750	Leucine-rich alpha-2-glycoprotein OS=Homo sapiens OX=9606 GN=LRG1 PE=1 SV=2 - [A2GL_HUMAN]	4.61	1	1	1	1		0.00			3.24	4.61	1	1	347	38.2	6.95
P15104	Glutamine synthetase OS=Homo sapiens OX=9606 GN=GLUL PE=1 SV=4 - [GLNA_HUMAN]	4.56	1	1	1	1		0.00			2.83	4.56	1	1	373	42.0	6.89
P11277	Spectrin beta chain, erythrocytic OS=Homo sapiens OX=9606 GN=SPTB PE=1 SV=5 - [SPTB1_HUMAN]	4.54	1	7	7	15		0.00			40.55	4.54	7	15	2137	246.3	5.27
Q9GZN7	Protein rogdi homolog OS=Homo sapiens OX=9606 GN=ROGDI PE=1 SV=1 - [ROGDI_HUMAN]	4.53	1	1	1	2	3.07	4.53	1	2		0.00			287	32.2	8.16
P85298	Rho GTPase-activating protein 8 OS=Homo sapiens OX=9606 GN=ARHGAP8 PE=1 SV=1 - [RHG08_HUMAN]	4.53	1	1	1	1		0.00			2.57	4.53	1	1	464	53.5	9.41
Q9BT22	Chitobiosyldiphosphodolichol beta-mannosyltransferase OS=Homo sapiens OX=9606 GN=ALG1 PE=1 SV=2 - [ALG1_HUMAN	4.53	1	1	1	1	3.06	4.53	1	1		0.00			464	52.5	7.23
O60260	E3 ubiquitin-protein ligase parkin OS=Homo sapiens OX=9606 GN=PRKN PE=1 SV=2 - [PRKN_HUMAN]	4.52	1	1	1	1	2.52	4.52	1	1		0.00			465	51.6	7.06
Q8NCW5	NAD(P)H-hydrate epimerase OS=Homo sapiens OX=9606 GN=NAXE PE=1 SV=2 - [NNRE_HUMAN]	4.51	1	1	1	1		0.00			2.09	4.51	1	1	288	31.7	7.66
Q9Y6Y8	SEC23-interacting protein OS=Homo sapiens OX=9606 GN=SEC23IP PE=1 SV=1 - [S23IP_HUMAN]	4.50	1	1	1	3	3.43	4.50	1	3		0.00			1000	111.0	5.54
Q9Y6L6	Solute carrier organic anion transporter family member 1B1 OS=Homo sapiens OX=9606 GN=SLCO1B1 PE=1 SV=2 - [SO1E	4.49	1	1	1	1	2.88	4.49	1	1		0.00			691	76.4	8.57
Q13561	Dynactin subunit 2 OS=Homo sapiens OX=9606 GN=DCTN2 PE=1 SV=4 - [DCTN2_HUMAN]	4.49	1	1	1	1		0.00			1.96	4.49	1	1	401	44.2	5.21
Q96IY4	Carboxypeptidase B2 OS=Homo sapiens OX=9606 GN=CPB2 PE=1 SV=2 - [CBPB2_HUMAN]	4.49	1	2	2	5	10.65	4.49	2	5		0.00			423	48.4	7.71
P11142	Heat shock cognate 71 kDa protein OS=Homo sapiens OX=9606 GN=HSPA8 PE=1 SV=1 - [HSP7C_HUMAN]	4.49	2	2	2	2		0.00			5.18	4.49	2	2	646	70.9	5.52
Q06323	Proteasome activator complex subunit 1 OS=Homo sapiens OX=9606 GN=PSME1 PE=1 SV=1 - [PSME1_HUMAN]	4.42	1	1	1	3		0.00			7.46	4.42	1	3	249	28.7	6.02
Q7Z6G3	N-terminal EF-hand calcium-binding protein 2 OS=Homo sapiens OX=9606 GN=NECAB2 PE=1 SV=1 - [NECA2_HUMAN]	4.40	1	1	1	1		0.00			0.00	4.40	1	1	386	43.2	5.50
P20810	Calpastatin OS=Homo sapiens OX=9606 GN=CAST PE=1 SV=4 - [ICAL_HUMAN]	4.38	1	1	1	2	2.45	4.38	1	2		0.00			708	76.5	5.07
Q9BVP2	Guanine nucleotide-binding protein-like 3 OS=Homo sapiens OX=9606 GN=GNL3 PE=1 SV=2 - [GNL3_HUMAN]	4.37	1	1	1	1	2.89	4.37	1	1		0.00			549	62.0	9.16
Q08AH1	Acyl-coenzyme A synthetase ACSM1, mitochondrial OS=Homo sapiens OX=9606 GN=ACSM1 PE=1 SV=1 - [ACSM1_HUMAN]	4.33	1	1	1	1		0.00			2.72	4.33	1	1	577	65.2	8.28
O95741	Copine-6 OS=Homo sapiens OX=9606 GN=CPNE6 PE=1 SV=3 - [CPNE6_HUMAN]	4.31	1	1	1	2	2.94	4.31	1	1	3.16	4.31	1	1	557	62.0	5.52
P01130	Low-density lipoprotein receptor OS=Homo sapiens OX=9606 GN=LDLR PE=1 SV=1 - [LDLR_HUMAN]	4.30	1	1	1	1		0.00			5.24	4.30	1	1	860	95.3	5.05
Q9Y5Z6	Beta-1,3-galactosyltransferase 1 OS=Homo sapiens OX=9606 GN=B3GALT1 PE=1 SV=1 - [B3GT1_HUMAN]	4.29	1	1	1	2	2.40	4.29	1	2		0.00			326	38.0	9.29
P56693	Transcription factor SOX-10 OS=Homo sapiens OX=9606 GN=SOX10 PE=1 SV=1 - [SOX10_HUMAN]	4.29	1	1	1	1		0.00			2.55	4.29	1	1	466	49.9	6.67
Q9NZV8	Potassium voltage-gated channel subfamily D member 2 OS=Homo sapiens OX=9606 GN=KCND2 PE=1 SV=2 - [KCND2_HU	4.29	3	1	1	1	3.19	4.29	1	1		0.00			630	70.5	7.97
Q92733	Proline-rich protein PRCC OS=Homo sapiens OX=9606 GN=PRCC PE=1 SV=1 - [PRCC_HUMAN]	4.28	1	1	1	2		0.00			2.85	4.28	1	2	491	52.4	5.10
Q8TCB0	Interferon-induced protein 44 OS=Homo sapiens OX=9606 GN=IFI44 PE=1 SV=2 - [IFI44_HUMAN]	4.28	1	1	1	1	0.00	4.28	1	1		0.00			444	50.5	6.89
A8MYJ7	Tetratricopeptide repeat protein 34 OS=Homo sapiens OX=9606 GN=TTC34 PE=2 SV=2 - [TTC34_HUMAN]	4.24	1	1	1	1	3.33	4.24	1	1		0.00			566	60.9	7.33
Q68CP4	Heparan-alpha-glucosaminide N-acetyltransferase OS=Homo sapiens OX=9606 GN=HGSNAT PE=1 SV=2 - [HGNAT_HUMAN	4.22	1	1	1	1		0.00			2.49	4.22	1	1	663	73.2	8.40
Q8TD90	Melanoma-associated antigen E2 OS=Homo sapiens OX=9606 GN=MAGEE2 PE=2 SV=1 - [MAGE2_HUMAN]	4.21	1	1	1	1		0.00			3.34	4.21	1	1	523	60.3	5.10
Q4W5G0	Tigger transposable element-derived protein 2 OS=Homo sapiens OX=9606 GN=TIGD2 PE=3 SV=1 - [TIGD2_HUMAN]	4.19	1	1	1	1		0.00			3.01	4.19	1	1	525	59.6	9.00
P01833	Polymeric immunoglobulin receptor OS=Homo sapiens OX=9606 GN=PIGR PE=1 SV=4 - [PIGR_HUMAN]	4.19	1	2	2	2		0.00			4.41	4.19	2	2	764	83.2	5.74
O76050	E3 ubiquitin-protein ligase NEURL1 OS=Homo sapiens OX=9606 GN=NEURL1 PE=2 SV=1 - [NEUL1_HUMAN]	4.18	1	1	1	1		0.00			3.05	4.18	1	1	574	61.8	8.51
Q8NDL9	Cytosolic carboxypeptidase-like protein 5 OS=Homo sapiens OX=9606 GN=AGBL5 PE=2 SV=1 - [CBPC5_HUMAN]	4.18	1	1	1	1	3.91	4.18	1	1		0.00			886	97.5	9.17
Q9UKD2	mRNA turnover protein 4 homolog OS=Homo sapiens OX=9606 GN=MRTO4 PE=1 SV=2 - [MRT4_HUMAN]	4.18	1	1	1	1		0.00			1.68	4.18	1	1	239	27.5	8.29
Q6PI73	Leukocyte immunoglobulin-like receptor subfamily A member 6 OS=Homo sapiens OX=9606 GN=LILRA6 PE=2 SV=2 - [LIR/	4.16	2	1	1	1	2.21	4.16	1	1		0.00			481	52.4	7.77
Q8N841	Tubulin polyglutamylase TTLL6 OS=Homo sapiens OX=9606 GN=TTLL6 PE=1 SV=2 - [TTLL6_HUMAN]	4.15	1	1	1	1	3.16	4.15	1	1		0.00			843	96.3	8.82
Q8NA31	Telomere repeats-binding bouquet formation protein 1 OS=Homo sapiens OX=9606 GN=TERB1 PE=1 SV=3 - [TERB1_HUM	4.13	1	1	1	1		0.00			3.57	4.13	1	1	727	83.0	7.46
P06681	Complement C2 OS=Homo sapiens OX=9606 GN=C2 PE=1 SV=2 - [CO2_HUMAN]	4.12	1	1	1	1		0.00			3.90	4.12	1	1	752	83.2	7.42
P21549	Serine--pyruvate aminotransferase OS=Homo sapiens OX=9606 GN=AGXT PE=1 SV=1 - [SPYA_HUMAN]	4.08	1	1	1	1		0.00			2.22	4.08	1	1	392	43.0	8.40
P00558	Phosphoglycerate kinase 1 OS=Homo sapiens OX=9606 GN=PGK1 PE=1 SV=3 - [PGK1_HUMAN]	4.08	1	1	1	1		0.00			2.18	4.08	1	1	417	44.6	8.10
Q9UF83	Uncharacterized protein DKFZp434B061 OS=Homo sapiens OX=9606 PE=2 SV=2 - [YM012_HUMAN]	4.08	1	1	1	1	2.60	4.08	1	1		0.00			564	59.4	13.07
P26439	3 beta-hydroxysteroid dehydrogenase/Delta 5-->4-isomerase type 2 OS=Homo sapiens OX=9606 GN=HSD3B2 PE=1 SV=2 -	4.03	1	1	1	1	2.53	4.03	1	1		0.00			372	42.0	8.03
Q7Z402	Transmembrane channel-like protein 7 OS=Homo sapiens OX=9606 GN=TMC7 PE=1 SV=1 - [TMC7_HUMAN]	4.01	1	1	1	1		0.00			0.00	4.01	1	1	723	83.4	8.43
Q9NRX2	39S ribosomal protein L17, mitochondrial OS=Homo sapiens OX=9606 GN=MRPL17 PE=1 SV=1 - [RM17_HUMAN]	4.00	1	1	1	1	1.91	4.00	1	1		0.00			175	20.0	10.11
Q13790	Apolipoprotein F OS=Homo sapiens OX=9606 GN=APOF PE=1 SV=2 - [APOF_HUMAN]	3.99	1	1	1	1		0.00			2.88	3.99	1	1	326	35.4	5.64
P0CW18	Serine protease 56 OS=Homo sapiens OX=9606 GN=PRSS56 PE=2 SV=1 - [PRS56_HUMAN]	3.98	1	1	1	1		0.00			3.72	3.98	1	1	603	64.6	8.76
Q8TBM8	DnaJ homolog subfamily B member 14 OS=Homo sapiens OX=9606 GN=DNAJB14 PE=1 SV=1 - [DJB14_HUMAN]	3.96	1	1	1	1		0.00			2.04	3.96	1	1	379	42.5	8.59
Q9BX69	Caspase recruitment domain-containing protein 6 OS=Homo sapiens OX=9606 GN=CARD6 PE=1 SV=2 - [CARD6_HUMAN]	3.95	1	2	2	3	2.94	2.41	1	2	2.11	1.54	1	1	1037	116.4	6.37
Q16186	Proteasomal ubiquitin receptor ADRM1 OS=Homo sapiens OX=9606 GN=ADRM1 PE=1 SV=2 - [ADRM1_HUMAN]	3.93	1	1	1	2		0.00			2.06	3.93	1	2	407	42.1	5.07
Q9BVQ7	Spermatogenesis-associated protein 5-like protein 1 OS=Homo sapiens OX=9606 GN=SPATA5L1 PE=1 SV=2 - [SPA5L_HUM	3.85	1	1	1	2		0.00			3.13	3.85	1	2	753	80.7	8.09
P78385	Keratin, type II cuticular Hb3 OS=Homo sapiens OX=9606 GN=KRT83 PE=1 SV=2 - [KRT83_HUMAN]	3.85	1	1	1	1		0.00			2.84	3.85	1	1	493	54.2	5.64
A0A087X1G2	TBC1 domain family member 3K OS=Homo sapiens OX=9606 GN=TBC1D3K PE=3 SV=1 - [TBC3K_HUMAN]	3.83	4	1	1	1		0.00			2.27	3.83	1	1	549	62.1	9.07
Q8WVV5	Butyrophilin subfamily 2 member A2 OS=Homo sapiens OX=9606 GN=BTN2A2 PE=1 SV=2 - [BT2A2_HUMAN]	3.82	1	1	1	1	2.23	3.82	1	1		0.00			523	59.0	6.01
Q8NGG3	Olfactory receptor 5T3 OS=Homo sapiens OX=9606 GN=OR5T3 PE=3 SV=3 - [OR5T3_HUMAN]	3.82	1	1	1	1		0.00			2.44	3.82	1	1	340	38.3	8.09
O15231	Zinc finger protein 185 OS=Homo sapiens OX=9606 GN=ZNF185 PE=1 SV=3 - [ZN185_HUMAN]	3.77	1	1	1	1		0.00			2.59	3.77	1	1	689	73.5	7.01
P08842	Steryl-sulfatase OS=Homo sapiens OX=9606 GN=STS PE=1 SV=2 - [STS_HUMAN]	3.77	1	1	1	1	2.37	3.77	1	1		0.00			583	65.5	7.66
Q8N883	Zinc finger protein 614 OS=Homo sapiens OX=9606 GN=ZNF614 PE=1 SV=2 - [ZN614_HUMAN]	3.76	1	1	1	1		0.00			2.14	3.76	1	1	585	67.2	9.04
Q96RP8	Potassium voltage-gated channel subfamily A member 7 OS=Homo sapiens OX=9606 GN=KCNA7 PE=2 SV=1 - [KCNA7_HU	3.73	1	1	1	1		0.00			1.77	3.73	1	1	456	50.5	7.34
O15164	Transcription intermediary factor 1-alpha OS=Homo sapiens OX=9606 GN=TRIM24 PE=1 SV=3 - [TIF1A_HUMAN]	3.71	1	1	1	1	3.67	3.71	1	1		0.00			1050	116.8	7.11
P33151	Cadherin-5 OS=Homo sapiens OX=9606 GN=CDH5 PE=1 SV=5 - [CADH5_HUMAN]	3.70	1	2	2	3		0.00			7.60	3.70	2	3	784	87.5	5.43
Q04695	Keratin, type I cytoskeletal 17 OS=Homo sapiens OX=9606 GN=KRT17 PE=1 SV=2 - [K1C17_HUMAN]	3.70	1	1	1	1		0.00			2.12	3.70	1	1	432	48.1	5.02
Q96CX2	BTB/POZ domain-containing protein KCTD12 OS=Homo sapiens OX=9606 GN=KCTD12 PE=1 SV=1 - [KCD12_HUMAN]	3.69	1	1	1	1	2.00	3.69	1	1		0.00			325	35.7	5.64
Q6U841	Sodium-driven chloride bicarbonate exchanger OS=Homo sapiens OX=9606 GN=SLC4A10 PE=1 SV=1 - [S4A10_HUMAN]	3.67	1	1	1	1	2.58	3.67	1	1		0.00			1118	125.9	6.49
P05549	Transcription factor AP-2-alpha OS=Homo sapiens OX=9606 GN=TFAP2A PE=1 SV=1 - [AP2A_HUMAN]	3.66	1	1	1	1		0.00			3.33	3.66	1	1	437	48.0	8.02
P30613	Pyruvate kinase PKLR OS=Homo sapiens OX=9606 GN=PKLR PE=1 SV=2 - [KPYR_HUMAN]	3.66	1	1	1	1		0.00			3.21	3.66	1	1	574	61.8	7.74
Q9ULZ9	Matrix metalloproteinase-17 OS=Homo sapiens OX=9606 GN=MMP17 PE=1 SV=4 - [MMP17_HUMAN]	3.65	1	1	1	1		0.00			3.17	3.65	1	1	603	66.6	6.57
O94810	Regulator of G-protein signaling 11 OS=Homo sapiens OX=9606 GN=RGS11 PE=1 SV=2 - [RGS11_HUMAN]	3.64	1	1	1	1		0.00			0.00	3.64	1	1	467	52.9	8.78
P52630	Signal transducer and activator of transcription 2 OS=Homo sapiens OX=9606 GN=STAT2 PE=1 SV=1 - [STAT2_HUMAN]	3.64	1	1	1	1		0.00			3.65	3.64	1	1	851	97.9	5.49
P05160	Coagulation factor XIII B chain OS=Homo sapiens OX=9606 GN=F13B PE=1 SV=3 - [F13B_HUMAN]	3.63	1	1	1	1	3.33	3.63	1	1		0.00			661	75.5	6.39
Q96HE9	Proline-rich protein 11 OS=Homo sapiens OX=9606 GN=PRR11 PE=1 SV=1 - [PRR11_HUMAN]	3.61	1	1	1	1		0.00			2.70	3.61	1	1	360	40.1	10.11
Q9BYZ6	Rho-related BTB domain-containing protein 2 OS=Homo sapiens OX=9606 GN=RHOBTB2 PE=1 SV=2 - [RHBT2_HUMAN]	3.58	1	1	1	1		0.00			2.80	3.58	1	1	727	82.6	6.74
Q9NR21	Protein mono-ADP-ribosyltransferase PARP11 OS=Homo sapiens OX=9606 GN=PARP11 PE=1 SV=2 - [PAR11_HUMAN]	3.55	1	1	1	1	1.90	3.55	1	1		0.00			338	39.6	7.30
P08311	Cathepsin G OS=Homo sapiens OX=9606 GN=CTSG PE=1 SV=2 - [CATG_HUMAN]	3.53	1	1	1	1		0.00			0.00	3.53	1	1	255	28.8	11.19
Q9P0L2	Serine/threonine-protein kinase MARK1 OS=Homo sapiens OX=9606 GN=MARK1 PE=1 SV=2 - [MARK1_HUMAN]	3.52	1	1	1	1		0.00			3.41	3.52	1	1	795	88.9	9.36
Q9UHQ1	Nuclear prelamin A recognition factor OS=Homo sapiens OX=9606 GN=NARF PE=1 SV=1 - [NARF_HUMAN]	3.51	1	1	1	1		0.00			2.11	3.51	1	1	456	51.1	7.08
Q08828	Adenylate cyclase type 1 OS=Homo sapiens OX=9606 GN=ADCY1 PE=1 SV=2 - [ADCY1_HUMAN]	3.49	1	2	2	2	2.05	1.52	1	1	2.70	1.97	1	1	1119	123.4	8.43
Q9NYV8	Taste receptor type 2 member 14 OS=Homo sapiens OX=9606 GN=TAS2R14 PE=1 SV=1 - [T2R14_HUMAN]	3.47	1	1	1	1	0.00	3.47	1	1		0.00			317	36.1	10.10
Q92574	Hamartin OS=Homo sapiens OX=9606 GN=TSC1 PE=1 SV=2 - [TSC1_HUMAN]	3.44	1	1	1	1	3.13	3.44	1	1		0.00			1164	129.7	6.47
A2IDD5	Coiled-coil domain-containing protein 78 OS=Homo sapiens OX=9606 GN=CCDC78 PE=1 SV=1 - [CCD78_HUMAN]	3.42	1	1	1	2		0.00			2.31	3.42	1	2	438	48.5	8.16
P34913	Bifunctional epoxide hydrolase 2 OS=Homo sapiens OX=9606 GN=EPHX2 PE=1 SV=2 - [HYES_HUMAN]	3.42	1	1	1	1		0.00			2.97	3.42	1	1	555	62.6	6.28
O95125	Zinc finger protein 202 OS=Homo sapiens OX=9606 GN=ZNF202 PE=1 SV=4 - [ZN202_HUMAN]	3.40	1	1	1	1		0.00			3.09	3.40	1	1	648	74.7	5.97
Q8TBP0	TBC1 domain family member 16 OS=Homo sapiens OX=9606 GN=TBC1D16 PE=2 SV=1 - [TBC16_HUMAN]	3.39	1	1	1	1		0.00			2.69	3.39	1	1	767	86.3	5.82
Q9Y345	Sodium- and chloride-dependent glycine transporter 2 OS=Homo sapiens OX=9606 GN=SLC6A5 PE=1 SV=3 - [SC6A5_HUM	3.39	1	1	1	2	2.85	3.39	1	1	2.82	3.39	1	1	797	87.4	7.52
P50395	Rab GDP dissociation inhibitor beta OS=Homo sapiens OX=9606 GN=GDI2 PE=1 SV=2 - [GDIB_HUMAN]	3.37	1	1	1	1		0.00			2.31	3.37	1	1	445	50.6	6.47
Q9H257	Caspase recruitment domain-containing protein 9 OS=Homo sapiens OX=9606 GN=CARD9 PE=1 SV=2 - [CARD9_HUMAN]	3.36	1	1	1	1		0.00			2.64	3.36	1	1	536	62.2	6.07
Q9BZB8	Cytoplasmic polyadenylation element-binding protein 1 OS=Homo sapiens OX=9606 GN=CPEB1 PE=1 SV=1 - [CPEB1_HUM	3.36	1	1	1	1		0.00			2.58	3.36	1	1	566	62.6	7.61
Q8TE57	A disintegrin and metalloproteinase with thrombospondin motifs 16 OS=Homo sapiens OX=9606 GN=ADAMTS16 PE=2 SV=	3.35	1	1	1	1	4.26	3.35	1	1		0.00			1224	136.1	8.63
P12259	Coagulation factor V OS=Homo sapiens OX=9606 GN=F5 PE=1 SV=4 - [FA5_HUMAN]	3.33	1	4	4	5	11.96	2.65	3	4	2.60	0.67	1	1	2224	251.5	6.05
Q12849	G-rich sequence factor 1 OS=Homo sapiens OX=9606 GN=GRSF1 PE=1 SV=3 - [GRSF1_HUMAN]	3.33	1	1	1	3	8.35	3.33	1	3		0.00			480	53.1	6.19
O95831	Apoptosis-inducing factor 1, mitochondrial OS=Homo sapiens OX=9606 GN=AIFM1 PE=1 SV=1 - [AIFM1_HUMAN]	3.26	1	1	1	4	2.43	3.26	1	1	8.57	3.26	1	3	613	66.9	8.95
Q7Z3D6	D-glutamate cyclase, mitochondrial OS=Homo sapiens OX=9606 GN=DGLUCY PE=1 SV=2 - [GLUCM_HUMAN]	3.25	1	1	1	1		0.00			2.76	3.25	1	1	616	66.4	6.79
Q8NHS4	Clathrin heavy chain linker domain-containing protein 1 OS=Homo sapiens OX=9606 GN=CLHC1 PE=1 SV=3 - [CLHC1_HUN	3.24	1	1	1	1	2.25	3.24	1	1		0.00			586	67.2	6.49
Q9NZV7	Zinc finger imprinted 2 OS=Homo sapiens OX=9606 GN=ZIM2 PE=1 SV=1 - [ZIM2_HUMAN]	3.23	1	1	1	1		0.00			1.97	3.23	1	1	527	61.1	6.77
Q8WXF7	Atlastin-1 OS=Homo sapiens OX=9606 GN=ATL1 PE=1 SV=1 - [ATLA1_HUMAN]	3.23	1	1	1	1		0.00			2.29	3.23	1	1	558	63.5	6.18
Q5T1V6	Probable ATP-dependent RNA helicase DDX59 OS=Homo sapiens OX=9606 GN=DDX59 PE=1 SV=1 - [DDX59_HUMAN]	3.23	1	1	1	1		0.00			2.75	3.23	1	1	619	68.8	7.44
A8MVX0	Rho guanine nucleotide exchange factor 33 OS=Homo sapiens OX=9606 GN=ARHGEF33 PE=2 SV=2 - [ARG33_HUMAN]	3.20	1	1	1	1		0.00			3.76	3.20	1	1	844	94.6	6.81
Q15723	ETS-related transcription factor Elf-2 OS=Homo sapiens OX=9606 GN=ELF2 PE=1 SV=2 - [ELF2_HUMAN]	3.20	1	1	1	1		0.00			0.00	3.20	1	1	593	63.9	6.57
P42166	Lamina-associated polypeptide 2, isoform alpha OS=Homo sapiens OX=9606 GN=TMPO PE=1 SV=2 - [LAP2A_HUMAN]	3.17	1	1	1	1		0.00			2.41	3.17	1	1	694	75.4	7.66
O94760	N(G),N(G)-dimethylarginine dimethylaminohydrolase 1 OS=Homo sapiens OX=9606 GN=DDAH1 PE=1 SV=3 - [DDAH1_HUM	3.16	1	1	1	5	1.63	3.16	1	4	0.00	3.16	1	1	285	31.1	5.81
Q9HCM4	Band 4.1-like protein 5 OS=Homo sapiens OX=9606 GN=EPB41L5 PE=1 SV=3 - [E41L5_HUMAN]	3.14	1	1	1	1	2.71	3.14	1	1		0.00			733	81.8	6.58
P49746	Thrombospondin-3 OS=Homo sapiens OX=9606 GN=THBS3 PE=1 SV=1 - [TSP3_HUMAN]	3.14	1	1	1	1	3.03	3.14	1	1		0.00			956	104.1	4.65
Q96LT7	Guanine nucleotide exchange C9orf72 OS=Homo sapiens OX=9606 GN=C9orf72 PE=1 SV=2 - [CI072_HUMAN]	3.12	1	1	1	1	2.73	3.12	1	1		0.00			481	54.3	6.23
Q8TC26	Transmembrane protein 163 OS=Homo sapiens OX=9606 GN=TMEM163 PE=2 SV=1 - [TM163_HUMAN]	3.11	1	1	1	1		0.00			0.00	3.11	1	1	289	31.4	7.53
Q96EB6	NAD-dependent protein deacetylase sirtuin-1 OS=Homo sapiens OX=9606 GN=SIRT1 PE=1 SV=2 - [SIR1_HUMAN]	3.08	1	1	1	2	2.81	3.08	1	1	3.34	3.08	1	1	747	81.6	4.67
P29401	Transketolase OS=Homo sapiens OX=9606 GN=TKT PE=1 SV=3 - [TKT_HUMAN]	3.05	1	1	1	2		0.00			5.80	3.05	1	2	623	67.8	7.66
Q5H9T9	Fibrous sheath CABYR-binding protein OS=Homo sapiens OX=9606 GN=FSCB PE=2 SV=3 - [FSCB_HUMAN]	3.03	1	1	1	1	2.35	3.03	1	1		0.00			825	87.9	4.22
Q12879	Glutamate receptor ionotropic, NMDA 2A OS=Homo sapiens OX=9606 GN=GRIN2A PE=1 SV=1 - [NMDE1_HUMAN]	3.01	1	1	1	1	3.68	3.01	1	1		0.00			1464	165.2	7.11
Q9H094	Neuroblastoma breakpoint family member 3 OS=Homo sapiens OX=9606 GN=NBPF3 PE=2 SV=1 - [NBPF3_HUMAN]	3.00	1	1	1	3		0.00			0.00	3.00	1	3	633	72.9	4.50
Q9Y6F8	Testis-specific chromodomain protein Y 1 OS=Homo sapiens OX=9606 GN=CDY1 PE=1 SV=1 - [CDY1_HUMAN]	2.96	1	1	1	1	2.10	2.96	1	1		0.00			540	60.4	9.10
O00757	Fructose-1,6-bisphosphatase isozyme 2 OS=Homo sapiens OX=9606 GN=FBP2 PE=1 SV=2 - [F16P2_HUMAN]	2.95	1	1	1	2		0.00			4.08	2.95	1	2	339	36.7	7.23
Q96P44	Collagen alpha-1(XXI) chain OS=Homo sapiens OX=9606 GN=COL21A1 PE=2 SV=1 - [COLA1_HUMAN]	2.93	1	1	1	1		0.00			3.35	2.93	1	1	957	99.3	8.32
Q7Z7M1	Adhesion G-protein coupled receptor D2 OS=Homo sapiens OX=9606 GN=ADGRD2 PE=2 SV=1 - [AGRD2_HUMAN]	2.91	1	1	1	1		0.00			3.81	2.91	1	1	963	104.0	8.02
P35749	Myosin-11 OS=Homo sapiens OX=9606 GN=MYH11 PE=1 SV=3 - [MYH11_HUMAN]	2.89	2	1	4	8	3.88	0.81	1	1	19.73	2.89	4	7	1972	227.2	5.50
Q9UIL4	Kinesin-like protein KIF25 OS=Homo sapiens OX=9606 GN=KIF25 PE=2 SV=2 - [KIF25_HUMAN]	2.86	1	1	1	1	0.00	2.86	1	1		0.00			384	40.7	8.31
Q8NDA8	Maestro heat-like repeat-containing protein family member 1 OS=Homo sapiens OX=9606 GN=MROH1 PE=2 SV=3 - [MROH	2.86	1	1	1	1		0.00			3.95	2.86	1	1	1641	181.1	6.89
Q9HCN3	Post-GPI attachment to proteins factor 6 OS=Homo sapiens OX=9606 GN=PGAP6 PE=1 SV=3 - [PGAP6_HUMAN]	2.85	1	1	1	1		0.00			2.56	2.85	1	1	771	84.7	7.53
P36871	Phosphoglucomutase-1 OS=Homo sapiens OX=9606 GN=PGM1 PE=1 SV=3 - [PGM1_HUMAN]	2.85	1	1	1	3		0.00			6.82	2.85	1	3	562	61.4	6.76
Q9BVL2	Nucleoporin p58/p45 OS=Homo sapiens OX=9606 GN=NUP58 PE=1 SV=1 - [NUP58_HUMAN]	2.84	1	1	1	1		0.00			0.00	2.84	1	1	599	60.9	9.33
Q1ED39	Lysine-rich nucleolar protein 1 OS=Homo sapiens OX=9606 GN=KNOP1 PE=1 SV=1 - [KNOP1_HUMAN]	2.84	1	1	1	1	2.52	2.84	1	1		0.00			458	51.6	9.86
Q9HCJ0	Trinucleotide repeat-containing gene 6C protein OS=Homo sapiens OX=9606 GN=TNRC6C PE=1 SV=3 - [TNR6C_HUMAN]	2.84	1	1	1	1		0.00			4.29	2.84	1	1	1690	175.9	6.96
Q9UK80	Ubiquitin carboxyl-terminal hydrolase 21 OS=Homo sapiens OX=9606 GN=USP21 PE=1 SV=1 - [UBP21_HUMAN]	2.83	1	1	1	1		0.00			2.15	2.83	1	1	565	62.6	9.86
P36894	Bone morphogenetic protein receptor type-1A OS=Homo sapiens OX=9606 GN=BMPR1A PE=1 SV=2 - [BMR1A_HUMAN]	2.82	1	1	1	1		0.00			2.56	2.82	1	1	532	60.2	7.58
P54253	Ataxin-1 OS=Homo sapiens OX=9606 GN=ATXN1 PE=1 SV=2 - [ATX1_HUMAN]	2.82	1	1	1	1		0.00			3.07	2.82	1	1	815	86.9	8.35
C9JN71	Zinc finger protein 878 OS=Homo sapiens OX=9606 GN=ZNF878 PE=3 SV=2 - [ZN878_HUMAN]	2.82	1	1	1	1		0.00			1.75	2.82	1	1	531	61.5	9.45
Q9Y2G5	GDP-fucose protein O-fucosyltransferase 2 OS=Homo sapiens OX=9606 GN=POFUT2 PE=1 SV=3 - [OFUT2_HUMAN]	2.80	1	1	1	1		0.00			2.37	2.80	1	1	429	49.9	6.60
O15427	Monocarboxylate transporter 4 OS=Homo sapiens OX=9606 GN=SLC16A3 PE=1 SV=1 - [MOT4_HUMAN]	2.80	1	1	1	2		0.00			5.01	2.80	1	2	465	49.4	7.96
Q8NEZ2	Vacuolar protein sorting-associated protein 37A OS=Homo sapiens OX=9606 GN=VPS37A PE=1 SV=1 - [VP37A_HUMAN]	2.77	1	1	1	1	1.37	2.77	1	1		0.00			397	44.3	5.57
Q8IWU6	Extracellular sulfatase Sulf-1 OS=Homo sapiens OX=9606 GN=SULF1 PE=1 SV=1 - [SULF1_HUMAN]	2.76	1	1	1	1		0.00			2.50	2.76	1	1	871	101.0	9.09
P52333	Tyrosine-protein kinase JAK3 OS=Homo sapiens OX=9606 GN=JAK3 PE=1 SV=2 - [JAK3_HUMAN]	2.76	1	1	1	1		0.00			2.99	2.76	1	1	1124	125.0	7.18
Q9NXH9	tRNA (guanine(26)-N(2))-dimethyltransferase OS=Homo sapiens OX=9606 GN=TRMT1 PE=1 SV=1 - [TRM1_HUMAN]	2.73	1	1	1	1		0.00			4.19	2.73	1	1	659	72.2	7.64
Q8IX18	Probable ATP-dependent RNA helicase DHX40 OS=Homo sapiens OX=9606 GN=DHX40 PE=1 SV=2 - [DHX40_HUMAN]	2.70	1	1	1	1		0.00			2.99	2.70	1	1	779	88.5	8.65
Q8TDC3	Serine/threonine-protein kinase BRSK1 OS=Homo sapiens OX=9606 GN=BRSK1 PE=1 SV=2 - [BRSK1_HUMAN]	2.70	1	1	1	1	4.20	2.70	1	1		0.00			778	85.0	9.32
Q08AM6	Protein VAC14 homolog OS=Homo sapiens OX=9606 GN=VAC14 PE=1 SV=1 - [VAC14_HUMAN]	2.69	1	1	1	1	2.60	2.69	1	1		0.00			782	87.9	6.13
O75643	U5 small nuclear ribonucleoprotein 200 kDa helicase OS=Homo sapiens OX=9606 GN=SNRNP200 PE=1 SV=2 - [U520_HUM/	2.67	1	2	2	2	5.52	2.67	2	2		0.00			2136	244.4	6.06
Q969T7	7-methylguanosine phosphate-specific 5'-nucleotidase OS=Homo sapiens OX=9606 GN=NT5C3B PE=1 SV=4 - [5NT3B_HUM	2.67	1	1	1	3	3.94	2.67	1	2	1.64	2.67	1	1	300	34.4	6.38
O95905	Protein ecdysoneless homolog OS=Homo sapiens OX=9606 GN=ECD PE=1 SV=1 - [ECD_HUMAN]	2.64	1	1	1	1	1.68	2.64	1	1		0.00			644	72.7	4.87
Q7Z3F1	Integral membrane protein GPR155 OS=Homo sapiens OX=9606 GN=GPR155 PE=2 SV=2 - [GP155_HUMAN]	2.64	1	1	1	1	2.78	2.64	1	1		0.00			870	96.9	6.84
Q9UPQ4	E3 ubiquitin-protein ligase TRIM35 OS=Homo sapiens OX=9606 GN=TRIM35 PE=1 SV=2 - [TRI35_HUMAN]	2.64	1	1	1	1		0.00			2.22	2.64	1	1	493	56.5	7.11
Q9NTN9	Semaphorin-4G OS=Homo sapiens OX=9606 GN=SEMA4G PE=1 SV=1 - [SEM4G_HUMAN]	2.63	1	1	1	1		0.00			2.45	2.63	1	1	838	91.4	7.84
Q86WZ0	HEAT repeat-containing protein 4 OS=Homo sapiens OX=9606 GN=HEATR4 PE=2 SV=2 - [HEAT4_HUMAN]	2.63	1	1	1	1	2.29	2.63	1	1		0.00			1026	117.1	9.11
Q86X52	Chondroitin sulfate synthase 1 OS=Homo sapiens OX=9606 GN=CHSY1 PE=1 SV=3 - [CHSS1_HUMAN]	2.62	1	1	1	1	2.72	2.62	1	1		0.00			802	91.7	9.23
Q3KQU3	MAP7 domain-containing protein 1 OS=Homo sapiens OX=9606 GN=MAP7D1 PE=1 SV=1 - [MA7D1_HUMAN]	2.62	5	2	2	2		0.00			4.58	2.62	2	2	841	92.8	10.11
Q8N3U4	Cohesin subunit SA-2 OS=Homo sapiens OX=9606 GN=STAG2 PE=1 SV=3 - [STAG2_HUMAN]	2.60	3	2	2	2	0.00	1.87	1	1	0.00	0.73	1	1	1231	141.2	5.43
Q6PJT7	Zinc finger CCCH domain-containing protein 14 OS=Homo sapiens OX=9606 GN=ZC3H14 PE=1 SV=1 - [ZC3HE_HUMAN]	2.58	1	1	1	1	2.48	2.58	1	1		0.00			736	82.8	7.31
Q6P5Q4	Leiomodin-2 OS=Homo sapiens OX=9606 GN=LMOD2 PE=1 SV=2 - [LMOD2_HUMAN]	2.56	1	1	1	1		0.00			3.11	2.56	1	1	547	61.6	5.78
P15529	Membrane cofactor protein OS=Homo sapiens OX=9606 GN=CD46 PE=1 SV=3 - [MCP_HUMAN]	2.55	1	1	1	1	0.00	2.55	1	1		0.00			392	43.7	6.74
Q7Z2K6	Endoplasmic reticulum metallopeptidase 1 OS=Homo sapiens OX=9606 GN=ERMP1 PE=1 SV=2 - [ERMP1_HUMAN]	2.54	1	1	1	1		0.00			3.11	2.54	1	1	904	100.2	7.52
P09913	Interferon-induced protein with tetratricopeptide repeats 2 OS=Homo sapiens OX=9606 GN=IFIT2 PE=1 SV=1 - [IFIT2_HU	2.54	1	1	1	1		0.00			2.78	2.54	1	1	472	54.6	6.76
Q68BL8	Olfactomedin-like protein 2B OS=Homo sapiens OX=9606 GN=OLFML2B PE=2 SV=2 - [OLM2B_HUMAN]	2.53	1	1	1	1		0.00			2.81	2.53	1	1	750	83.9	5.20
Q96Q89	Kinesin-like protein KIF20B OS=Homo sapiens OX=9606 GN=KIF20B PE=1 SV=3 - [KI20B_HUMAN]	2.53	1	2	2	3	6.86	2.53	2	3		0.00			1820	210.5	5.67
Q96PQ6	Zinc finger protein 317 OS=Homo sapiens OX=9606 GN=ZNF317 PE=1 SV=2 - [ZN317_HUMAN]	2.52	1	1	1	1	0.00	2.52	1	1		0.00			595	67.9	8.98
Q9Y5E4	Protocadherin beta-5 OS=Homo sapiens OX=9606 GN=PCDHB5 PE=2 SV=1 - [PCDB5_HUMAN]	2.52	1	1	1	1		0.00			2.13	2.52	1	1	795	86.4	5.00
Q9BYC2	Succinyl-CoA:3-ketoacid coenzyme A transferase 2, mitochondrial OS=Homo sapiens OX=9606 GN=OXCT2 PE=2 SV=2 - [S(	2.51	1	1	1	1		0.00			0.00	2.51	1	1	517	56.1	7.14
A6NES4	Maestro heat-like repeat-containing protein family member 2A OS=Homo sapiens OX=9606 GN=MROH2A PE=4 SV=4 - [MF	2.51	1	2	2	3	3.62	1.49	1	2	2.91	1.02	1	1	1674	189.4	6.68
Q9HDB5	Neurexin-3-beta OS=Homo sapiens OX=9606 GN=NRXN3 PE=1 SV=4 - [NRX3B_HUMAN]	2.51	2	1	1	1		0.00			1.70	2.51	1	1	637	69.3	7.21
Q14161	ARF GTPase-activating protein GIT2 OS=Homo sapiens OX=9606 GN=GIT2 PE=1 SV=2 - [GIT2_HUMAN]	2.50	1	1	1	1		0.00			3.50	2.50	1	1	759	84.5	7.23

P04180	Phosphatidylcholine-sterol acyltransferase OS=Homo sapiens OX=9606 GN=LCAT PE=1 SV=1 - [LCAT_HUMAN]	2.50	1	1	1	1	0.00	2.50	1	1		0.00			440	49.5	6.11
Q96QU8	Exportin-6 OS=Homo sapiens OX=9606 GN=XPO6 PE=1 SV=1 - [XPO6_HUMAN]	2.49	1	1	1	1	0.00	2.49	1	1		0.00			1125	128.8	6.35
P49916	DNA ligase 3 OS=Homo sapiens OX=9606 GN=LIG3 PE=1 SV=2 - [DNLI3_HUMAN]	2.48	1	1	1	1	3.46	2.48	1	1		0.00			1009	112.8	9.01
Q96JS3	PiggyBac transposable element-derived protein 1 OS=Homo sapiens OX=9606 GN=PGBD1 PE=1 SV=1 - [PGBD1_HUMAN]	2.47	1	1	1	1	3.36	2.47	1	1		0.00			809	92.5	5.63
P04040	Catalase OS=Homo sapiens OX=9606 GN=CAT PE=1 SV=3 - [CATA_HUMAN]	2.47	1	1	1	4		0.00			10.46	2.47	1	4	527	59.7	7.39
Q9NV70	Exocyst complex component 1 OS=Homo sapiens OX=9606 GN=EXOC1 PE=1 SV=4 - [EXOC1_HUMAN]	2.46	1	1	1	1	2.75	2.46	1	1		0.00			894	101.9	6.61
Q96S53	Dual specificity testis-specific protein kinase 2 OS=Homo sapiens OX=9606 GN=TESK2 PE=1 SV=1 - [TESK2_HUMAN]	2.45	1	1	1	1		0.00			2.81	2.45	1	1	571	63.6	7.06
P17655	Calpain-2 catalytic subunit OS=Homo sapiens OX=9606 GN=CAPN2 PE=1 SV=6 - [CAN2_HUMAN]	2.43	1	1	1	1	2.28	2.43	1	1		0.00			700	79.9	4.98
P16383	Intron Large complex component GCFC2 OS=Homo sapiens OX=9606 GN=GCFC2 PE=1 SV=2 - [GCFC2_HUMAN]	2.43	1	1	1	1		0.00			2.60	2.43	1	1	781	89.3	5.99
P14923	Junction plakoglobin OS=Homo sapiens OX=9606 GN=JUP PE=1 SV=3 - [PLAK_HUMAN]	2.42	1	1	1	1	2.74	2.42	1	1		0.00			745	81.7	6.14
Q6IA86	Elongator complex protein 2 OS=Homo sapiens OX=9606 GN=ELP2 PE=1 SV=2 - [ELP2_HUMAN]	2.42	1	1	1	1		0.00			2.62	2.42	1	1	826	92.4	5.96
Q6EMB2	Tubulin polyglutamylase TTLL5 OS=Homo sapiens OX=9606 GN=TTLL5 PE=1 SV=3 - [TTLL5_HUMAN]	2.42	1	2	2	2		0.00			4.24	2.42	2	2	1281	143.5	8.63
D6REC4	Cilia- and flagella-associated protein 99 OS=Homo sapiens OX=9606 GN=CFAP99 PE=3 SV=1 - [CFA99_HUMAN]	2.40	1	1	1	1	0.00	2.40	1	1		0.00			459	52.3	10.55
Q70EK9	Ubiquitin carboxyl-terminal hydrolase 51 OS=Homo sapiens OX=9606 GN=USP51 PE=1 SV=1 - [UBP51_HUMAN]	2.39	1	1	1	2		0.00			2.65	2.39	1	2	711	79.7	8.37
Q8TEL6	Short transient receptor potential channel 4-associated protein OS=Homo sapiens OX=9606 GN=TRPC4AP PE=1 SV=2 - [TR	2.38	1	1	1	1		0.00			2.93	2.38	1	1	797	90.8	7.61
Q8N323	NXPE family member 1 OS=Homo sapiens OX=9606 GN=NXPE1 PE=1 SV=2 - [NXPE1_HUMAN]	2.38	1	1	1	1		0.00			1.74	2.38	1	1	547	63.1	8.56
A0MZ66	Shootin-1 OS=Homo sapiens OX=9606 GN=SHTN1 PE=1 SV=4 - [SHOT1_HUMAN]	2.38	1	1	1	1	2.73	2.38	1	1		0.00			631	71.6	5.33
Q96J84	Kin of IRRE-like protein 1 OS=Homo sapiens OX=9606 GN=KIRREL1 PE=1 SV=2 - [KIRR1_HUMAN]	2.38	1	1	1	1		0.00			2.63	2.38	1	1	757	83.5	5.73
Q92954	Proteoglycan 4 OS=Homo sapiens OX=9606 GN=PRG4 PE=1 SV=3 - [PRG4_HUMAN]	2.35	1	2	2	6	5.94	2.35	2	3	8.97	2.35	2	3	1404	151.0	9.51
Q9P1W8	Signal-regulatory protein gamma OS=Homo sapiens OX=9606 GN=SIRPG PE=1 SV=3 - [SIRPG_HUMAN]	2.33	3	1	1	1		0.00			0.00	2.33	1	1	387	42.5	7.17
Q5HY64	Putative protein FAM47C OS=Homo sapiens OX=9606 GN=FAM47C PE=2 SV=1 - [FA47C_HUMAN]	2.32	1	1	1	1	0.00	2.32	1	1		0.00			1035	115.3	7.18
Q6PJP8	DNA cross-link repair 1A protein OS=Homo sapiens OX=9606 GN=DCLRE1A PE=1 SV=3 - [DCR1A_HUMAN]	2.31	1	1	1	1		0.00			2.98	2.31	1	1	1040	116.3	7.97
Q9NS93	Transmembrane 7 superfamily member 3 OS=Homo sapiens OX=9606 GN=TM7SF3 PE=2 SV=1 - [TM7S3_HUMAN]	2.28	1	1	1	1		0.00			0.00	2.28	1	1	570	64.1	7.01
Q99490	Arf-GAP with GTPase, ANK repeat and PH domain-containing protein 2 OS=Homo sapiens OX=9606 GN=AGAP2 PE=1 SV=2	2.27	1	1	1	1		0.00			3.25	2.27	1	1	1192	124.6	9.89
P0CK97	Solute carrier family 35 member E2A OS=Homo sapiens OX=9606 GN=SLC35E2A PE=1 SV=1 - [S35E2_HUMAN]	2.26	2	1	1	1		0.00			0.00	2.26	1	1	266	29.1	8.09
Q9BYX2	TBC1 domain family member 2A OS=Homo sapiens OX=9606 GN=TBC1D2 PE=1 SV=3 - [TBD2A_HUMAN]	2.26	1	1	1	1	2.54	2.26	1	1		0.00			928	105.3	6.58
O60331	Phosphatidylinositol 4-phosphate 5-kinase type-1 gamma OS=Homo sapiens OX=9606 GN=PIP5K1C PE=1 SV=2 - [PI51C_H	2.25	1	1	1	1		0.00			0.00	2.25	1	1	668	73.2	5.29
Q9UJ41	Rab5 GDP/GTP exchange factor OS=Homo sapiens OX=9606 GN=RABGEF1 PE=1 SV=3 - [RABX5_HUMAN]	2.24	1	1	1	1		0.00			2.11	2.24	1	1	491	56.9	7.02
Q9NPI6	mRNA-decapping enzyme 1A OS=Homo sapiens OX=9606 GN=DCP1A PE=1 SV=3 - [DCP1A_HUMAN]	2.23	1	1	1	1		0.00			2.49	2.23	1	1	582	63.2	6.25
Q92805	Golgin subfamily A member 1 OS=Homo sapiens OX=9606 GN=GOLGA1 PE=1 SV=3 - [GOGA1_HUMAN]	2.22	1	1	1	1		0.00			2.31	2.22	1	1	767	88.1	5.27
O95954	Formimidoyltransferase-cyclodeaminase OS=Homo sapiens OX=9606 GN=FTCD PE=1 SV=2 - [FTCD_HUMAN]	2.22	1	1	1	1		0.00			2.46	2.22	1	1	541	58.9	5.74
P24298	Alanine aminotransferase 1 OS=Homo sapiens OX=9606 GN=GPT PE=1 SV=3 - [ALAT1_HUMAN]	2.22	1	1	1	1		0.00			0.00	2.22	1	1	496	54.6	7.18
Q9UHL9	General transcription factor II-I repeat domain-containing protein 1 OS=Homo sapiens OX=9606 GN=GTF2IRD1 PE=1 SV=	2.19	1	1	1	1		0.00			2.35	2.19	1	1	959	106.0	6.87
Q6ZUK4	Transmembrane protein 26 OS=Homo sapiens OX=9606 GN=TMEM26 PE=1 SV=1 - [TMM26_HUMAN]	2.17	1	1	1	1	0.00	2.17	1	1		0.00			368	41.6	6.87
Q86X29	Lipolysis-stimulated lipoprotein receptor OS=Homo sapiens OX=9606 GN=LSR PE=1 SV=4 - [LSR_HUMAN]	2.16	1	1	1	1		0.00			2.71	2.16	1	1	649	71.4	7.97
P54198	Protein HIRA OS=Homo sapiens OX=9606 GN=HIRA PE=1 SV=2 - [HIRA_HUMAN]	2.16	1	1	1	1		0.00			2.73	2.16	1	1	1017	111.8	8.07
Q6ZVL6	UPF0606 protein KIAA1549L OS=Homo sapiens OX=9606 GN=KIAA1549L PE=2 SV=2 - [K154L_HUMAN]	2.16	1	2	2	2	2.77	1.08	1	1	2.78	1.08	1	1	1849	198.9	8.31
O14709	Zinc finger protein 197 OS=Homo sapiens OX=9606 GN=ZNF197 PE=2 SV=1 - [ZN197_HUMAN]	2.14	1	1	1	1		0.00			2.32	2.14	1	1	1029	118.8	8.59
Q6ZMR5	Transmembrane protease serine 11A OS=Homo sapiens OX=9606 GN=TMPRSS11A PE=1 SV=1 - [TM11A_HUMAN]	2.14	1	1	1	1		0.00			1.61	2.14	1	1	421	47.5	9.22
P08575	Receptor-type tyrosine-protein phosphatase C OS=Homo sapiens OX=9606 GN=PTPRC PE=1 SV=3 - [PTPRC_HUMAN]	2.14	1	2	2	2		0.00			5.93	2.14	2	2	1306	147.4	6.15
Q9H0I3	Coiled-coil domain-containing protein 113 OS=Homo sapiens OX=9606 GN=CCDC113 PE=1 SV=1 - [CC113_HUMAN]	2.12	1	1	1	1		0.00			1.76	2.12	1	1	377	44.2	8.73
P35711	Transcription factor SOX-5 OS=Homo sapiens OX=9606 GN=SOX5 PE=1 SV=3 - [SOX5_HUMAN]	2.10	1	1	1	1	0.00	2.10	1	1		0.00			763	84.0	6.60
Q9UHN6	Cell surface hyaluronidase OS=Homo sapiens OX=9606 GN=CEMIP2 PE=1 SV=1 - [CEIP2_HUMAN]	2.10	1	1	1	3		0.00			2.91	2.10	1	3	1383	154.3	8.15
Q08050	Forkhead box protein M1 OS=Homo sapiens OX=9606 GN=FOXM1 PE=1 SV=3 - [FOXM1_HUMAN]	2.10	1	1	1	1	2.40	2.10	1	1		0.00			763	84.2	7.91
Q8NHU2	Cilia- and flagella-associated protein 61 OS=Homo sapiens OX=9606 GN=CFAP61 PE=2 SV=3 - [CFA61_HUMAN]	2.10	1	1	1	1	3.51	2.10	1	1		0.00			1237	141.3	6.16
Q8NAP3	Zinc finger and BTB domain-containing protein 38 OS=Homo sapiens OX=9606 GN=ZBTB38 PE=1 SV=2 - [ZBT38_HUMAN]	2.09	1	1	1	1	3.12	2.09	1	1		0.00			1195	134.2	8.03
O60763	General vesicular transport factor p115 OS=Homo sapiens OX=9606 GN=USO1 PE=1 SV=2 - [USO1_HUMAN]	2.08	1	1	1	1		0.00			3.33	2.08	1	1	962	107.8	4.91
Q8N5A5	Zinc finger CCCH-type with G patch domain-containing protein OS=Homo sapiens OX=9606 GN=ZGPAT PE=1 SV=3 - [ZGP	2.07	1	1	1	1	0.00	2.07	1	1		0.00			531	57.3	5.43
Q8TC59	Piwi-like protein 2 OS=Homo sapiens OX=9606 GN=PIWIL2 PE=1 SV=1 - [PIWL2_HUMAN]	2.06	1	1	1	1		0.00			2.67	2.06	1	1	973	109.8	8.98
Q9P2E9	Ribosome-binding protein 1 OS=Homo sapiens OX=9606 GN=RRBP1 PE=1 SV=5 - [RRBP1_HUMAN]	2.06	1	1	1	1		0.00			2.53	2.06	1	1	1410	152.4	8.60
Q5W041	Armadillo repeat-containing protein 3 OS=Homo sapiens OX=9606 GN=ARMC3 PE=2 SV=2 - [ARMC3_HUMAN]	2.06	1	1	1	1		0.00			2.88	2.06	1	1	872	96.3	6.21
O43286	Beta-1,4-galactosyltransferase 5 OS=Homo sapiens OX=9606 GN=B4GALT5 PE=1 SV=1 - [B4GT5_HUMAN]	2.06	2	1	1	1		0.00			0.00	2.06	1	1	388	45.1	8.15
Q6P9F0	Coiled-coil domain-containing protein 62 OS=Homo sapiens OX=9606 GN=CCDC62 PE=1 SV=2 - [CCD62_HUMAN]	2.05	1	1	1	3	4.52	2.05	1	3		0.00			684	77.7	6.02
Q6IQ23	Pleckstrin homology domain-containing family A member 7 OS=Homo sapiens OX=9606 GN=PLEKHA7 PE=1 SV=2 - [PKHA7	2.05	1	1	2	2		0.00			3.66	2.05	2	2	1121	127.1	9.35
Q16666	Gamma-interferon-inducible protein 16 OS=Homo sapiens OX=9606 GN=IFI16 PE=1 SV=3 - [IF16_HUMAN]	2.04	1	1	1	1	2.57	2.04	1	1		0.00			785	88.2	9.28
Q5TIA1	Meiosis inhibitor protein 1 OS=Homo sapiens OX=9606 GN=MEI1 PE=2 SV=2 - [MEI1_HUMAN]	2.04	1	1	1	1		0.00			2.88	2.04	1	1	1274	141.1	6.70
Q9NR80	Rho guanine nucleotide exchange factor 4 OS=Homo sapiens OX=9606 GN=ARHGEF4 PE=1 SV=3 - [ARHG4_HUMAN]	2.03	1	1	1	2		0.00			2.02	2.03	1	2	690	79.0	7.01
Q06124	Tyrosine-protein phosphatase non-receptor type 11 OS=Homo sapiens OX=9606 GN=PTPN11 PE=1 SV=3 - [PTN11_HUMAN	2.02	1	1	1	1		0.00			2.30	2.02	1	1	593	68.0	7.30
O94973	AP-2 complex subunit alpha-2 OS=Homo sapiens OX=9606 GN=AP2A2 PE=1 SV=2 - [AP2A2_HUMAN]	2.02	2	1	1	2		0.00			2.64	2.02	1	2	939	103.9	6.96
Q9Y446	Plakophilin-3 OS=Homo sapiens OX=9606 GN=PKP3 PE=1 SV=1 - [PKP3_HUMAN]	2.01	1	1	1	1	2.43	2.01	1	1		0.00			797	87.0	9.32
Q53R41	FAST kinase domain-containing protein 1, mitochondrial OS=Homo sapiens OX=9606 GN=FASTKD1 PE=1 SV=1 - [FAKD1_H	2.01	1	1	1	1		0.00			2.71	2.01	1	1	847	97.3	7.74
Q9BQN1	Protein FAM83C OS=Homo sapiens OX=9606 GN=FAM83C PE=1 SV=3 - [FA83C_HUMAN]	2.01	1	1	1	1		0.00			0.00	2.01	1	1	747	81.0	8.07
Q6PJF5	Inactive rhomboid protein 2 OS=Homo sapiens OX=9606 GN=RHBDF2 PE=1 SV=2 - [RHDF2_HUMAN]	1.99	1	1	1	1		0.00			1.95	1.99	1	1	856	96.6	8.82
Q7RTW8	Otoancorin OS=Homo sapiens OX=9606 GN=OTOA PE=1 SV=1 - [OTOAN_HUMAN]	1.99	1	1	1	1		0.00			3.15	1.99	1	1	1153	128.5	5.82
Q01844	RNA-binding protein EWS OS=Homo sapiens OX=9606 GN=EWSR1 PE=1 SV=1 - [EWS_HUMAN]	1.98	1	1	1	1		0.00			0.00	1.98	1	1	656	68.4	9.33
Q9UBF2	Coatomer subunit gamma-2 OS=Homo sapiens OX=9606 GN=COPG2 PE=1 SV=1 - [COPG2_HUMAN]	1.95	1	1	1	1	2.44	1.95	1	1		0.00			871	97.6	5.81
Q8IYX0	Zinc finger protein 679 OS=Homo sapiens OX=9606 GN=ZNF679 PE=1 SV=2 - [ZN679_HUMAN]	1.95	1	1	1	1	1.48	1.95	1	1		0.00			411	47.1	8.91
Q9H6U6	BCAS3 microtubule associated cell migration factor OS=Homo sapiens OX=9606 GN=BCAS3 PE=1 SV=3 - [BCAS3_HUMAN]	1.94	1	1	1	1		0.00			2.90	1.94	1	1	928	101.2	6.70
Q9NPJ1	McKusick-Kaufman/Bardet-Biedl syndromes putative chaperonin OS=Homo sapiens OX=9606 GN=MKKS PE=1 SV=1 - [MKK	1.93	1	1	1	1	0.00	1.93	1	1		0.00			570	62.3	7.08
Q9Y5Q5	Atrial natriuretic peptide-converting enzyme OS=Homo sapiens OX=9606 GN=CORIN PE=1 SV=2 - [CORIN_HUMAN]	1.92	1	1	1	1	2.55	1.92	1	1		0.00			1042	116.4	5.02
Q8IZX4	Transcription initiation factor TFIID subunit 1-like OS=Homo sapiens OX=9606 GN=TAF1L PE=1 SV=1 - [TAF1L_HUMAN]	1.92	1	1	1	2	2.78	1.92	1	2		0.00			1826	207.2	5.40
A6NHR9	Structural maintenance of chromosomes flexible hinge domain-containing protein 1 OS=Homo sapiens OX=9606 GN=SMCH	1.90	1	1	1	1	0.00	1.90	1	1		0.00			2005	226.2	7.30
Q9Y2G9	Protein strawberry notch homolog 2 OS=Homo sapiens OX=9606 GN=SBNO2 PE=2 SV=3 - [SBNO2_HUMAN]	1.90	1	1	1	1	2.99	1.90	1	1		0.00			1366	150.2	6.52
Q8IX07	Zinc finger protein ZFPM1 OS=Homo sapiens OX=9606 GN=ZFPM1 PE=1 SV=2 - [FOG1_HUMAN]	1.89	1	1	1	1		0.00			2.19	1.89	1	1	1006	104.8	7.87
Q96JG9	Zinc finger protein 469 OS=Homo sapiens OX=9606 GN=ZNF469 PE=2 SV=3 - [ZN469_HUMAN]	1.89	1	2	2	2	3.97	0.82	1	1	3.33	1.07	1	1	3925	409.9	7.72
Q4KWH8	1-phosphatidylinositol 4,5-bisphosphate phosphodiesterase eta-1 OS=Homo sapiens OX=9606 GN=PLCH1 PE=1 SV=1 - [PL(	1.89	1	2	2	2	0.00	1.42	1	1	1.70	0.47	1	1	1693	189.1	7.74
Q9NZI6	Transcription factor CP2-like protein 1 OS=Homo sapiens OX=9606 GN=TFCP2L1 PE=1 SV=1 - [TF2L1_HUMAN]	1.88	1	1	1	2		0.00			0.00	1.88	1	2	479	54.6	7.01
Q9UL18	Protein argonaute-1 OS=Homo sapiens OX=9606 GN=AGO1 PE=1 SV=3 - [AGO1_HUMAN]	1.87	2	1	1	1		0.00			2.14	1.87	1	1	857	97.2	9.16
Q13308	Inactive tyrosine-protein kinase 7 OS=Homo sapiens OX=9606 GN=PTK7 PE=1 SV=2 - [PTK7_HUMAN]	1.87	1	1	1	1	2.92	1.87	1	1		0.00			1070	118.3	7.09
Q68DU8	BTB/POZ domain-containing protein KCTD16 OS=Homo sapiens OX=9606 GN=KCTD16 PE=1 SV=1 - [KCD16_HUMAN]	1.87	1	1	1	1		0.00			1.73	1.87	1	1	428	49.1	8.09
Q8N159	N-acetylglutamate synthase, mitochondrial OS=Homo sapiens OX=9606 GN=NAGS PE=1 SV=1 - [NAGS_HUMAN]	1.87	1	1	1	1		0.00			2.03	1.87	1	1	534	58.1	8.90
O75027	Iron-sulfur clusters transporter ABCB7, mitochondrial OS=Homo sapiens OX=9606 GN=ABCB7 PE=1 SV=2 - [ABCB7_HUMA	1.86	1	1	1	1		0.00			2.36	1.86	1	1	752	82.6	9.33
Q96JP9	Cadherin-related family member 1 OS=Homo sapiens OX=9606 GN=CDHR1 PE=1 SV=2 - [CDHR1_HUMAN]	1.86	1	1	1	1		0.00			2.09	1.86	1	1	859	93.5	5.54
Q8WY54	Protein phosphatase 1E OS=Homo sapiens OX=9606 GN=PPM1E PE=1 SV=3 - [PPM1E_HUMAN]	1.85	1	1	1	1		0.00			3.18	1.85	1	1	755	83.9	5.01
Q6AWC2	Protein WWC2 OS=Homo sapiens OX=9606 GN=WWC2 PE=1 SV=2 - [WWC2_HUMAN]	1.85	1	1	1	1	2.08	1.85	1	1		0.00			1192	133.8	5.53
Q8N2N9	Ankyrin repeat domain-containing protein 36B OS=Homo sapiens OX=9606 GN=ANKRD36B PE=1 SV=4 - [AN36B_HUMAN]	1.85	2	1	1	1		0.00			0.00	1.85	1	1	1353	153.5	8.85
P11171	Protein 4.1 OS=Homo sapiens OX=9606 GN=EPB41 PE=1 SV=4 - [EPB41_HUMAN]	1.85	1	1	1	2		0.00			5.74	1.85	1	2	864	97.0	5.58
P39086	Glutamate receptor ionotropic, kainate 1 OS=Homo sapiens OX=9606 GN=GRIK1 PE=1 SV=1 - [GRIK1_HUMAN]	1.85	1	1	1	1		0.00			2.16	1.85	1	1	918	103.9	7.08
O75533	Splicing factor 3B subunit 1 OS=Homo sapiens OX=9606 GN=SF3B1 PE=1 SV=3 - [SF3B1_HUMAN]	1.84	1	1	1	2	2.47	1.84	1	2		0.00			1304	145.7	7.09
Q96KG7	Multiple epidermal growth factor-like domains protein 10 OS=Homo sapiens OX=9606 GN=MEGF10 PE=1 SV=1 - [MEG10_H	1.84	1	1	1	1	0.00	1.84	1	1		0.00			1140	122.1	6.87
P11597	Cholesteryl ester transfer protein OS=Homo sapiens OX=9606 GN=CETP PE=1 SV=2 - [CETP_HUMAN]	1.83	1	1	1	1	1.96	1.83	1	1		0.00			493	54.7	6.09
Q7Z460	CLIP-associating protein 1 OS=Homo sapiens OX=9606 GN=CLASP1 PE=1 SV=1 - [CLAP1_HUMAN]	1.82	1	1	1	3		0.00			3.37	1.82	1	3	1538	169.3	9.03
P11169	Solute carrier family 2, facilitated glucose transporter member 3 OS=Homo sapiens OX=9606 GN=SLC2A3 PE=1 SV=1 - [G	1.81	2	1	1	1					2.37	1.81	1	1	496	53.9	7.20
Q13332	Receptor-type tyrosine-protein phosphatase S OS=Homo sapiens OX=9606 GN=PTPRS PE=1 SV=3 - [PTPRS_HUMAN]	1.80	1	2	2	2		0.00			5.44	1.80	2	2	1948	216.9	6.46
Q5T8P6	RNA-binding protein 26 OS=Homo sapiens OX=9606 GN=RBM26 PE=1 SV=3 - [RBM26_HUMAN]	1.79	1	1	1	1		0.00			3.54	1.79	1	1	1007	113.5	9.16
Q0IIM8	TBC1 domain family member 8B OS=Homo sapiens OX=9606 GN=TBC1D8B PE=1 SV=2 - [TBC8B_HUMAN]	1.79	1	1	1	2	3.49	1.79	1	2		0.00			1120	128.6	5.95
Q9BRD0	BUD13 homolog OS=Homo sapiens OX=9606 GN=BUD13 PE=1 SV=1 - [BUD13_HUMAN]	1.78	1	1	1	2		0.00			1.95	1.78	1	2	619	70.5	9.86
P13637	Sodium/potassium-transporting ATPase subunit alpha-3 OS=Homo sapiens OX=9606 GN=ATP1A3 PE=1 SV=3 - [AT1A3_HU	1.78	3	1	1	1		0.00			2.41	1.78	1	1	1013	111.7	5.38
Q5T0Z8	Uncharacterized protein C6orf132 OS=Homo sapiens OX=9606 GN=C6orf132 PE=1 SV=4 - [CF132_HUMAN]	1.77	1	1	1	1		0.00			2.63	1.77	1	1	1188	124.0	9.45
Q92834	X-linked retinitis pigmentosa GTPase regulator OS=Homo sapiens OX=9606 GN=RPGR PE=1 SV=2 - [RPGR_HUMAN]	1.76	1	1	1	1	3.20	1.76	1	1		0.00			1020	113.3	4.86
Q8N1P7	Beta/gamma crystallin domain-containing protein 2 OS=Homo sapiens OX=9606 GN=CRYBG2 PE=2 SV=2 - [CRBG2_HUMA	1.75	1	1	1	1		0.00			2.63	1.75	1	1	1661	177.8	6.05
Q9NUX5	Protection of telomeres protein 1 OS=Homo sapiens OX=9606 GN=POT1 PE=1 SV=1 - [POTE1_HUMAN]	1.74	1	1	1	1	1.79	1.74	1	1		0.00			634	71.4	6.73
Q5T749	Keratinocyte proline-rich protein OS=Homo sapiens OX=9606 GN=KPRP PE=1 SV=1 - [KPRP_HUMAN]	1.73	1	1	1	1	2.11	1.73	1	1		0.00			579	64.1	8.27
P29320	Ephrin type-A receptor 3 OS=Homo sapiens OX=9606 GN=EPHA3 PE=1 SV=2 - [EPHA3_HUMAN]	1.73	1	1	1	1	2.50	1.73	1	1		0.00			983	110.1	6.76
O00423	Echinoderm microtubule-associated protein-like 1 OS=Homo sapiens OX=9606 GN=EML1 PE=1 SV=3 - [EMAL1_HUMAN]	1.72	1	1	1	1	2.04	1.72	1	1		0.00			815	89.8	7.06
Q7Z572	Spermatogenesis-associated protein 21 OS=Homo sapiens OX=9606 GN=SPATA21 PE=2 SV=3 - [SPT21_HUMAN]	1.71	1	1	1	1	0.00	1.71	1	1		0.00			469	52.2	7.17
Q96FT7	Acid-sensing ion channel 4 OS=Homo sapiens OX=9606 GN=ASIC4 PE=1 SV=2 - [ASIC4_HUMAN]	1.70	1	1	1	1	2.09	1.70	1	1		0.00			647	70.1	7.83
Q8IZJ1	Netrin receptor UNC5B OS=Homo sapiens OX=9606 GN=UNC5B PE=1 SV=2 - [UNC5B_HUMAN]	1.69	1	1	1	1	2.64	1.69	1	1		0.00			945	103.6	6.02
P46940	Ras GTPase-activating-like protein IQGAP1 OS=Homo sapiens OX=9606 GN=IQGAP1 PE=1 SV=1 - [IQGA1_HUMAN]	1.69	1	1	1	1		0.00			3.41	1.69	1	1	1657	189.1	6.48
O60603	Toll-like receptor 2 OS=Homo sapiens OX=9606 GN=TLR2 PE=1 SV=1 - [TLR2_HUMAN]	1.66	1	1	1	1		0.00			2.94	1.66	1	1	784	89.8	6.61
Q9H2E6	Semaphorin-6A OS=Homo sapiens OX=9606 GN=SEMA6A PE=1 SV=2 - [SEM6A_HUMAN]	1.65	1	1	1	1	2.99	1.65	1	1		0.00			1030	114.3	8.41
Q8TD43	Transient receptor potential cation channel subfamily M member 4 OS=Homo sapiens OX=9606 GN=TRPM4 PE=1 SV=1 - [	1.65	1	1	1	1		0.00			2.58	1.65	1	1	1214	134.2	8.15
P20073	Annexin A7 OS=Homo sapiens OX=9606 GN=ANXA7 PE=1 SV=3 - [ANXA7_HUMAN]	1.64	1	1	1	1	0.00	1.64	1	1		0.00			488	52.7	5.68
Q96PE3	Inositol polyphosphate-4-phosphatase type I A OS=Homo sapiens OX=9606 GN=INPP4A PE=1 SV=1 - [INP4A_HUMAN]	1.64	1	1	1	1		0.00			2.42	1.64	1	1	977	109.9	6.96
Q5HYW2	NHS-like protein 2 OS=Homo sapiens OX=9606 GN=NHSL2 PE=1 SV=2 - [NHSL2_HUMAN]	1.63	1	1	1	2		0.00			3.12	1.63	1	2	1225	133.2	7.77
Q7L1W4	Volume-regulated anion channel subunit LRRC8D OS=Homo sapiens OX=9606 GN=LRRC8D PE=1 SV=1 - [LRC8D_HUMAN]	1.63	1	1	1	1	2.37	1.63	1	1		0.00			858	98.1	7.72
P54278	Mismatch repair endonuclease PMS2 OS=Homo sapiens OX=9606 GN=PMS2 PE=1 SV=2 - [PMS2_HUMAN]	1.62	1	1	1	1	2.79	1.62	1	1		0.00			862	95.7	6.86
P55072	Transitional endoplasmic reticulum ATPase OS=Homo sapiens OX=9606 GN=VCP PE=1 SV=4 - [TERA_HUMAN]	1.61	1	1	1	1		0.00			2.39	1.61	1	1	806	89.3	5.26
Q9Y5P4	Ceramide transfer protein OS=Homo sapiens OX=9606 GN=CERT1 PE=1 SV=1 - [CERT_HUMAN]	1.60	1	1	1	1		0.00			0.00	1.60	1	1	624	70.8	5.48
O75064	DENN domain-containing protein 4B OS=Homo sapiens OX=9606 GN=DENND4B PE=1 SV=4 - [DEN4B_HUMAN]	1.60	1	1	1	1		0.00			0.00	1.60	1	1	1496	163.7	7.21
P12882	Myosin-1 OS=Homo sapiens OX=9606 GN=MYH1 PE=1 SV=3 - [MYH1_HUMAN]	1.60	1	1	2	2	2.14	0.52	1	1	3.01	1.08	1	1	1939	223.0	5.74
Q8IU80	Transmembrane protease serine 6 OS=Homo sapiens OX=9606 GN=TMPRSS6 PE=1 SV=3 - [TMPS6_HUMAN]	1.60	1	1	1	2		0.00			1.82	1.60	1	2	811	89.9	6.68
Q16795	NADH dehydrogenase [ubiquinone] 1 alpha subcomplex subunit 9, mitochondrial OS=Homo sapiens OX=9606 GN=NDUFA9	1.59	1	1	1	1	0.00	1.59	1	1		0.00			377	42.5	9.80
P03952	Plasma kallikrein OS=Homo sapiens OX=9606 GN=KLKB1 PE=1 SV=1 - [KLKB1_HUMAN]	1.57	1	1	1	3	4.37	1.57	1	2	1.88	1.57	1	1	638	71.3	8.22
O60610	Protein diaphanous homolog 1 OS=Homo sapiens OX=9606 GN=DIAPH1 PE=1 SV=2 - [DIAP1_HUMAN]	1.57	1	1	1	1		0.00			3.03	1.57	1	1	1272	141.3	5.41
Q96CV9	Optineurin OS=Homo sapiens OX=9606 GN=OPTN PE=1 SV=3 - [OPTN_HUMAN]	1.56	1	1	1	2	0.00	1.56	1	2		0.00			577	65.9	5.17
Q96M93	Adenosine deaminase domain-containing protein 1 OS=Homo sapiens OX=9606 GN=ADAD1 PE=2 SV=1 - [ADAD1_HUMAN]	1.56	1	1	1	1		0.00			1.77	1.56	1	1	576	64.4	9.01
Q9H788	SH2 domain-containing protein 4A OS=Homo sapiens OX=9606 GN=SH2D4A PE=1 SV=1 - [SH24A_HUMAN]	1.54	1	1	1	1	1.24	1.54	1	1		0.00			454	52.7	8.06
Q6PJI9	GATOR complex protein WDR59 OS=Homo sapiens OX=9606 GN=WDR59 PE=1 SV=2 - [WDR59_HUMAN]	1.54	1	1	1	1		0.00			2.58	1.54	1	1	974	109.7	7.91
P48736	Phosphatidylinositol 4,5-bisphosphate 3-kinase catalytic subunit gamma isoform OS=Homo sapiens OX=9606 GN=PIK3CG P	1.54	1	1	1	1		0.00			2.80	1.54	1	1	1102	126.4	7.50
Q96KP1	Exocyst complex component 2 OS=Homo sapiens OX=9606 GN=EXOC2 PE=1 SV=1 - [EXOC2_HUMAN]	1.52	1	1	1	1	1.91	1.52	1	1		0.00			924	104.0	6.90
Q9HAP2	MLX-interacting protein OS=Homo sapiens OX=9606 GN=MLXIP PE=1 SV=2 - [MLXIP_HUMAN]	1.52	1	1	1	1		0.00			2.14	1.52	1	1	919	101.1	8.35
O43143	Pre-mRNA-splicing factor ATP-dependent RNA helicase DHX15 OS=Homo sapiens OX=9606 GN=DHX15 PE=1 SV=2 - [DHX	1.51	1	1	1	1		0.00			1.78	1.51	1	1	795	90.9	7.46
Q75QN2	Integrator complex subunit 8 OS=Homo sapiens OX=9606 GN=INTS8 PE=1 SV=1 - [INT8_HUMAN]	1.51	1	1	1	1	0.00	1.51	1	1		0.00			995	113.0	7.05
Q5TC82	Roquin-1 OS=Homo sapiens OX=9606 GN=RC3H1 PE=1 SV=1 - [RC3H1_HUMAN]	1.50	1	1	1	1		0.00			2.32	1.50	1	1	1133	125.7	7.27
Q7Z3V4	Ubiquitin-protein ligase E3B OS=Homo sapiens OX=9606 GN=UBE3B PE=1 SV=3 - [UBE3B_HUMAN]	1.50	1	1	1	4		0.00			4.35	1.50	1	4	1068	123.0	8.19
O94921	Cyclin-dependent kinase 14 OS=Homo sapiens OX=9606 GN=CDK14 PE=1 SV=3 - [CDK14_HUMAN]	1.49	1	1	1	2	0.00	1.49	1	2		0.00			469	53.0	8.92
P55201	Peregrin OS=Homo sapiens OX=9606 GN=BRPF1 PE=1 SV=2 - [BRPF1_HUMAN]	1.48	1	1	1	1		0.00			0.00	1.48	1	1	1214	137.4	7.93
B1ANS9	WD repeat-containing protein 64 OS=Homo sapiens OX=9606 GN=WDR64 PE=2 SV=1 - [WDR64_HUMAN]	1.48	1	1	1	1		0.00			1.79	1.48	1	1	1081	123.6	8.19
Q6AHZ1	Zinc finger protein 518A OS=Homo sapiens OX=9606 GN=ZNF518A PE=1 SV=2 - [Z518A_HUMAN]	1.48	1	1	1	1		0.00			3.37	1.48	1	1	1483	166.7	9.28
Q9P241	Phospholipid-transporting ATPase VD OS=Homo sapiens OX=9606 GN=ATP10D PE=1 SV=3 - [AT10D_HUMAN]	1.47	1	1	1	1	3.40	1.47	1	1		0.00			1426	160.2	7.15
Q02388	Collagen alpha-1(VII) chain OS=Homo sapiens OX=9606 GN=COL7A1 PE=1 SV=2 - [CO7A1_HUMAN]	1.46	1	1	1	1	3.48	1.46	1	1		0.00			2944	295.0	6.27
Q8IWV2	Contactin-4 OS=Homo sapiens OX=9606 GN=CNTN4 PE=1 SV=1 - [CNTN4_HUMAN]	1.46	1	1	1	1		0.00			2.71	1.46	1	1	1026	113.4	7.47
P15144	Aminopeptidase N OS=Homo sapiens OX=9606 GN=ANPEP PE=1 SV=4 - [AMPN_HUMAN]	1.45	1	1	1	1		0.00			0.00	1.45	1	1	967	109.5	5.48
Q5VU43	Myomegalin OS=Homo sapiens OX=9606 GN=PDE4DIP PE=1 SV=3 - [MYOME_HUMAN]	1.45	1	1	1	1	3.06	1.45	1	1		0.00			2346	264.9	5.47
Q9UBZ9	DNA repair protein REV1 OS=Homo sapiens OX=9606 GN=REV1 PE=1 SV=1 - [REV1_HUMAN]	1.44	1	1	1	1		0.00			2.08	1.44	1	1	1251	138.2	8.51
Q9P2J2	Protein turtle homolog A OS=Homo sapiens OX=9606 GN=IGSF9 PE=1 SV=2 - [TUTLA_HUMAN]	1.44	1	1	1	1	1.94	1.44	1	1		0.00			1179	126.5	7.15
Q9BY89	Uncharacterized protein KIAA1671 OS=Homo sapiens OX=9606 GN=KIAA1671 PE=1 SV=2 - [K1671_HUMAN]	1.44	1	1	1	1		0.00			3.06	1.44	1	1	1806	196.6	8.47
Q68E01	Integrator complex subunit 3 OS=Homo sapiens OX=9606 GN=INTS3 PE=1 SV=1 - [INT3_HUMAN]	1.44	1	1	1	1		0.00			0.00	1.44	1	1	1043	118.0	5.80
Q7Z5K2	Wings apart-like protein homolog OS=Homo sapiens OX=9606 GN=WAPL PE=1 SV=1 - [WAPL_HUMAN]	1.43	1	1	1	1		0.00			2.79	1.43	1	1	1190	132.9	5.44
Q96SN8	CDK5 regulatory subunit-associated protein 2 OS=Homo sapiens OX=9606 GN=CDK5RAP2 PE=1 SV=5 - [CK5P2_HUMAN]	1.43	1	1	1	1	3.11	1.43	1	1		0.00			1893	214.9	5.58
Q6GQQ9	OTU domain-containing protein 7B OS=Homo sapiens OX=9606 GN=OTUD7B PE=1 SV=1 - [OTU7B_HUMAN]	1.42	1	1	1	1	0.00	1.42	1	1		0.00			843	92.5	6.71
Q9ULH0	Kinase D-interacting substrate of 220 kDa OS=Homo sapiens OX=9606 GN=KIDINS220 PE=1 SV=3 - [KDIS_HUMAN]	1.41	1	1	1	1	2.79	1.41	1	1		0.00			1771	196.4	6.62
Q9BYJ1	Hydroperoxide isomerase ALOXE3 OS=Homo sapiens OX=9606 GN=ALOXE3 PE=1 SV=1 - [LOXE3_HUMAN]	1.41	1	1	1	1	0.00	1.41	1	1		0.00			711	80.5	6.96
P50570	Dynamin-2 OS=Homo sapiens OX=9606 GN=DNM2 PE=1 SV=2 - [DYN2_HUMAN]	1.38	1	1	1	1		0.00			2.66	1.38	1	1	870	98.0	7.44
P51798	H(+)/Cl(-) exchange transporter 7 OS=Homo sapiens OX=9606 GN=CLCN7 PE=1 SV=2 - [CLCN7_HUMAN]	1.37	1	1	1	1	1.61	1.37	1	1		0.00			805	88.6	8.43
O60500	Nephrin OS=Homo sapiens OX=9606 GN=NPHS1 PE=1 SV=1 - [NPHN_HUMAN]	1.37	1	1	1	1		0.00			2.63	1.37	1	1	1241	134.7	5.64
Q32P28	Prolyl 3-hydroxylase 1 OS=Homo sapiens OX=9606 GN=P3H1 PE=1 SV=2 - [P3H1_HUMAN]	1.36	1	1	1	1	1.73	1.36	1	1		0.00			736	83.3	5.14
P52746	Zinc finger protein 142 OS=Homo sapiens OX=9606 GN=ZNF142 PE=1 SV=4 - [ZN142_HUMAN]	1.36	1	1	1	1	0.00	1.36	1	1		0.00			1687	187.8	7.91
Q6ZU69	Protein FAM205A OS=Homo sapiens OX=9606 GN=FAM205A PE=2 SV=4 - [F205A_HUMAN]	1.35	1	1	1	1		0.00			3.04	1.35	1	1	1335	148.0	8.29
P35443	Thrombospondin-4 OS=Homo sapiens OX=9606 GN=THBS4 PE=1 SV=2 - [TSP4_HUMAN]	1.35	1	1	1	1	2.29	1.35	1	1		0.00			961	105.8	4.68
Q8WWZ7	Cholesterol transporter ABCA5 OS=Homo sapiens OX=9606 GN=ABCA5 PE=1 SV=2 - [ABCA5_HUMAN]	1.34	1	1	1	1		0.00			1.74	1.34	1	1	1642	186.4	6.95

Q00653	Nuclear factor NF-kappa-B p100 subunit OS=Homo sapiens OX=9606 GN=NFKB2 PE=1 SV=4 - [NFKB2_HUMAN]	1.33	1	1	1	1		0.00			2.27	1.33	1	1	900	96.7	6.25
Q8TDJ6	DmX-like protein 2 OS=Homo sapiens OX=9606 GN=DMXL2 PE=1 SV=2 - [DMXL2_HUMAN]	1.32	1	2	2	2	4.67	1.32	2	2		0.00			3036	339.4	6.38
Q13586	Stromal interaction molecule 1 OS=Homo sapiens OX=9606 GN=STIM1 PE=1 SV=3 - [STIM1_HUMAN]	1.31	1	1	1	2		0.00			1.63	1.31	1	2	685	77.4	6.67
Q9BRC7	1-phosphatidylinositol 4,5-bisphosphate phosphodiesterase delta-4 OS=Homo sapiens OX=9606 GN=PLCD4 PE=1 SV=1 - [P	1.31	1	1	1	1		0.00			1.81	1.31	1	1	762	87.5	5.21
Q16720	Plasma membrane calcium-transporting ATPase 3 OS=Homo sapiens OX=9606 GN=ATP2B3 PE=1 SV=3 - [AT2B3_HUMAN]	1.31	1	1	1	1	2.26	1.31	1	1		0.00			1220	134.1	5.62
O60522	Tudor domain-containing protein 6 OS=Homo sapiens OX=9606 GN=TDRD6 PE=2 SV=2 - [TDRD6_HUMAN]	1.29	1	1	1	1		0.00			3.48	1.29	1	1	2096	236.4	5.25
Q9Y2K3	Myosin-15 OS=Homo sapiens OX=9606 GN=MYH15 PE=1 SV=5 - [MYH15_HUMAN]	1.28	1	1	1	1	3.27	1.28	1	1		0.00			1946	224.5	5.85
Q14409	Glycerol kinase 3 OS=Homo sapiens OX=9606 GN=GK3P PE=2 SV=2 - [GLPK3_HUMAN]	1.27	2	1	1	6	0.00	1.27	1	2	5.09	1.27	1	4	553	60.6	6.39
Q5VWQ8	Disabled homolog 2-interacting protein OS=Homo sapiens OX=9606 GN=DAB2IP PE=1 SV=2 - [DAB2P_HUMAN]	1.26	1	1	1	1	2.12	1.26	1	1		0.00			1189	131.5	8.72
Q12774	Rho guanine nucleotide exchange factor 5 OS=Homo sapiens OX=9606 GN=ARHGEF5 PE=1 SV=3 - [ARHG5_HUMAN]	1.25	1	1	1	1	3.23	1.25	1	1		0.00			1597	176.7	5.53
P09848	Lactase-phlorizin hydrolase OS=Homo sapiens OX=9606 GN=LCT PE=2 SV=3 - [LPH_HUMAN]	1.25	1	2	2	2		0.00			2.59	1.25	2	2	1927	218.4	6.34
Q9HBX8	Leucine-rich repeat-containing G-protein coupled receptor 6 OS=Homo sapiens OX=9606 GN=LGR6 PE=1 SV=3 - [LGR6_HU	1.24	1	1	1	1	2.17	1.24	1	1		0.00			967	104.2	5.72
O75147	Obscurin-like protein 1 OS=Homo sapiens OX=9606 GN=OBSL1 PE=1 SV=4 - [OBSL1_HUMAN]	1.21	1	1	1	1	2.56	1.21	1	1		0.00			1896	206.8	5.63
O60333	Kinesin-like protein KIF1B OS=Homo sapiens OX=9606 GN=KIF1B PE=1 SV=5 - [KIF1B_HUMAN]	1.21	1	1	1	1	2.36	1.21	1	1		0.00			1816	204.3	5.60
Q9UHI6	Probable ATP-dependent RNA helicase DDX20 OS=Homo sapiens OX=9606 GN=DDX20 PE=1 SV=2 - [DDX20_HUMAN]	1.21	1	1	1	1	0.00	1.21	1	1		0.00			824	92.2	6.95
Q7Z7M0	Multiple epidermal growth factor-like domains protein 8 OS=Homo sapiens OX=9606 GN=MEGF8 PE=1 SV=2 - [MEGF8_HU	1.20	1	1	1	1		0.00			3.92	1.20	1	1	2845	302.9	6.87
Q96PE1	Adhesion G protein-coupled receptor A2 OS=Homo sapiens OX=9606 GN=ADGRA2 PE=1 SV=2 - [AGRA2_HUMAN]	1.20	1	1	1	1	2.75	1.20	1	1		0.00			1338	142.6	8.44
Q8N442	Translation factor GUF1, mitochondrial OS=Homo sapiens OX=9606 GN=GUF1 PE=2 SV=1 - [GUF1_HUMAN]	1.20	1	1	1	2	0.00	1.20	1	1	1.63	1.20	1	1	669	74.3	8.59
Q9NQW1	Protein transport protein Sec31B OS=Homo sapiens OX=9606 GN=SEC31B PE=1 SV=1 - [SC31B_HUMAN]	1.19	1	1	1	1	1.69	1.19	1	1		0.00			1179	128.6	8.29
Q9Y2I9	TBC1 domain family member 30 OS=Homo sapiens OX=9606 GN=TBC1D30 PE=1 SV=2 - [TBC30_HUMAN]	1.19	1	1	1	1		0.00			1.71	1.19	1	1	924	102.7	8.29
Q8IV33	Uncharacterized protein KIAA0825 OS=Homo sapiens OX=9606 GN=KIAA0825 PE=1 SV=3 - [K0825_HUMAN]	1.18	1	1	1	1	2.42	1.18	1	1		0.00			1275	147.6	6.61
Q9NTI5	Sister chromatid cohesion protein PDS5 homolog B OS=Homo sapiens OX=9606 GN=PDS5B PE=1 SV=1 - [PDS5B_HUMAN]	1.17	1	1	1	2	2.58	1.17	1	2		0.00			1447	164.6	8.47
Q9NZN5	Rho guanine nucleotide exchange factor 12 OS=Homo sapiens OX=9606 GN=ARHGEF12 PE=1 SV=1 - [ARHGC_HUMAN]	1.17	1	1	1	1		0.00			0.00	1.17	1	1	1544	173.1	5.74
Q5D862	Filaggrin-2 OS=Homo sapiens OX=9606 GN=FLG2 PE=1 SV=1 - [FILA2_HUMAN]	1.17	1	1	1	1	0.00	1.17	1	1		0.00			2391	247.9	8.31
Q7Z6E9	E3 ubiquitin-protein ligase RBBP6 OS=Homo sapiens OX=9606 GN=RBBP6 PE=1 SV=1 - [RBBP6_HUMAN]	1.17	1	1	1	1		0.00			2.64	1.17	1	1	1792	201.4	9.64
Q5JTH9	RRP12-like protein OS=Homo sapiens OX=9606 GN=RRP12 PE=1 SV=2 - [RRP12_HUMAN]	1.16	1	1	1	1	3.08	1.16	1	1		0.00			1297	143.6	8.75
Q56NI9	N-acetyltransferase ESCO2 OS=Homo sapiens OX=9606 GN=ESCO2 PE=1 SV=1 - [ESCO2_HUMAN]	1.16	1	1	1	1		0.00			1.72	1.16	1	1	601	68.3	9.39
Q9C093	Sperm flagellar protein 2 OS=Homo sapiens OX=9606 GN=SPEF2 PE=1 SV=2 - [SPEF2_HUMAN]	1.15	1	1	1	1	2.66	1.15	1	1		0.00			1822	209.7	5.54
Q2M2H8	Probable maltase-glucoamylase 2 OS=Homo sapiens OX=9606 GN=MGAM2 PE=2 SV=3 - [MGAL_HUMAN]	1.15	1	2	2	2	2.70	0.80	1	1	1.79	0.36	1	1	2515	277.8	5.21
P27815	cAMP-specific 3',5'-cyclic phosphodiesterase 4A OS=Homo sapiens OX=9606 GN=PDE4A PE=1 SV=3 - [PDE4A_HUMAN]	1.13	1	1	1	1		0.00			1.93	1.13	1	1	886	98.1	5.21
Q00610	Clathrin heavy chain 1 OS=Homo sapiens OX=9606 GN=CLTC PE=1 SV=5 - [CLH1_HUMAN]	1.13	1	1	1	1		0.00			2.39	1.13	1	1	1675	191.5	5.69
O95980	Reversion-inducing cysteine-rich protein with Kazal motifs OS=Homo sapiens OX=9606 GN=RECK PE=1 SV=1 - [RECK_HUM	1.13	1	1	1	1		0.00			0.00	1.13	1	1	971	106.4	6.74
O75094	Slit homolog 3 protein OS=Homo sapiens OX=9606 GN=SLIT3 PE=2 SV=3 - [SLIT3_HUMAN]	1.12	1	1	1	2		0.00			7.65	1.12	1	2	1523	167.6	7.65
Q15477	Helicase SKI2W OS=Homo sapiens OX=9606 GN=SKIV2L PE=1 SV=3 - [SKIV2_HUMAN]	1.12	1	1	1	1		0.00			2.67	1.12	1	1	1246	137.7	6.06
P51790	H(+)/Cl(-) exchange transporter 3 OS=Homo sapiens OX=9606 GN=CLCN3 PE=1 SV=2 - [CLCN3_HUMAN]	1.10	1	1	1	1	1.60	1.10	1	1		0.00			818	90.9	6.28
O15020	Spectrin beta chain, non-erythrocytic 2 OS=Homo sapiens OX=9606 GN=SPTBN2 PE=1 SV=3 - [SPTN2_HUMAN]	1.09	1	1	1	2	3.14	1.09	1	2		0.00			2390	271.2	6.11
P78527	DNA-dependent protein kinase catalytic subunit OS=Homo sapiens OX=9606 GN=PRKDC PE=1 SV=3 - [PRKDC_HUMAN]	1.09	1	1	1	1	3.59	1.09	1	1		0.00			4128	468.8	7.12
Q8IUD2	ELKS/Rab6-interacting/CAST family member 1 OS=Homo sapiens OX=9606 GN=ERC1 PE=1 SV=1 - [RB6I2_HUMAN]	1.08	1	1	1	1		0.00			2.80	1.08	1	1	1116	128.0	5.97
A0A0J9YXQ4	Paraneoplastic antigen Ma6E OS=Homo sapiens OX=9606 GN=PNMA6E PE=4 SV=1 - [PMA6E_HUMAN]	1.08	1	1	1	1		0.00			1.81	1.08	1	1	647	65.1	4.63
Q69YQ0	Cytospin-A OS=Homo sapiens OX=9606 GN=SPECC1L PE=1 SV=2 - [CYTSA_HUMAN]	1.07	1	1	1	1	2.32	1.07	1	1		0.00			1117	124.5	5.72
Q92540	Protein SMG7 OS=Homo sapiens OX=9606 GN=SMG7 PE=1 SV=2 - [SMG7_HUMAN]	1.06	1	1	1	1	2.18	1.06	1	1		0.00			1137	127.2	8.72
Q96L93	Kinesin-like protein KIF16B OS=Homo sapiens OX=9606 GN=KIF16B PE=1 SV=2 - [KI16B_HUMAN]	1.06	1	1	1	1		0.00			2.48	1.06	1	1	1317	151.9	6.16
Q9P266	Junctional protein associated with coronary artery disease OS=Homo sapiens OX=9606 GN=JCAD PE=1 SV=3 - [JCAD_HUM	1.03	1	1	1	1	2.37	1.03	1	1		0.00			1359	148.3	7.09
Q9ULI1	NACHT and WD repeat domain-containing protein 2 OS=Homo sapiens OX=9606 GN=NWD2 PE=2 SV=3 - [NWD2_HUMAN]	1.03	1	1	1	1	2.88	1.03	1	1		0.00			1742	197.3	6.24
Q8NG31	Kinetochore scaffold 1 OS=Homo sapiens OX=9606 GN=KNL1 PE=1 SV=3 - [KNL1_HUMAN]	1.02	1	1	1	1	2.94	1.02	1	1		0.00			2342	265.2	5.47
Q4FZB7	Histone-lysine N-methyltransferase KMT5B OS=Homo sapiens OX=9606 GN=KMT5B PE=1 SV=5 - [KMT5B_HUMAN]	1.02	1	1	1	1	1.45	1.02	1	1		0.00			885	99.1	8.78
Q7Z745	Maestro heat-like repeat-containing protein family member 2B OS=Homo sapiens OX=9606 GN=MROH2B PE=2 SV=3 - [MR	1.01	1	1	1	1	2.56	1.01	1	1		0.00			1585	180.7	6.28
Q9ULI3	Protein HEG homolog 1 OS=Homo sapiens OX=9606 GN=HEG1 PE=1 SV=3 - [HEG1_HUMAN]	1.01	1	1	1	1	2.41	1.01	1	1		0.00			1381	147.4	6.18
Q8N139	ATP-binding cassette sub-family A member 6 OS=Homo sapiens OX=9606 GN=ABCA6 PE=1 SV=2 - [ABCA6_HUMAN]	0.99	1	1	1	1	2.84	0.99	1	1		0.00			1617	184.2	7.36
Q9HCH0	Nck-associated protein 5-like OS=Homo sapiens OX=9606 GN=NCKAP5L PE=1 SV=3 - [NCK5L_HUMAN]	0.97	1	1	1	1	1.69	0.97	1	1		0.00			1334	139.3	8.06
Q9UMN6	Histone-lysine N-methyltransferase 2B OS=Homo sapiens OX=9606 GN=KMT2B PE=1 SV=1 - [KMT2B_HUMAN]	0.96	1	1	1	1	2.53	0.96	1	1		0.00			2715	293.3	8.22
Q96MR6	Cilia- and flagella-associated protein 57 OS=Homo sapiens OX=9606 GN=CFAP57 PE=2 SV=3 - [CFA57_HUMAN]	0.96	1	1	1	1	1.62	0.96	1	1		0.00			1250	144.9	5.80
A2RUB1	Meiosis-specific coiled-coil domain-containing protein MEIOC OS=Homo sapiens OX=9606 GN=MEIOC PE=2 SV=3 - [MEIOC	0.95	1	1	1	1		0.00			0.00	0.95	1	1	952	107.5	7.12
Q9H3U1	Protein unc-45 homolog A OS=Homo sapiens OX=9606 GN=UNC45A PE=1 SV=1 - [UN45A_HUMAN]	0.95	1	1	1	1	0.00	0.95	1	1		0.00			944	103.0	6.07
Q96JB1	Dynein axonemal heavy chain 8 OS=Homo sapiens OX=9606 GN=DNAH8 PE=1 SV=2 - [DYH8_HUMAN]	0.94	1	1	1	1		0.00			4.66	0.94	1	1	4490	514.3	6.32
Q8TDM6	Disks large homolog 5 OS=Homo sapiens OX=9606 GN=DLG5 PE=1 SV=4 - [DLG5_HUMAN]	0.94	1	1	1	2		0.00			2.62	0.94	1	2	1919	213.7	7.42
Q5TCS8	Adenylate kinase 9 OS=Homo sapiens OX=9606 GN=AK9 PE=1 SV=2 - [KAD9_HUMAN]	0.94	1	1	1	1		0.00			3.01	0.94	1	1	1911	221.3	5.01
O75096	Low-density lipoprotein receptor-related protein 4 OS=Homo sapiens OX=9606 GN=LRP4 PE=1 SV=4 - [LRP4_HUMAN]	0.94	1	1	1	1	0.00	0.94	1	1		0.00			1905	211.9	5.27
Q96NU0	Contactin-associated protein-like 3B OS=Homo sapiens OX=9606 GN=CNTNAP3B PE=2 SV=3 - [CNT3B_HUMAN]	0.93	2	1	1	1		0.00			2.17	0.93	1	1	1288	140.3	7.40
Q15431	Synaptonemal complex protein 1 OS=Homo sapiens OX=9606 GN=SYCP1 PE=1 SV=2 - [SYCP1_HUMAN]	0.92	1	1	1	1		0.00			0.00	0.92	1	1	976	114.1	5.96
Q15643	Thyroid receptor-interacting protein 11 OS=Homo sapiens OX=9606 GN=TRIP11 PE=1 SV=3 - [TRIPB_HUMAN]	0.91	1	1	1	2	2.01	0.91	1	1	2.14	0.91	1	1	1979	227.4	5.26
Q9Y6Q9	Nuclear receptor coactivator 3 OS=Homo sapiens OX=9606 GN=NCOA3 PE=1 SV=1 - [NCOA3_HUMAN]	0.91	1	1	1	1		0.00			2.21	0.91	1	1	1424	155.2	7.47
P27487	Dipeptidyl peptidase 4 OS=Homo sapiens OX=9606 GN=DPP4 PE=1 SV=2 - [DPP4_HUMAN]	0.91	1	1	1	1		0.00			0.00	0.91	1	1	766	88.2	6.04
Q5VWN6	Protein TASOR 2 OS=Homo sapiens OX=9606 GN=TASOR2 PE=1 SV=1 - [TASO2_HUMAN]	0.91	1	1	1	2	3.16	0.91	1	2		0.00			2430	268.7	5.90
Q5TZA2	Rootletin OS=Homo sapiens OX=9606 GN=CROCC PE=1 SV=1 - [CROCC_HUMAN]	0.89	1	1	1	1	0.00	0.89	1	1		0.00			2017	228.4	5.50
Q3T8J9	GON-4-like protein OS=Homo sapiens OX=9606 GN=GON4L PE=1 SV=1 - [GON4L_HUMAN]	0.89	1	1	1	1	2.47	0.89	1	1		0.00			2241	248.5	5.01
O75179	Ankyrin repeat domain-containing protein 17 OS=Homo sapiens OX=9606 GN=ANKRD17 PE=1 SV=3 - [ANR17_HUMAN]	0.88	1	1	1	1	3.79	0.88	1	1		0.00			2603	274.1	6.52
Q9UQR1	Zinc finger protein 148 OS=Homo sapiens OX=9606 GN=ZNF148 PE=1 SV=2 - [ZN148_HUMAN]	0.88	1	1	1	1	0.00	0.88	1	1		0.00			794	88.9	6.48
Q14517	Protocadherin Fat 1 OS=Homo sapiens OX=9606 GN=FAT1 PE=1 SV=2 - [FAT1_HUMAN]	0.87	1	2	2	2	3.33	0.48	1	1	3.31	0.39	1	1	4588	506.0	5.00
Q07864	DNA polymerase epsilon catalytic subunit A OS=Homo sapiens OX=9606 GN=POLE PE=1 SV=5 - [DPOE1_HUMAN]	0.87	1	1	1	1		0.00			2.55	0.87	1	1	2286	261.4	6.39
P12270	Nucleoprotein TPR OS=Homo sapiens OX=9606 GN=TPR PE=1 SV=3 - [TPR_HUMAN]	0.85	1	1	1	1		0.00			3.37	0.85	1	1	2363	267.1	5.02
O15078	Centrosomal protein of 290 kDa OS=Homo sapiens OX=9606 GN=CEP290 PE=1 SV=2 - [CE290_HUMAN]	0.85	1	2	2	2	2.01	0.56	1	1	0.00	0.28	1	1	2479	290.2	5.95
Q9NZW4	Dentin sialophosphoprotein OS=Homo sapiens OX=9606 GN=DSPP PE=1 SV=2 - [DSPP_HUMAN]	0.85	1	1	1	2	0.00	0.85	1	2		0.00			1301	131.1	3.87
Q9UKA4	A-kinase anchor protein 11 OS=Homo sapiens OX=9606 GN=AKAP11 PE=1 SV=1 - [AKA11_HUMAN]	0.84	1	1	1	1		0.00			2.19	0.84	1	1	1901	210.4	5.39
Q149N8	E3 ubiquitin-protein ligase SHPRH OS=Homo sapiens OX=9606 GN=SHPRH PE=1 SV=2 - [SHPRH_HUMAN]	0.83	1	1	1	1		0.00			0.00	0.83	1	1	1683	193.0	7.47
Q9Y4G6	Talin-2 OS=Homo sapiens OX=9606 GN=TLN2 PE=1 SV=4 - [TLN2_HUMAN]	0.83	1	1	1	1	3.89	0.83	1	1		0.00			2542	271.4	5.57
P82987	ADAMTS-like protein 3 OS=Homo sapiens OX=9606 GN=ADAMTSL3 PE=1 SV=4 - [ATL3_HUMAN]	0.83	1	1	1	1		0.00			0.00	0.83	1	1	1691	188.6	7.83
P55060	Exportin-2 OS=Homo sapiens OX=9606 GN=CSE1L PE=1 SV=3 - [XPO2_HUMAN]	0.82	1	1	1	3		0.00			0.00	0.82	1	3	971	110.3	5.77
Q9NVE4	Coiled-coil domain-containing protein 87 OS=Homo sapiens OX=9606 GN=CCDC87 PE=1 SV=2 - [CCD87_HUMAN]	0.82	1	1	1	8	0.00	0.82	1	3	0.00	0.82	1	5	849	96.3	8.59
Q9P265	Disco-interacting protein 2 homolog B OS=Homo sapiens OX=9606 GN=DIP2B PE=1 SV=3 - [DIP2B_HUMAN]	0.82	1	1	1	1		0.00			2.35	0.82	1	1	1576	171.4	8.09
O43150	Arf-GAP with SH3 domain, ANK repeat and PH domain-containing protein 2 OS=Homo sapiens OX=9606 GN=ASAP2 PE=1 S	0.80	1	1	1	5	10.79	0.80	1	5		0.00			1006	111.6	6.68
P11717	Cation-independent mannose-6-phosphate receptor OS=Homo sapiens OX=9606 GN=IGF2R PE=1 SV=3 - [MPRI_HUMAN]	0.80	1	1	1	1		0.00			3.25	0.80	1	1	2491	274.2	5.94
Q6PIF6	Unconventional myosin-VIIb OS=Homo sapiens OX=9606 GN=MYO7B PE=1 SV=2 - [MYO7B_HUMAN]	0.80	1	1	1	1	2.29	0.80	1	1		0.00			2116	241.4	8.57
P10586	Receptor-type tyrosine-protein phosphatase F OS=Homo sapiens OX=9606 GN=PTPRF PE=1 SV=2 - [PTPRF_HUMAN]	0.79	1	1	1	1		0.00			2.35	0.79	1	1	1907	212.7	6.30
Q9NS87	Kinesin-like protein KIF15 OS=Homo sapiens OX=9606 GN=KIF15 PE=1 SV=1 - [KIF15_HUMAN]	0.79	1	1	1	1		0.00			0.00	0.79	1	1	1388	160.1	6.00
Q86VH2	Kinesin-like protein KIF27 OS=Homo sapiens OX=9606 GN=KIF27 PE=1 SV=1 - [KIF27_HUMAN]	0.79	1	1	1	1		0.00			1.81	0.79	1	1	1401	160.2	7.28
Q15468	SCL-interrupting locus protein OS=Homo sapiens OX=9606 GN=STIL PE=1 SV=2 - [STIL_HUMAN]	0.78	1	1	1	1	0.00	0.78	1	1		0.00			1287	142.9	6.46
Q01968	Inositol polyphosphate 5-phosphatase OCRL OS=Homo sapiens OX=9606 GN=OCRL PE=1 SV=3 - [OCRL_HUMAN]	0.78	1	1	1	1	0.00	0.78	1	1		0.00			901	104.1	6.55
Q5JPB2	Zinc finger protein 831 OS=Homo sapiens OX=9606 GN=ZNF831 PE=2 SV=4 - [ZN831_HUMAN]	0.78	1	1	1	2		0.00			2.25	0.78	1	2	1677	177.8	8.34
Q99728	BRCA1-associated RING domain protein 1 OS=Homo sapiens OX=9606 GN=BARD1 PE=1 SV=2 - [BARD1_HUMAN]	0.77	1	1	1	1		0.00			0.00	0.77	1	1	777	86.6	8.72
Q8IY92	Structure-specific endonuclease subunit SLX4 OS=Homo sapiens OX=9606 GN=SLX4 PE=1 SV=3 - [SLX4_HUMAN]	0.76	1	1	1	2		0.00			1.70	0.76	1	2	1834	199.9	6.06
Q5TCY1	Tau-tubulin kinase 1 OS=Homo sapiens OX=9606 GN=TTBK1 PE=1 SV=2 - [TTBK1_HUMAN]	0.76	1	1	1	1	2.40	0.76	1	1		0.00			1321	142.6	5.60
P42345	Serine/threonine-protein kinase mTOR OS=Homo sapiens OX=9606 GN=MTOR PE=1 SV=1 - [MTOR_HUMAN]	0.75	1	1	1	1	2.59	0.75	1	1		0.00			2549	288.7	7.17
O95996	Adenomatous polyposis coli protein 2 OS=Homo sapiens OX=9606 GN=APC2 PE=1 SV=1 - [APCL_HUMAN]	0.74	1	1	1	1	2.58	0.74	1	1		0.00			2303	243.8	8.82
P78332	RNA-binding protein 6 OS=Homo sapiens OX=9606 GN=RBM6 PE=1 SV=5 - [RBM6_HUMAN]	0.71	1	1	1	1		0.00			0.00	0.71	1	1	1123	128.6	6.32
P0C091	FRAS1-related extracellular matrix protein 3 OS=Homo sapiens OX=9606 GN=FREM3 PE=3 SV=2 - [FREM3_HUMAN]	0.70	1	1	1	1	0.00	0.70	1	1		0.00			2139	238.0	5.33
Q6BDS2	UHRF1-binding protein 1 OS=Homo sapiens OX=9606 GN=UHRF1BP1 PE=1 SV=1 - [URFB1_HUMAN]	0.69	1	1	1	1		0.00			0.00	0.69	1	1	1440	159.4	6.14
Q8IVF4	Dynein axonemal heavy chain 10 OS=Homo sapiens OX=9606 GN=DNAH10 PE=1 SV=4 - [DYH10_HUMAN]	0.69	1	1	1	3	3.61	0.69	1	1	4.12	0.69	1	2	4471	514.5	5.88
Q9Y485	DmX-like protein 1 OS=Homo sapiens OX=9606 GN=DMXL1 PE=1 SV=3 - [DMXL1_HUMAN]	0.69	1	2	2	3	1.63	0.69	2	3		0.00			3027	337.6	6.34
Q8N7Z5	Ankyrin repeat domain-containing protein 31 OS=Homo sapiens OX=9606 GN=ANKRD31 PE=2 SV=2 - [ANR31_HUMAN]	0.69	1	1	1	1		0.00			2.03	0.69	1	1	1873	210.7	6.20
Q9UPU5	Ubiquitin carboxyl-terminal hydrolase 24 OS=Homo sapiens OX=9606 GN=USP24 PE=1 SV=3 - [UBP24_HUMAN]	0.69	1	1	1	1	0.00	0.69	1	1		0.00			2620	294.2	6.14
Q07954	Prolow-density lipoprotein receptor-related protein 1 OS=Homo sapiens OX=9606 GN=LRP1 PE=1 SV=2 - [LRP1_HUMAN]	0.68	1	1	1	1	0.00	0.68	1	1		0.00			4544	504.3	5.39
P78509	Reelin OS=Homo sapiens OX=9606 GN=RELN PE=1 SV=3 - [RELN_HUMAN]	0.66	1	2	2	3	5.03	0.66	2	3		0.00			3460	388.1	5.88
Q9Y3S1	Serine/threonine-protein kinase WNK2 OS=Homo sapiens OX=9606 GN=WNK2 PE=1 SV=4 - [WNK2_HUMAN]	0.65	1	1	1	1		0.00			2.42	0.65	1	1	2297	242.5	6.13
Q9HCU4	Cadherin EGF LAG seven-pass G-type receptor 2 OS=Homo sapiens OX=9606 GN=CELSR2 PE=1 SV=1 - [CELR2_HUMAN]	0.65	1	1	1	1	2.71	0.65	1	1		0.00			2923	317.3	5.31
Q15878	Voltage-dependent R-type calcium channel subunit alpha-1E OS=Homo sapiens OX=9606 GN=CACNA1E PE=1 SV=3 - [CAC	0.65	1	1	1	1		0.00			2.41	0.65	1	1	2313	261.6	8.24
P46939	Utrophin OS=Homo sapiens OX=9606 GN=UTRN PE=1 SV=2 - [UTRN_HUMAN]	0.64	1	1	1	1		0.00			2.84	0.64	1	1	3433	394.2	5.33
Q5H9U9	Probable ATP-dependent RNA helicase DDX60-like OS=Homo sapiens OX=9606 GN=DDX60L PE=2 SV=2 - [DDX6L_HUMAN]	0.64	1	1	1	1		0.00			2.59	0.64	1	1	1706	197.5	8.29
Q9BV73	Centrosome-associated protein CEP250 OS=Homo sapiens OX=9606 GN=CEP250 PE=1 SV=2 - [CP250_HUMAN]	0.61	1	1	1	1	2.51	0.61	1	1		0.00			2442	281.0	5.02
Q6P0Q8	Microtubule-associated serine/threonine-protein kinase 2 OS=Homo sapiens OX=9606 GN=MAST2 PE=1 SV=2 - [MAST2_HU	0.61	1	1	1	1	1.61	0.61	1	1		0.00			1798	196.3	8.16
P00451	Coagulation factor VIII OS=Homo sapiens OX=9606 GN=F8 PE=1 SV=1 - [FA8_HUMAN]	0.60	1	1	1	1	3.07	0.60	1	1		0.00			2351	266.8	7.36
O75335	Liprin-alpha-4 OS=Homo sapiens OX=9606 GN=PPFIA4 PE=2 SV=3 - [LIPA4_HUMAN]	0.59	2	1	1	1		0.00			0.00	0.59	1	1	1185	134.3	6.74
Q8IVU3	Probable E3 ubiquitin-protein ligase HERC6 OS=Homo sapiens OX=9606 GN=HERC6 PE=1 SV=2 - [HERC6_HUMAN]	0.59	1	1	1	2	3.68	0.59	1	2		0.00			1022	115.1	7.96
B5MCY1	Tudor domain-containing protein 15 OS=Homo sapiens OX=9606 GN=TDRD15 PE=2 SV=1 - [TDR15_HUMAN]	0.57	1	1	1	1		0.00			1.65	0.57	1	1	1934	221.6	7.03
P46100	Transcriptional regulator ATRX OS=Homo sapiens OX=9606 GN=ATRX PE=1 SV=5 - [ATRX_HUMAN]	0.56	1	1	1	1	0.00	0.56	1	1		0.00			2492	282.4	6.58
Q9Y283	Inversin OS=Homo sapiens OX=9606 GN=INVS PE=1 SV=2 - [INVS_HUMAN]	0.56	1	1	1	2		0.00			0.00	0.56	1	2	1065	117.8	9.35
Q9H254	Spectrin beta chain, non-erythrocytic 4 OS=Homo sapiens OX=9606 GN=SPTBN4 PE=1 SV=2 - [SPTN4_HUMAN]	0.55	1	1	1	1	1.75	0.55	1	1		0.00			2564	288.8	6.01
Q5CZC0	Fibrous sheath-interacting protein 2 OS=Homo sapiens OX=9606 GN=FSIP2 PE=2 SV=4 - [FSIP2_HUMAN]	0.54	1	1	1	1	3.69	0.54	1	1		0.00			6907	780.1	6.71
O15417	Trinucleotide repeat-containing gene 18 protein OS=Homo sapiens OX=9606 GN=TNRC18 PE=1 SV=3 - [TNC18_HUMAN]	0.51	1	1	1	1		0.00			0.00	0.51	1	1	2968	314.3	8.70
Q6V1P9	Protocadherin-23 OS=Homo sapiens OX=9606 GN=DCHS2 PE=2 SV=2 - [PCD23_HUMAN]	0.50	1	1	1	1		0.00			2.16	0.50	1	1	3371	370.0	4.77
Q8TE73	Dynein axonemal heavy chain 5 OS=Homo sapiens OX=9606 GN=DNAH5 PE=1 SV=3 - [DYH5_HUMAN]	0.50	1	1	1	1		0.00			3.13	0.50	1	1	4624	528.7	6.10
Q9P2D1	Chromodomain-helicase-DNA-binding protein 7 OS=Homo sapiens OX=9606 GN=CHD7 PE=1 SV=3 - [CHD7_HUMAN]	0.47	1	1	1	1	0.00	0.47	1	1		0.00			2997	335.7	6.34
Q15772	Striated muscle preferentially expressed protein kinase OS=Homo sapiens OX=9606 GN=SPEG PE=1 SV=4 - [SPEG_HUMAN	0.46	1	1	1	1		0.00			3.01	0.46	1	1	3267	354.1	8.51
Q99996	A-kinase anchor protein 9 OS=Homo sapiens OX=9606 GN=AKAP9 PE=1 SV=4 - [AKAP9_HUMAN]	0.46	1	1	1	1		0.00			2.10	0.46	1	1	3907	452.7	4.98
Q5T890	DNA excision repair protein ERCC-6-like 2 OS=Homo sapiens OX=9606 GN=ERCC6L2 PE=1 SV=2 - [ER6L2_HUMAN]	0.45	1	1	1	1		0.00			0.00	0.45	1	1	1561	177.0	8.60
O75592	E3 ubiquitin-protein ligase MYCBP2 OS=Homo sapiens OX=9606 GN=MYCBP2 PE=1 SV=4 - [MYCB2_HUMAN]	0.45	1	1	1	1		0.00			2.68	0.45	1	1	4678	513.3	7.02
Q9NU22	Midasin OS=Homo sapiens OX=9606 GN=MDN1 PE=1 SV=2 - [MDN1_HUMAN]	0.43	1	1	1	1	2.96	0.43	1	1		0.00			5596	632.4	5.68
Q03164	Histone-lysine N-methyltransferase 2A OS=Homo sapiens OX=9606 GN=KMT2A PE=1 SV=5 - [KMT2A_HUMAN]	0.43	1	1	1	1		0.00			0.00	0.43	1	1	3969	431.5	9.09
Q9HC84	Mucin-5B OS=Homo sapiens OX=9606 GN=MUC5B PE=1 SV=3 - [MUC5B_HUMAN]	0.42	1	1	1	1		0.00			3.06	0.42	1	1	5762	596.0	6.64
P21817	Ryanodine receptor 1 OS=Homo sapiens OX=9606 GN=RYR1 PE=1 SV=3 - [RYR1_HUMAN]	0.42	1	1	1	1	3.10	0.42	1	1		0.00			5038	564.8	5.30
Q2LD37	Transmembrane protein KIAA1109 OS=Homo sapiens OX=9606 GN=KIAA1109 PE=1 SV=2 - [K1109_HUMAN]	0.42	1	1	1	1	2.56	0.42	1	1		0.00			5005	555.1	6.58
Q12769	Nuclear pore complex protein Nup160 OS=Homo sapiens OX=9606 GN=NUP160 PE=1 SV=3 - [NU160_HUMAN]	0.42	1	1	1	1	0.00	0.42	1	1		0.00			1436	162.0	5.50
P13611	Versican core protein OS=Homo sapiens OX=9606 GN=VCAN PE=1 SV=3 - [CSPG2_HUMAN]	0.41	1	1	1	1	2.47	0.41	1	1		0.00			3396	372.6	4.51
P46013	Proliferation marker protein Ki-67 OS=Homo sapiens OX=9606 GN=MKI67 PE=1 SV=2 - [KI67_HUMAN]	0.40	1	1	1	1		0.00			0.00	0.40	1	1	3256	358.5	9.45
Q9P2D7	Dynein axonemal heavy chain 1 OS=Homo sapiens OX=9606 GN=DNAH1 PE=2 SV=5 - [DYH1_HUMAN]	0.38	1	1	1	1		0.00			2.57	0.38	1	1	4265	487.2	5.83
Q5T4S7	E3 ubiquitin-protein ligase UBR4 OS=Homo sapiens OX=9606 GN=UBR4 PE=1 SV=1 - [UBR4_HUMAN]	0.37	1	1	1	1		0.00			2.82	0.37	1	1	5183	573.5	6.04
O95071	E3 ubiquitin-protein ligase UBR5 OS=Homo sapiens OX=9606 GN=UBR5 PE=1 SV=2 - [UBR5_HUMAN]	0.36	1	1	1	1	1.82	0.36	1	1		0.00			2799	309.2	5.85
Q4LDE5	Sushi, von Willebrand factor type A, EGF and pentraxin domain-containing protein 1 OS=Homo sapiens OX=9606 GN=SVEF	0.36	1	1	1	1	2.37	0.36	1	1		0.00			3571	389.9	5.50
Q8WXG9	Adhesion G-protein coupled receptor V1 OS=Homo sapiens OX=9606 GN=ADGRV1 PE=1 SV=2 - [AGRV1_HUMAN]	0.36	1	1	1	1		0.00			3.00	0.36	1	1	6306	692.6	4.64
P11532	Dystrophin OS=Homo sapiens OX=9606 GN=DMD PE=1 SV=3 - [DMD_HUMAN]	0.35	1	1	1	1	2.24	0.35	1	1		0.00			3685	426.5	5.88
Q5UIP0	Telomere-associated protein RIF1 OS=Homo sapiens OX=9606 GN=RIF1 PE=1 SV=2 - [RIF1_HUMAN]	0.32	1	1	1	1	2.53	0.32	1	1		0.00			2472	274.3	5.52
O15230	Laminin subunit alpha-5 OS=Homo sapiens OX=9606 GN=LAMA5 PE=1 SV=8 - [LAMA5_HUMAN]	0.30	1	1	1	1	0.00	0.30	1	1		0.00			3695	399.5	7.02
Q7Z5P9	Mucin-19 OS=Homo sapiens OX=9606 GN=MUC19 PE=1 SV=3 - [MUC19_HUMAN]	0.29	1	1	1	1		0.00			3.03	0.29	1	1	8384	804.8	5.01
Q6ZQQ6	WD repeat-containing protein 87 OS=Homo sapiens OX=9606 GN=WDR87 PE=1 SV=3 - [WDR87_HUMAN]	0.28	1	1	1	1	0.00	0.28	1	1		0.00			2873	333.0	7.28
Q6ZS30	Neurobeachin-like protein 1 OS=Homo sapiens OX=9606 GN=NBEAL1 PE=2 SV=3 - [NBEL1_HUMAN]	0.26	1	1	1	1		0.00			0.00	0.26	1	1	2694	307.0	6.44
O60673	DNA polymerase zeta catalytic subunit OS=Homo sapiens OX=9606 GN=REV3L PE=1 SV=2 - [REV3L_HUMAN]	0.26	1	1	1	1	2.15	0.26	1	1		0.00			3130	352.6	8.47
Q8NCM8	Cytoplasmic dynein 2 heavy chain 1 OS=Homo sapiens OX=9606 GN=DYNC2H1 PE=1 SV=4 - [DYHC2_HUMAN]	0.21	1	1	1	1	1.84	0.21	1	1		0.00			4307	492.3	6.54
Q9UPN3	Microtubule-actin cross-linking factor 1, isoforms 1/2/3/5 OS=Homo sapiens OX=9606 GN=MACF1 PE=1 SV=4 - [MACF1_HU	0.18	1	1	1	1		0.00			2.43	0.18	1	1	7388	837.8	5.39
Q8WXI7	Mucin-16 OS=Homo sapiens OX=9606 GN=MUC16 PE=1 SV=3 - [MUC16_HUMAN]	0.17	1	1	1	1	3.45	0.17	1	1		0.00			14507	1518.2	5.26
Q8WZ42	Titin OS=Homo sapiens OX=9606 GN=TTN PE=1 SV=4 - [TITIN_HUMAN]	0.14	1	3	3	4	4.17	0.08	2	3	2.68	0.06	1	1	34350	3813.7	6.35
Q5VST9	Obscurin OS=Homo sapiens OX=9606 GN=OBSCN PE=1 SV=3 - [OBSCN_HUMAN]	0.13	1	1	1	1		0.00			1.49	0.13	1	1	7968	867.9	5.99

Supplementary Table 2 qPCR primers

Name	Forward primer (5' to 3')	Reverse primer (5' to 3')
human CCL1	CTCATTTGCGGAGCAAGAGAT	GCCTCTGAACCCATCCAACTG
human CCL2	CCCCAGTCACCTGCTGTTAT	TGGAATCCTGAACCCACTTC
human CCL4	CTTTTCTTACACCGCGAGGA	GCTTGCTTCTTTTGGTTTGG
human CCL5	ATCCTCATTGCTACTGCCCTC	GCCACTGGTGTAGAAATACTCC
human COX2	TAAGTGCGATTGTACCCGGAC	TTTGTAGCCATAGTCAGCATTGT
human CXCL1	AGGGAATTCACCCCAAGAAC	ACTATGGGGGATGCAGGATT
human CXCL2	CCCATGGTTAAGAAAATCATCG	CTTCAGGAACAGCCACCAAT
human IL-10	TCAAGGCGCATGTGAACTCC	GATGTCAAACTCACTCATGGCT
human IL-1β	CCACAGACCTTCCAGGAGAATG	GTGCAGTTCAGTGATCGTACAGG
human IL-6	ACTCACCTCTTCAGAACGAATTG	CCATCTTTGGAAGGTTCAGGTTG
human IL-8	CTGTGTGAAGGTGCAGTTTTGCC	CTCAGCCCTCTTCAAAAACTTCTCC
human TBP	GATAAGAGAGCCACGAACCAC	CAAGAACTTAGCTGGAAAACCC
human TNF-α	CTCTTCTGCCTGCTGCACTTTG	ATGGGCTACAGGCTTGTCACTC
mouse Ccl2	TTAAAAACCTGGATCGGAACCAA	GCATTAGCTTCAGATTTACGGGT
mouse Cxcl-1	CTGGGATTCACCTCAAGAACATC	CAGGGTCAAGGCAAGCCTC
mouse Cxcl-2	CCAACCACCAGGCTACAGG	GCGTCACACTCAAGCTCTG
mouse Il-1β	TGGACCTTCCAGGATGAGGACA	GTTCATCTCGGAGCCTGTAGTG
mouse Tbp	TCAAACCCAGAATTGTTCTCC	GGGGTAGATGTTTTCAAATGC
mouse Tnf-α	CCCTCACACTCAGATCATCTTCT	GCTACGACGTGGGCTACAG

Histone 2A

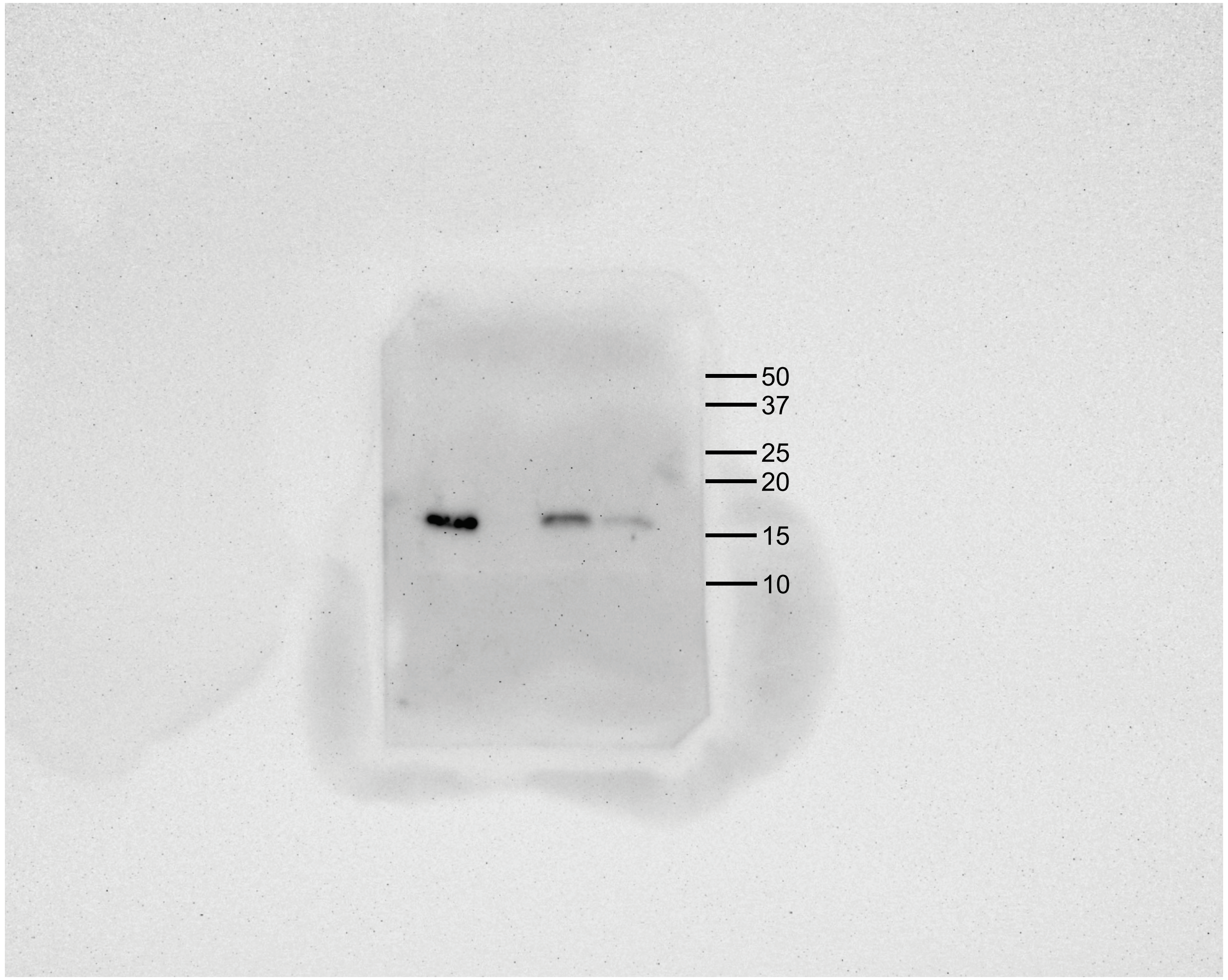

chemiluminescent

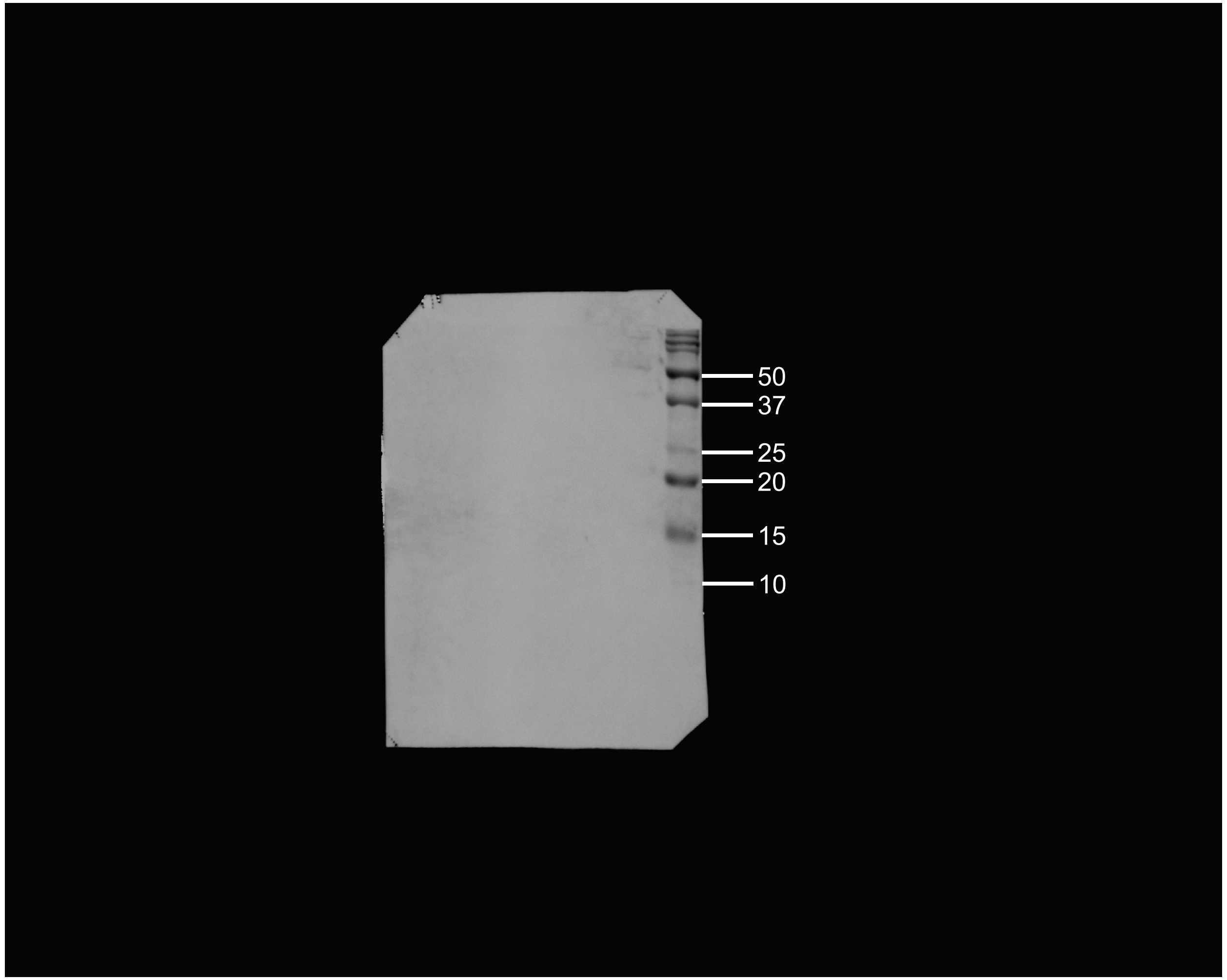

colorimetric

Supplementary Figure 1. Original full length western blots with protein standards

Histone 2B

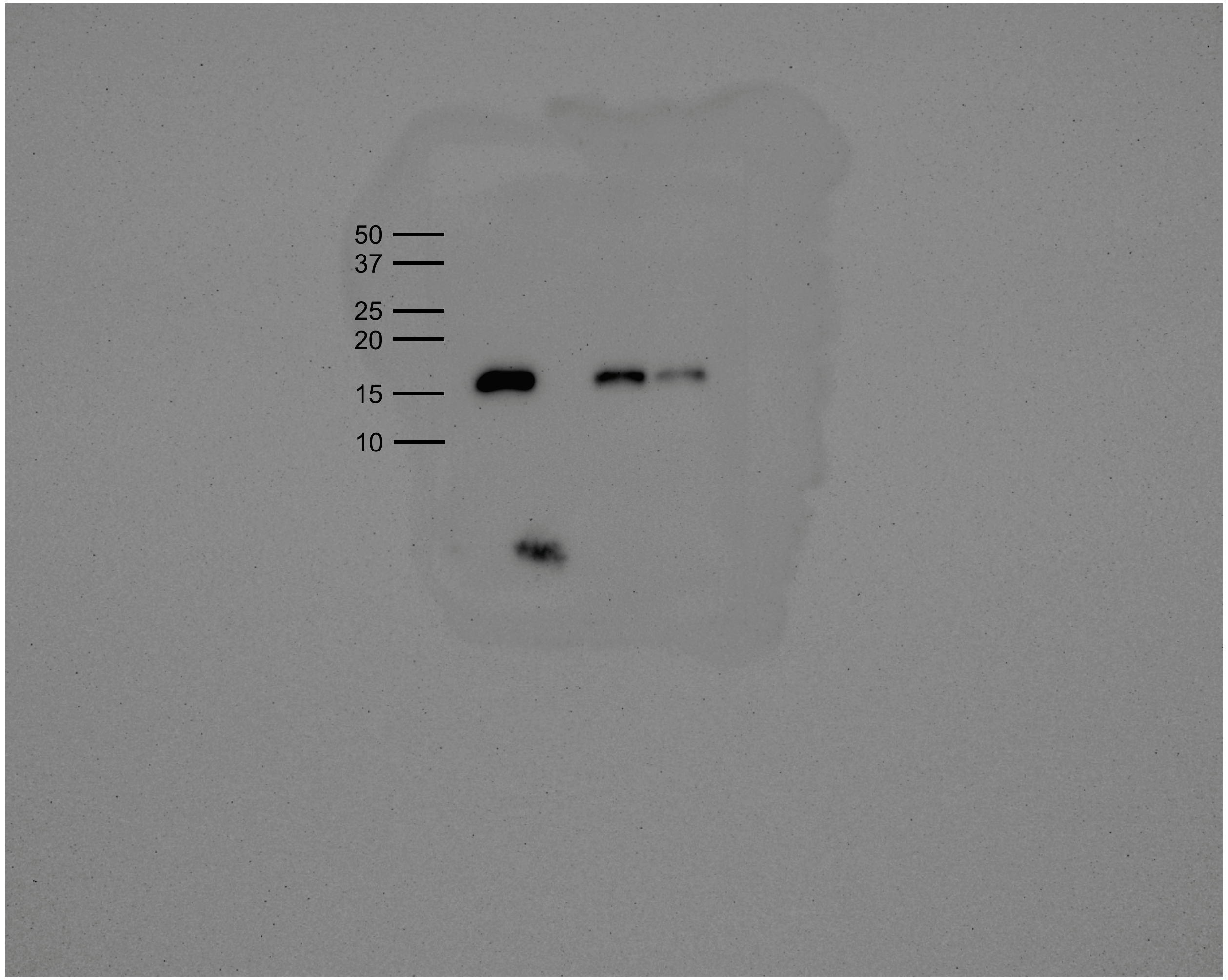

chemiluminescent

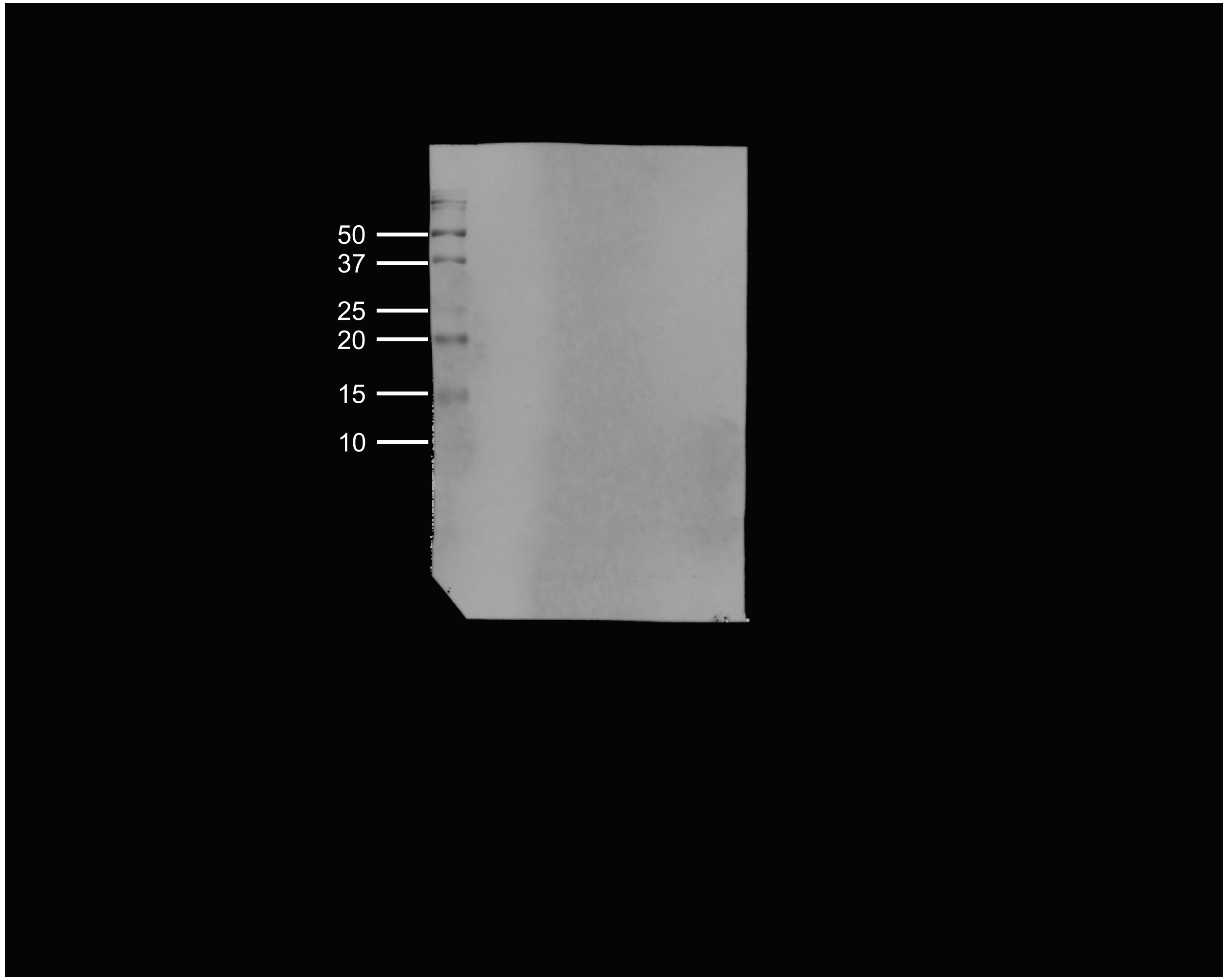

colorimetric

Supplementary Figure 1. Original full length western blots with protein standards

Histone 3

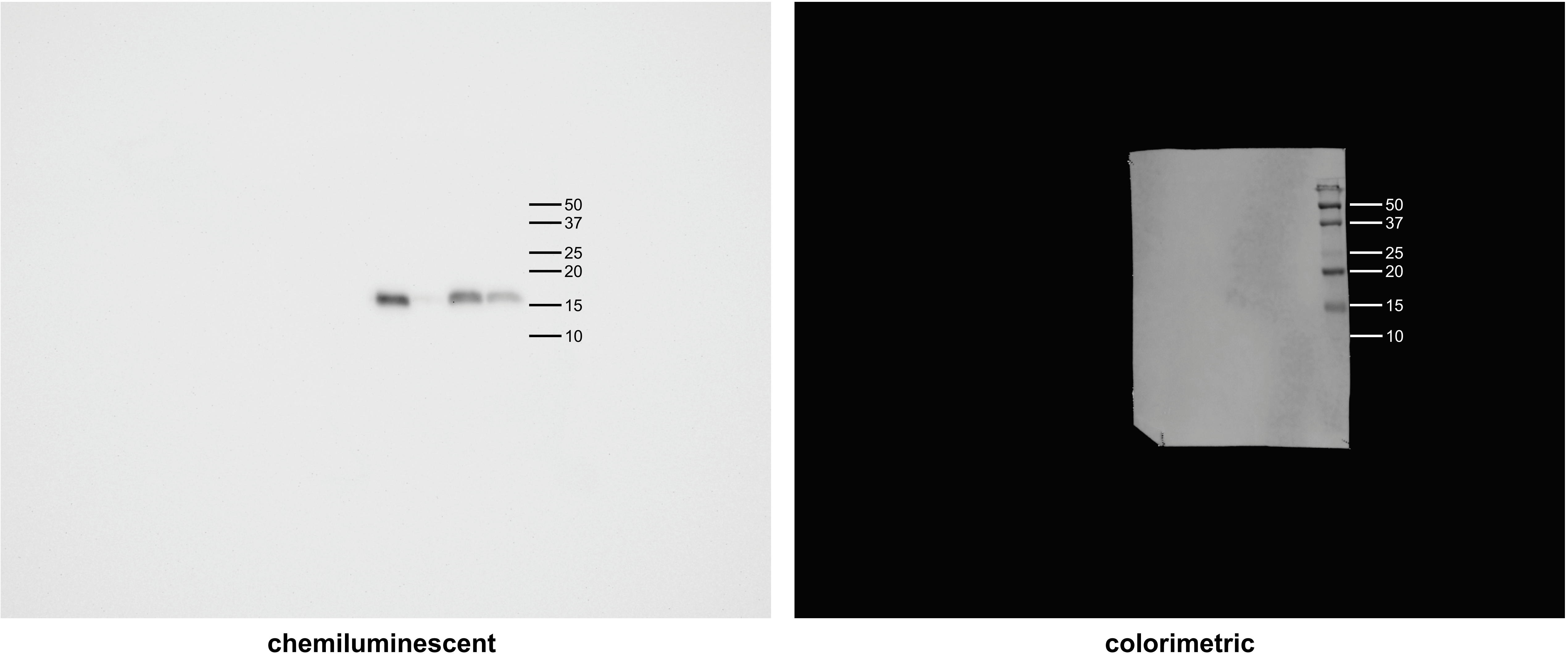

Supplementary Figure 1. Original full length western blots with protein standards

Histone 4

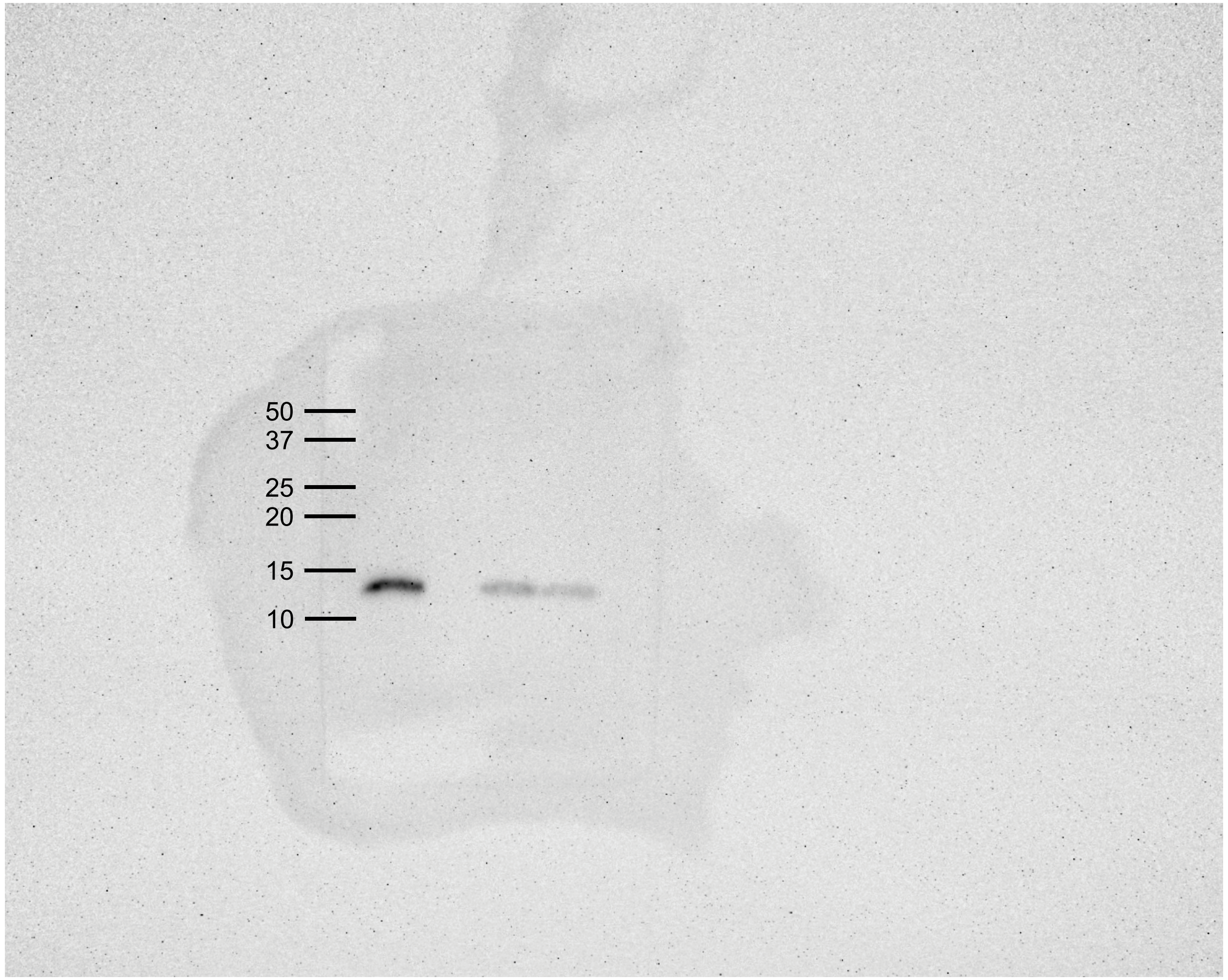

chemiluminescent

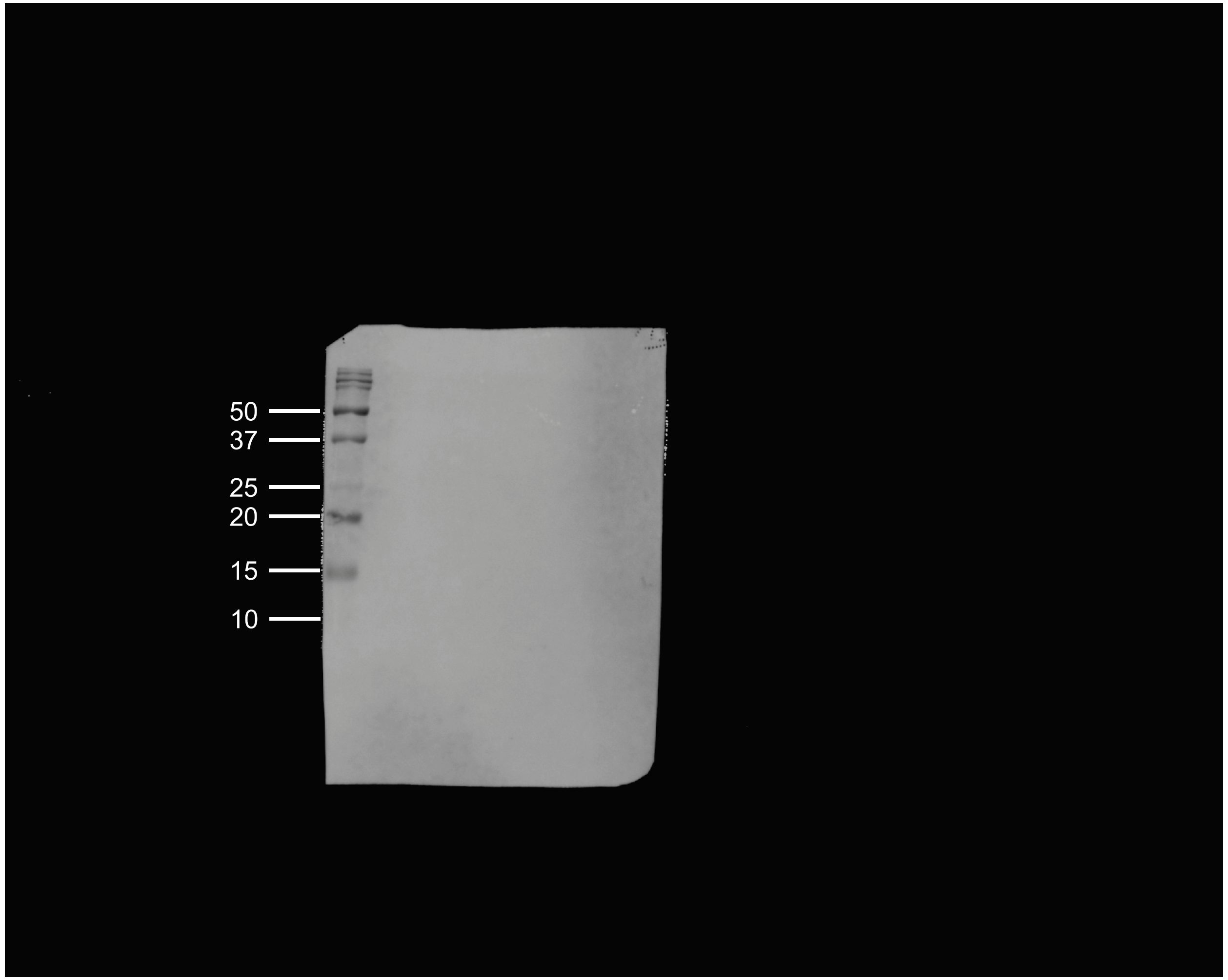

colorimetric

Supplementary Figure 1. Original full length western blots with protein standards
